# Author Correction: A sandcastle worm-inspired strategy to functionalize wet hydrogels

**DOI:** 10.1038/s41467-026-76868-8

**Published:** 2026-08-18

**Authors:** Donghui Zhang, Jingjing Liu, Qi Chen, Weinan Jiang, Yibing Wang, Jiayang Xie, Kaiqian Ma, Chao Shi, Haodong Zhang, Minzhang Chen, Jianglin Wan, Pengcheng Ma, Jingcheng Zou, Wenjing Zhang, Feng Zhou, Runhui Liu

**Affiliations:** 1 State Key Laboratory of Bioreactor Engineering, East China University of Science and Technology, Shanghai, 200237 China; 2 Key Laboratory for Ultrafine Materials of Ministry of Education, Frontiers Science Center for Materiobiology and Dynamic Chemistry, Research Center for Biomedical Materials of Ministry of Education, School of Materials Science and Engineering, East China University of Science and Technology, Shanghai, 200237 China; 3 Biomedical Nanotechnology Center, School of Biotechnology, East China University of Science and Technology, Shanghai, 200237 China; 4 State Key Laboratory of Solid Lubrication, Lanzhou Institute of Chemical Physics, Chinese Academy of Sciences, Lanzhou, 730000 China

**Keywords:** Bioinspired materials, Gels and hydrogels, Biomedical materials

Correction to: *Nature Communications* 10.1038/s41467-021-26659-0, published online 03 November 2021

In the version of this article initially published, in Fig. 6d, the top-right fluorescence image was inadvertently sourced from an incorrect file. In the Source Data, sheets named Fig. 4b and 4d should have read 5b and 5d, respectively. The figure and Source Data are updated in the HTML and PDF versions of the article. For comparison, the original Fig. 6 is available as Supplementary Information accompanying this amendment.

## Supplementary information

Original Fig. 6

