# Supplementary material for: A sandcastle worm-inspired strategy to functionalize wet hydrogels
Source: Nat Commun. 2021 Nov 3;12:6331. doi: 10.1038/s41467-021-26659-0 (PMC8566497; doi:10.1038/s41467-021-26659-0)
Supplement: Supplementary file 1 — Supplementary Information [file 41467_2021_26659_MOESM1_ESM.pdf]

Supplementary Information

A Sandcastle Worm-Inspired Strategy to Functionalize Wet Hydrogels

Donghui Zhang,[1¶] Jingjing Liu,[1¶] Qi Chen,[2] Weinan Jiang,[2] Yibing Wang,[1,3] Jiayang Xie,[2] Kaiqian Ma,[2] Chao Shi,[2] Haodong Zhang,[2] Minzhang Chen,[2] Jianglin Wan,[2] Pengcheng Ma,[2] Jingcheng Zou,[2] Wenjing Zhang,[2] Feng Zhou[4] and Runhui Liu[*,1,2]

[1]State Key Laboratory of Bioreactor Engineering, East China University of Science and Technology, Shanghai 200237, China

[2]Key Laboratory for Ultrafine Materials of Ministry of Education, Frontiers Science Center for Materiobiology and Dynamic Chemistry, Research Center for Biomedical Materials of Ministry of Education, School of Materials Science and Engineering, East China University of Science and Technology, Shanghai 200237, China

[3]Biomedical Nanotechnology Center, School of Biotechnology, East China University of Science and Technology, Shanghai 200237, China

[4]State Key Laboratory of Solid Lubrication, Lanzhou Institute of Chemical Physics, Chinese Academy of Sciences, Lanzhou 730000, China

Correspondence should be addressed to R.L. (rliu@ecust.edu.cn)

[¶]D.Z and J.L contribute equally to this manuscript

Supplementary Methods and Figures

Materials

Fmoc-Lys(Boc)-OH was purchased from Leyan®; Poly(ethyleneglycol) diacrylate (PEGDA, M_n = 2000) was obtained from JenKem®; Polyvinyl alcohol 1799 (PVA, alcoholysis degree: 98~99%) and sodium alginate (ALG, viscosity: 200-500 mPa.s) were purchased from Aladdin; All other solvents and reagents were purchased from Adamas-beta®. The water used in these experiments was obtained from a Milli-Q water purification system with a minimum resistivity of 18.2 MΩ. cm. Rink amide-MBHA resin (catalog no. 49101, 0.516 mmol/g) and 2-chlorotrityl chloride (2-CTC) resin (catalog no. 48101, 1.179 mmol/g) were purchased from GL Biochem; MLCT silicon nitride cantilever was purchased from Bruker Nano Inc. Dulbecco's modified eagle medium (DMEM) was obtained from GE Life Science; Sep-Pak C18 column were obtained from Waters. LIVE/DEAD® BacLight kit (catalog no. L13152) for LIVE/DEAD staining and phosphate buffered saline (PBS; 10 mM phosphate, 137 mM NaCl, pH 7.4) were purchased from Thermo Fisher Scientific; *Escherichia coli* (*E. coli*, ATCC 25922) and *Staphylococcus aureus* (*S. aureus*, ATCC 6538) were used for antibacterial experiments. NIH-3T3 fibroblast (3T3 ATCC CRL-1658) was obtained from the Cell Bank of Typical Culture Collection of Chinese Academy of Sciences (Shanghai, China). Rabbit anti-Vinculin/AF555 conjugated antibody was obtained from Beijing Biosynthesis Biotechnology Co., Ltd. (catalog no. bs-6640R-AF555). FITC-phalloidin and 4'-6-diamidino-2-phenylindole (DAPI) were purchased from Yeasen Biotech Co., Ltd.

Measurements and instruments

Synthesized chemicals were purified using a SepaBean machine equipped with Sepaflash columns produced by Santai Technologies Inc. in China. Ultraviolet lamp (NCSU033B, NICHIA) was from Shenzhen Walker secret technology Co., Ltd. NMR spectra were collected on an AVANCE III 400 spectrometer or an

Ascend 600 spectrometer with TopSpin software, using $CDCl_3$, DMSO-*d*6 or D_2O as the solvent. ^{1}H NMR chemical shifts were referenced to the resonance for residual protonated solvent (δ 7.26 for $CDCl_3$, 2.50 for DMSO-*d*6, 4.79 for D_2O, and 3.31 for CD_3OD). ^{13}C NMR chemical shifts were referenced to the solvent (δ 77.16 for $CDCl_3$, 39.52 for DMSO-*d*6, and 49.00 for CD_3OD). High-resolution electrospray ionization time-of-flight mass spectrometry (HRESI-MS) was acquired using a Waters XEVO G2 TOF mass spectrometer with MassLynxTM software. High-performance liquid chromatography (HPLC) analysis was carried out on a Shimadzu LC-20AR HPLC System (with LabSolutions software) equipped with a Gemini 5 μm NX-C18 column. Matrix-assisted laser desorption/ionization time-of-flight (MALDI-TOF) mass spectra were collected on an AB SCIEX 4800plus MALDI-TOF analyzer (with Data Explorer software) in reflection mode equipped with a nitrogen laser emitting at 337 nm, using 2,5-dihydroxybenzoic acid (DHB) as matrices. Ultraviolet-visible (UV-vis) absorbance spectrum was determined by UV1800 Series UV-vis spectrophotometer (Shimadzu, Japan) with UV probe software. The gel permeation chromatography (GPC) was performed on a Waters GPC instrument with Breeze 2 software, using DMF (supplemented with 10 μM LiBr) as the mobile phase on an instrument equipped with a refractive index detector (Waters 2414) and two TOSOH alpha-2500/alpha-3000 columns (particle size 7 μm) linked in series. Microplate reader (Molecular Devices SpectraMax M2 precision) was operated with SoftMax Pro software. Microimages of cell culture on polymer modified surface were characterized using a fluorescence microscope (Nikon ECLIPSE Ti-S) with NIS Elements software. The lyophilization of the hydrogel was conducted in the Labconco® FreeZone Plus 4.5 liter cascade benchtop freeze dry system.

Synthesis of Fmoc-DOPA(acetonide)-OH

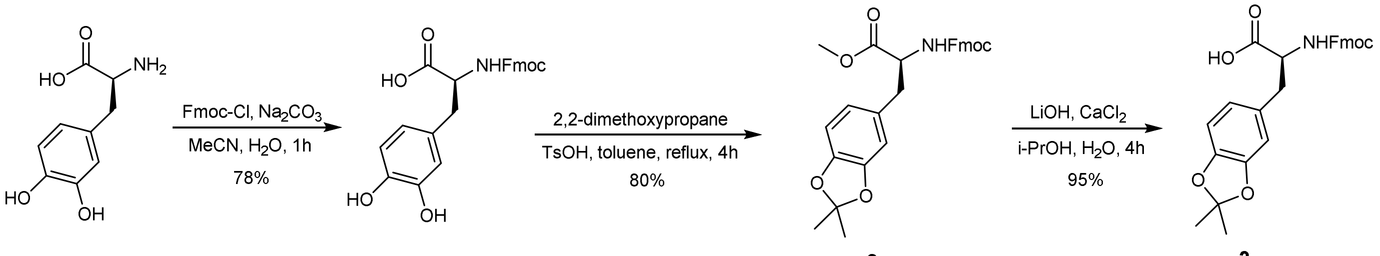

Supplementary Figure 1. Synthesis of Fmoc-DOPA(acetonide)-OH (**3**).

Synthesis of compound 1. Compound **1** was obtained using previously reported method[1]. DOPA (15 g, 76.1 mmol) was suspended in MeCN (100 mL) at 0 °C, followed by slowly addition of 10% Na_2CO_3 aqueous solution (200 mL, 188.7 mmol) into the reaction flask. After DOPA was almost dissolved, a solution of 9-fluorenylmethyl chloroformate (Fmoc-Cl; 23.6 g, 91.3 mmol) in 100 mL MeCN was slowly added into the reaction mixture via a dropping funnel under a N_2 environment. The dripping process lasts for 1 h, and the system is strictly maintained at 0 °C during this process. Then the mixture was adjusted with 6 N HCl to pH 2 at 0 °C, and then was warmed to room temperature. MeCN was removed under reduced pressure, and then the aqueous phase was extracted into CH_2Cl_2 (300 mL × 3). The combined organic phase was washed with brine (100 mL), dried over $MgSO_4$ and concentrated. The crude product was purified by silica-gel flash chromatography using 20:1 CH_2Cl_2:MeOH as the eluent to obtain compound **1** as a light brown solid (24.9 g, 78% yield). ^{1}H NMR (400 MHz, DMSO-*d6*) δ 7.88 (d, J = 7.5 Hz, 2H), 7.69-7.61 (m, 3H), 7.44-7.36 (m, 2H), 7.36-7.25 (m, 2H), 6.72-6.60 (m, 2H), 6.52 (dd, J = 8.1, 2.1 Hz, 1H), 4.27-4.12 (m, 3H), 4.07 (ddd, J = 10.1, 8.3, 4.4 Hz, 1H), 2.90 (dd, J = 13.9, 4.5 Hz, 1H), 2.69 (dd, J = 13.8, 10.2 Hz, 1H). ESI-MS m/z calcd for $C_{24}H_{21}NNaO_6$ $[M+Na]^+$: 442.1, found 441.7.

Synthesis of compound 2. Compound **1** (20 g, 47.7 mmol) was suspended in toluene (240 mL), followed by addition of 2,2-dimethoxypropane (DMP; 23.4 mL, 191 mmol) and *p*-toluenesulfonic acid (TsOH; 1.6 g, 9.5

mmol) into the reaction flask at room temperature. A Soxhlet extractor containing 40 g $CaCl_2$ was fitted on the reaction flask. The mixture was allowed to reflux for 4 h under a N_2 environment. The mixture was cooled to room temperature and poured into 200 mL EtOAc. The organic layer was washed by saturated $NaHCO_3$, brine, dried over $MgSO_4$ and concentrated. The crude product was purified by silica-gel flash chromatography using 4:1 PE:EtOAc as the eluent to obtain compound **2** as a white solid (18.1 g, 80% yield). ^{1}H NMR (400 MHz, $CDCl_3$) δ 7.78 (d, J = 7.5 Hz, 2H), 7.59 (t, J = 7.6 Hz, 2H), 7.41 (t, J = 7.4 Hz, 2H), 7.33 (m, 2H), 6.65 (d, J = 7.7 Hz, 1H), 6.51 (m, 2H), 5.37 (d, J = 8.2 Hz, 1H), 4.64 (q, J = 5.7 Hz, 1H), 4.46 (dd, J = 10.6, 7.1 Hz, 1H), 4.36 (dd, J = 10.5, 7.1 Hz, 1H), 4.24 (t, J = 7.0 Hz, 1H), 3.76 (s, 3H), 3.04 (t, J = 5.2 Hz, 2H), 1.67 (s, 6H). ^{13}C NMR (100 MHz, $CDCl_3$) δ 172.05, 155.65, 147.72, 146.65, 143.93, 143.81, 141.36, 128.65, 127.77, 127.14, 125.20, 125.14, 121.89, 120.06, 118.03, 109.41, 108.23, 67.02, 54.95, 52.41, 47.22, 37.91, 25.92. HRESI-MS m/z calcd for $C_{28}H_{27}NNaO_6$ $[M+Na]^+$: 496.1736, found 496.1735.

Synthesis of Fmoc-DOPA(acetonide)-OH (compound 3[2]). Compound **2** (16 g, 33.8 mmol) in i-PrOH (210 mL) was mixed with a $CaCl_2$ solution (30 g in 70 mL H_2O, 270.4 mmol). The mixture was cooled to 0 °C, followed by slowly addition of a LiOH solution (1.2 g in 20 mL H_2O, 50.7 mmol) to the reaction flask. The reaction mixture was allowed to stir at room temperature for 4 h, and i-PrOH was removed under a reduced pressure to give a solid. The obtained solid was dispersed in the EtOAc (300 mL) and H_2O (200 mL) and then the mixture was adjusted to pH 2 at 0 °C using 2 N HCl. Then the aqueous phase was extracted by EtOAc (200 mL × 2). The combined organic phase was washed with brine (100 mL), dried over $MgSO_4$ and concentrated. The crude product was purified by silica-gel flash chromatography using 50:1 CH_2Cl_2:MeOH as the eluent to obtain compound **3** as a white solid (14.8 g, 95% yield). ^{1}H NMR (400 MHz, $CDCl_3$) δ 7.76 (d, J = 7.6 Hz, 2H), 7.55 (q, J = 6.9 Hz, 2H), 7.40 (t, J = 7.4 Hz, 2H), 7.31 (tdd, J = 7.4, 3.1, 1.2 Hz, 2H), 6.65 (d, J = 7.7 Hz, 1H), 6.54 (d, J = 10.5 Hz, 2H), 5.22 (d, J = 8.2 Hz, 1H), 4.65 (t, J = 6.7 Hz, 1H), 4.45

(dd, J = 10.6, 7.2 Hz, 1H), 4.36 (dd, J = 10.7, 6.9 Hz, 1H), 4.22 (t, J = 7.1 Hz, 1H), 3.07 (qd, J = 14.1, 5.6 Hz, 2H). ESI-MS m/z calcd for $C_{27}H_{25}NNaO_6$ $[M+Na]^+$: 482.2, found 481.8.

Synthesis of amine terminated DbaYKY peptide

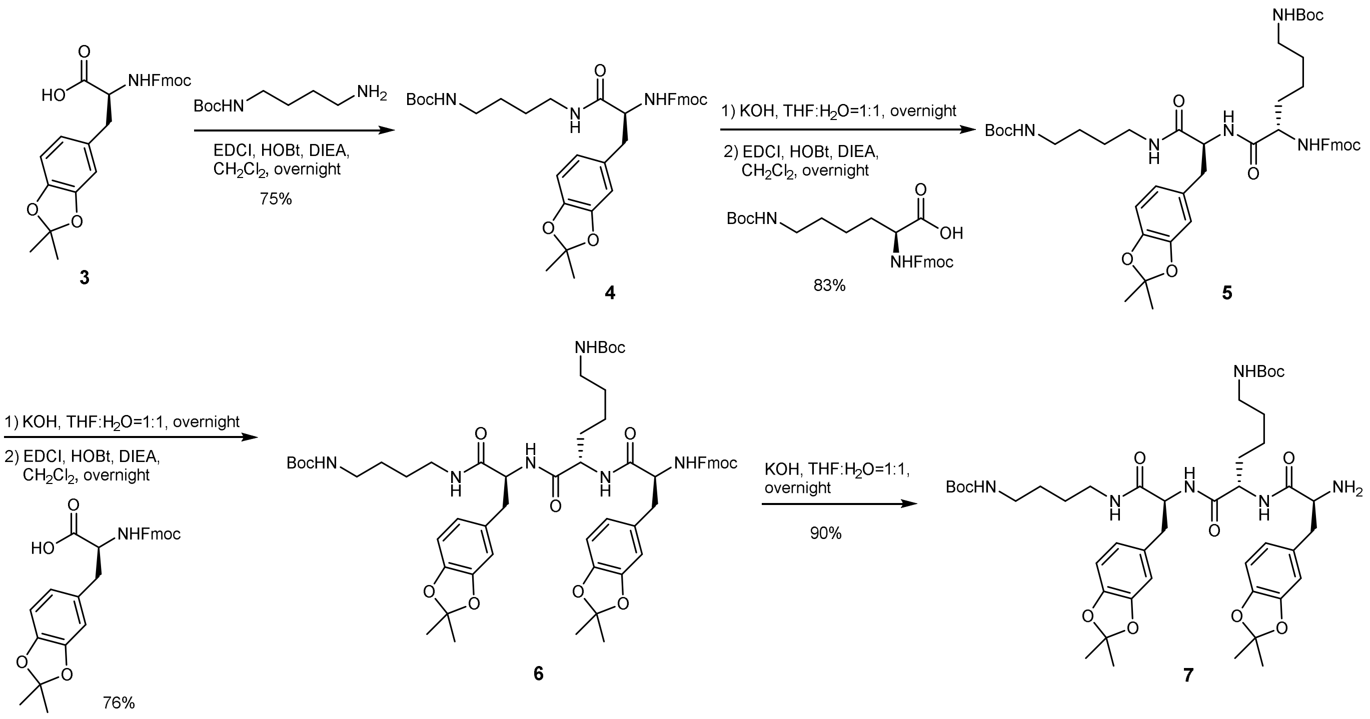

Supplementary Figure 2. Synthesis of amine terminated DbaYKY peptide (**7**).

Synthesis of compound 4. Fmoc-DOPA(acetonide)-OH (10 g, 21.8 mmol) was dissolved in CH_2Cl_2 (80 mL) at 0 °C, followed by addition of EDCI (5.0 g, 26.1 mmol), 1-hydroxybenzotriazole (HOBt, 3.5 g, 26.1 mmol) and N,N-diisopropylethylamine (DIEA, 4.5 mL, 26.1 mmol) to the mixture. Then a solution of N-(tert-butoxycarbonyl)-1,4-diaminobutane (4.9 g, 26.1 mmol) in 20 mL CH_2Cl_2 was injected into the reaction mixture under a N_2 environment. The reaction mixture was warmed to room temperature and stirred for overnight. 100 mL water was added to the reaction mixture and then the crude product was extracted into CH_2Cl_2 (400 mL × 3). The combined organic phase was washed with brine (100 mL), dried over $MgSO_4$ and concentrated to minimum amount. The crude product was purified by silica-gel flash chromatography (2:1

PE: EtOAc) to obtain compound **4** as a white solid (10.2 g, 75% yield). ^{1}H NMR (400 MHz, $CDCl_3$) δ 7.73 (d, J = 7.6 Hz, 2H), 7.52 (t, J = 7.2 Hz, 2H), 7.37 (t, J = 7.6 Hz, 2H), 7.28 (d, J = 7.2 Hz, 2H), 6.60 (t, J = 7.6 Hz, 3H), 6.42 (br, 1H), 5.79 (br, 1H), 4.75 (br, 1H), 4.40 (m, J = 10.4, 7.2 Hz, 2H), 4.25 (br, 1H), 4.16 (t, J = 6.8 Hz, 1H), 3.34-2.80 (m, 6H), 1.60 (d, J = 4.4 Hz, 6H), 1.42 (s, 9H), 1.37 (m, 4H). ^{13}C NMR (100 MHz, $CDCl_3$) δ 171.14, 156.15, 147.60, 146.37, 143.73, 141.24, 129.60, 127.72, 127.11, 125.13, 125.05, 121.85, 119.98, 117.88, 109.45, 108.11, 79.14, 67.05, 56.48, 47.06, 40.01, 39.12, 38.56, 28.44, 27.38, 26.38, 25.80, 25.79. HRESI-MS m/z calcd for $C_{36}H_{43}N_3NaO_7$ $[M+Na]^+$: 652.2999, found 652.2997.

Synthesis of compound 5. A solution of KOH (6.1 g, 108.2 mmol) in water (60 mL) was added to a solution of compound **4** (8.5 g, 13.5 mmol) in THF (60 mL) at 0 °C. The reaction mixture was stirred at room temperature for overnight. THF was removed by rotary evaporation at a reduced pressure and the water phase was extracted into EtOAc (400 mL × 3). The combined organic layer was washed with brine (100 mL), dried over $MgSO_4$ and concentrated to minimum amount. The mixture was suspended to 500 mL PE and then the suspension was sonicated for 30 min and filtered to give the primary amine intermediate as a white solid for the next step reaction without further purification. To a solution of the above crude amine intermediate (5.2 g, 12.6 mmol) and Fmoc-Lys(Boc)-OH (7.7 g, 16.4 mmol) in CH_2Cl_2 (120 mL) was added EDCI (3.2 g, 16.4 mmol), HOBt (2.2 g, 16.4 mmol) and DIEA (2.8 mL, 16.4 mmol) at 0 °C under a N_2 environment. The reaction mixture was allowed to stir at room temperature for overnight. 100 mL water was added to the reaction mixture and the crude product was extracted into CH_2Cl_2 (300 mL × 3). The combined organic phase was washed with brine (100 mL), dried over $MgSO_4$ and concentrated to minimum amount. The mixture was suspended to 500 mL EtOAc and then was sonicated for 30 min and filtered to give the compound **5** as a white solid (9.7 g, 83% yield over two steps). ^{1}H NMR (400 MHz, $CDCl_3$) δ 7.74 (d, J = 7.6 Hz, 2H), 7.58 (d, J = 6.8 Hz, 2H), 7.38 (t, J = 7.2 Hz, 2H), 7.29 (dd, J = 6.8, 3.6 Hz, 2H), 6.94 (br, 1H), 6.56 (d, J = 14.0

Hz, 4H), 6.02 (br, 1H), 4.85 (br, 2H), 4.56 (d, *J* = 6.4 Hz, 1H), 4.44-4.24 (m, 2H), 4.17 (t, *J* = 6.8 Hz, 2H), 3.34-2.78 (m, 8H), 1.84-1.62 (m, 2H), 1.56 (d, *J* = 8.8 Hz, 6H), 1.41 (d, *J* = 6.4 Hz, 24H), 1.26 (d, *J* = 11.2 Hz, 2H). ^{13}C NMR (100 MHz, $CDCl_3$) δ 171.89, 170.72, 156.82, 156.56, 156.18, 147.67, 146.41, 143.81, 143.76, 141.34, 129.69, 127.85, 127.19, 125.16, 121.73, 120.07, 117.93, 109.45, 108.11, 79.26, 79.10, 67.21, 55.42, 54.76, 47.15, 40.14, 39.55, 39.18, 37.91, 31.50, 29.76, 28.52, 28.51, 27.24, 26.44, 25.81, 25.79, 22.08. HRESI-MS m/z calcd for $C_{47}H_{63}N_5NaO_{10}$ $[M+Na]^+$: 880.4473, found 880.4474.

Synthesis of compound 6. Compound **6** was obtained using the same method as compound **5** described above. A solution of KOH (5.0 g, 88.8 mmol) in water (120 mL) was added dropwise to a solution of compound **5** (9.5 g, 11.1 mmol) in THF (120 mL) at 0 °C. The mixture was allowed to stir at room temperature for overnight. The crude product underwent work-up to give an amine crude intermediate. To a solution of above crude intermediate (6.6 g, 10.3 mmol) in CH_2Cl_2 (120 mL) was added compound **3** (5.0 g, 10.8 mmol), EDCI (3.0 g, 15.5 mmol), HOBt (2.1 g, 15.5 mmol) and DIEA (2.7 mL, 15.5 mmol) at 0 °C under a N_2 environment. The reaction mixture was allowed to stir at room temperature for overnight. After work-up, the crude product was precipitated in 500 mL EtOAc to give compound **6** as a white solid (9.0 g, 76% yield over two steps). ^{1}H NMR (400 MHz, DMSO-*d6*) δ 8.05 (d, *J* = 7.6 Hz, 1H), 7.95-7.81 (m, 4H), 7.61 (dd, *J* = 13.2, 6.8 Hz, 3H), 7.40 (td, *J* = 7.6, 3.6 Hz, 2H), 7.33-7.23 (m, , 2H), 6.82 (d, *J* = 1.2 Hz, 1H),6.78-6.62 (m, 6H), 6.59 (dd, *J* = 8.0, 1.2 Hz, 1H), 4.43-4.01 (m, 6H), 3.10-2.55 (m, 10H), 1.52-1.41 (m, 12H), 1.52-1.41 (m, 2H), 1.41-1.25 (m, 24H), 1.25-1.14 (m, 2H). ^{13}C NMR (100 MHz, DMSO-*d6*) δ 171.60, 171.17, 170.43, 155.85, 155.59, 155.53, 146.60, 145.37, 145.29, 143.76, 143.72, 140.65, 131.31, 130.64, 127.61, 127.08, 125.37, 125.27, 121.79, 121.67, 120.11, 117.45, 109.47, 109.32, 107.63, 77.35, 77.32, 65.69, 56.29, 54.13, 52.69, 46.57, 38.23, 37.71, 37.08, 31.84, 29.24, 28.28, 26.79, 26.30, 25.50, 22.60. HRESI-MS m/z calcd for $C_{59}H_{76}N_6NaO_{13}$ $[M+Na]^+$: 1099.5368, found 1099.5359.

Synthesis of amine terminated protected DbaYKY peptide (compound 7). The compound **6** (7.4 g, 6.87 mmol) was dissolved in THF (70 mL) in a 250 mL flask, and then KOH (3.1 g, 55.0 mmol) in 10 mL water was injected into the reaction flask under a N_2 protection. The mixture was stirred overnight at room temperature (rt). The crude product was extracted into CH_2Cl_2, precipitated out by adding PE, and purified by recrystallization using CH_2Cl_2: PE=1:2 to give the pure product compound **7** as a white powder (5.3 g, 90% yield). ^{1}H NMR (400 MHz, $CDCl_3$) δ 7.84 (s, 1H), 6.88 (s, 1H), 6.75-6.39 (m, 7H), 4.84 (d, J = 36.4 Hz, 2H), 4.51 (dd, J = 14, 6.8 Hz, 1H), 4.28 (d, J = 5.6 Hz, 1H), 3.51 (s, 1H), 3.33-2.80 (m, 9H), 2.70-2.50 (m, 1H), 2.40-1.95 (m, 3H), 1.78 (dd, J = 13.2, 7.2 Hz, 1H), 1.61 (d, J = 18.8 Hz, 12H), 1.41 (s, 24H). ^{13}C NMR (100 MHz, $CDCl_3$) δ 175.30, 171.49, 170.75, 156.22, 147.89, 147.54, 146.50, 146.37, 130.33, 129.76, 128.72, 121.92, 121.87, 120.03, 118.04, 117.99, 114.93, 109.49, 109.23, 108.22, 108.15, 79.11, 56.25, 54.58, 53.32, 40.43, 40.19, 39.20, 37.65, 31.48, 29.76, 29.60, 28.52, 27.23, 26.45, 25.92, 25.83, 25.80, 22.74. HRESI-MS m/z calcd for $C_{44}H_{66}N_6NaO_{11}$ $[M+H]^+$: 854.4790, found 855.4869.

Solid-phase synthesis of protected HOOC-YKY-NHFmoc and synthesis of compound 7

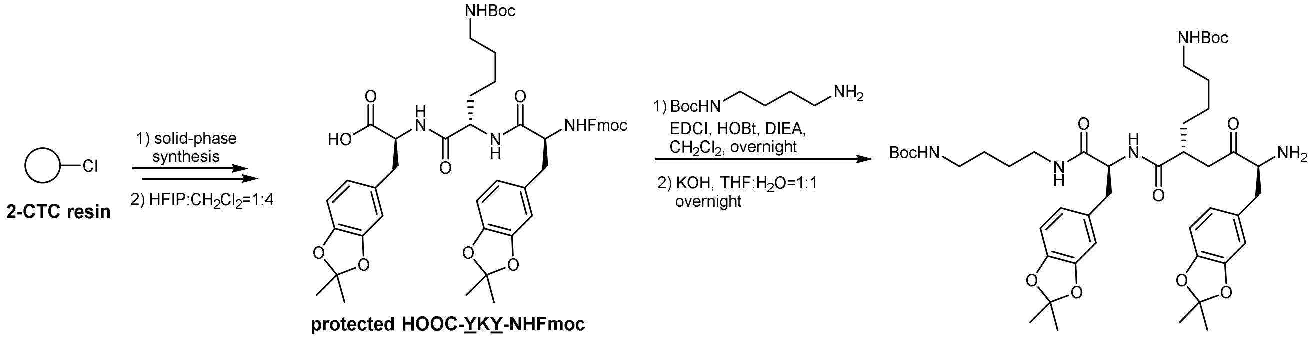

7

Supplementary Figure 3. Solid-phase synthesis of protected HOOC-YKY-NHFmoc and synthesis of compound **7**.

Synthesis of protected HOOC-YKY-NHFmoc. The protected HOOC-YKY-NHFmoc was synthesized by solid-phase synthesis on 2-CTC resins (1.179 mmol/g). The resin (46 mg, 1 equiv) was swollen in CH_2Cl_2 (2 mL) for 1 h. The conjugation with the first amino acid Y was done for 2 h with 2 mL of coumpond **3** (2 equiv) and DIEA (10 equiv) in CH_2Cl_2. The resin was washed with CH_2Cl_2 (3 × 5 mL) and DMF (3 × 5 mL). The unreacted sites on the resin were blocked twice by 7 mL a mixture of MeOH/CH_2Cl_2/DIEA (16/3/1 v/v/v) for 10 min each time. After draining the solvent, the resin was washed with CH_2Cl_2 (3 × 5 mL) and DMF (3 × 5 mL), and then treated with 20% piperidine in DMF (2 mL) for 20 min followed by washing with DMF (3 × 5 mL) and CH_2Cl_2 (3 × 5 mL). The couplings of K was done for 3 h with 2 mL of a preactivated (2 min) mixture of Fmoc protected lysine (3 equiv), DIEA (6 equiv), HOBt (3 equiv) and o-benzotriazol-1yl-N,N,N',N'-tetramethyluronium hexafluorophosphate (HBTU) (3 equiv) in DMF. The resin was washed with DMF (3 × 5 mL) and CH_2Cl_2 (3 × 5 mL). The coupling was monitored by the Kaiser test. The N-terminal Fmoc group was removed as mentioned above to allow further coupling of Y. Cleavage of protected HOOC-YKY-NHFmoc was achieved by using 2 mL of a scavenging mixture of hexafluoroisopropanol (HFIP)/CH_2Cl_2 (1/4 v/v) for 30 min. The resin was filtered and rinsed with 1 mL of CH_2Cl_2. The filtrate containing the deprotected product was concentrated under a reduced pressure and used for next step without further purification.

Synthesis of compound 7 by protected HOOC-YKY-NHFmoc. The protected HOOC-YKY-NHFmoc (61 mg) was dissolved in CH_2Cl_2 (0.3 mL), followed by addition of N-(tert-butoxycarbonyl)-1,4-diaminobutane (15.2 mg, 0.081 mmol), EDCI (15.5 mg, 0.081 mmol), HOBt (10.9 mg, 0.081 mmol) and DIEA (13.4 μL, 0.081 mmol) at 0 °C under a N_2 environment. The reaction mixture was allowed to stir at room temperature for overnight. After work-up, the crude product was precipitated out in 10 mL EtOAc to give compound **6** as a white solid (26.5 mg, 46% yield over two steps). The Fmoc protecting group in compound **6** was removed

to give compound **7**, as described above.

Synthesis of NHS-CPβ

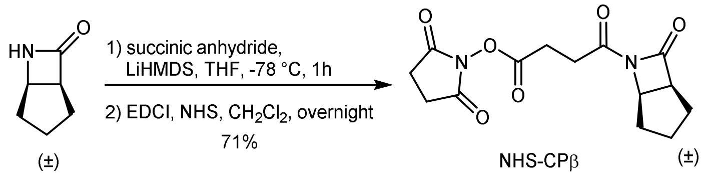

Supplementary Figure 4. Synthesis of NHS-CPβ.

The racemic mixture of β-lactam CPβ was obtained by following the previous method[3]. Lithium hexamethyldisilazide (LiHMDS) (47 mL, 1 M in THF) was added to a solution of CPβ (4.0 g, 36 mmol) in anhydrous tetrahydrofuran (THF, 400 mL) at -78 °C under a N_2 environment. After the reaction was stirred for 10 min, a solution of succinic anhydride (10.0 g, 108 mmol) in THF (20 mL) was dropwise injected to the reaction mixture. After 1 h, the reaction mixture was warmed to 0 °C and then adjusted with 1 M $NaSO_4$ to pH 2~3. After the solvent was removed by rotary evaporation at a reduced pressure, the crude product was extracted into ethyl acetate (EtOAc) (150 mL × 3) and the combined organic phase was washed with brine (50 mL), dried over $MgSO_4$, concentrated to minimum amount, and re-dissolved in anhydrous dichloromethane (CH_2Cl_2, 300 mL). Then 1-(3-dimethylaminopropyl)-3-ethylcarbodiimide hydrochloride (EDCI, 10.4 g, 54 mmol) and N-hydroxy succinimide (NHS, 6.2 g, 54 mmol) were added to the reaction and the mixture was stirred at room temperature for overnight. The reaction mixture was poured into H_2O and the crude product was extracted into CH_2Cl_2 (150 mL × 3). The combined organic phase was washed with brine (50 mL), dried over $MgSO_4$ and concentrated to minimum amount. The crude product was purified by silica-gel flash chromatography (1:1 PE:EtOAc) to give the pure product NHS-CPβ as a white solid (7.9 g, 71% yield). ^1H NMR (400 MHz, $CDCl_3$) δ 4.42 (t, J = 4.8 Hz, 1H), 3.53 (dd, J = 8.0, 4.4 Hz, 1H), 3.15-3.03 (m, 2H), 3.01-2.92 (m, 2H), 2.81 (s, 4H), 2.26 (dd, J = 13.6, 5.2 Hz, 1H), 2.09 (dd, J = 12.0, 4.8 Hz 1H),

1.93-1.83 (m, 1H), 1.67-1.50 (m, 2H), 1.49-1.36 (m, 1H). ^{13}C NMR (100 MHz, $CDCl_3$) δ 169.03, 168.20, 167.89, 56.88, 54.38, 31.13, 28.58, 25.90, 25.67, 25.22, 22.82. HRESI-MS m/z calcd for $C_{14}H_{16}N_2NaO_6$ $[M+Na]^+$: 331.0906, found 331.0907.

Synthesis of DbaYKY contained initiators

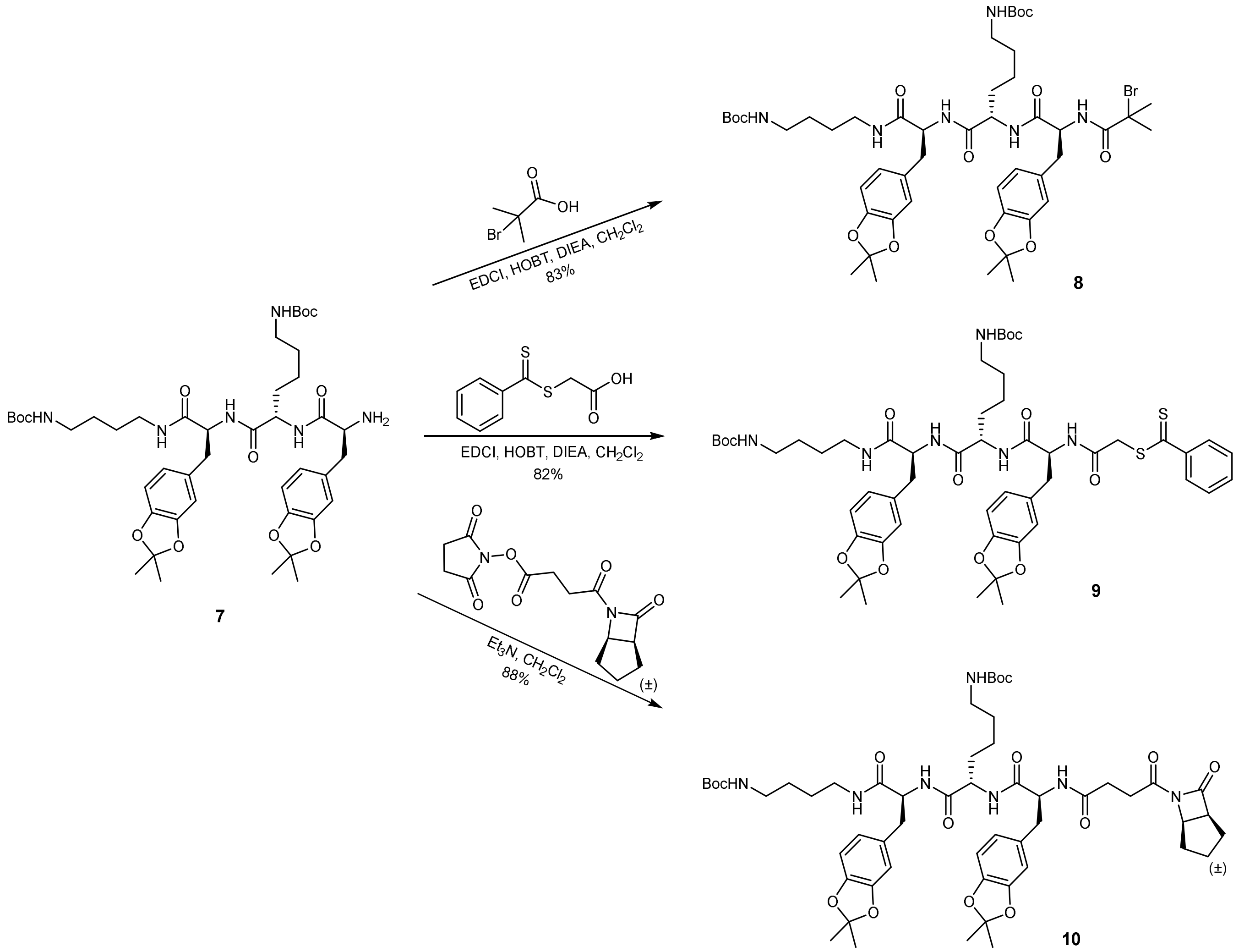

Supplementary Figure 5. Synthesis of DbaYKY containing initiators.

Synthesis of Compound 8. A solution of compound **7** (320 mg, 0.37 mmol) in CH_2Cl_2 (8 mL) was mixed with 2-bromo-2-methylpropanoic acid (75 mg, 0.45 mmol), followed by addition of EDCI (108 mg, 0.56 mmol), HOBt (76 mg, 0.56 mmol) and DIEA (97 μL, 0.56 mmol) to the reaction under a N_2 environment. After the reaction mixture was stirred at room temperature for overnight, the mixture was poured into H_2O

(50 mL) and extracted into CH_2Cl_2 (50 mL × 3). The combined organic layer was washed with brine (50 mL), dried over $MgSO_4$, and concentrated to minimum amount. The crude product was purified by silica-gel flash chromatography (97:3 CH_2Cl_2:MeOH) to give the pure compound **8** as a white solid (310 mg, 83% yield). ^{1}H NMR (600 MHz, CD_3OD) δ 6.74-6.54 (m, 6H), 4.67 (dd, J = 6.0, 3.2 Hz, 1H), 4.50 (dd, J = 9.6, 5.2 Hz, 1H), 4.43-4.34 (m, 1H), 3.27-2.83 (m, 10H), 1.86 (d, J = 12.8 Hz, 6H), 1.80-1.72 (m, 2H), 1.71-1.62 (m, 2H), 1.62-1.50 (m, 12H), 1.50-1.28 (m, 24H). ^{13}C NMR (150 MHz, $CDCl_3$) δ 172.23, 171.52, 170.90, 156.18, 156.16, 147.67, 147.19, 146.45, 146.04, 145.79, 141.09, 129.80, 129.59, 121.84, 121.80, 121.67, 121.45, 117.76, 117.73, 117.54, 117.40, 109.44, 109.20, 108.28, 108.05, 108.03, 107.57, 78.96, 55.20, 40.25, 39.12, 31.63, 28.40, 25.74, 25.72, 25.65, 25.58. HRESI-MS m/z calcd for $C_{48}H_{70}N_6O_{12}Br$ [M-H]$^-$: 1001.4235, found 1001.4238.

Synthesis of Compound 9. A solution of compound **7** (320 mg, 0.37 mmol) in CH_2Cl_2 (8 mL) was mixed with 2-(benzothioylthio)acetic acid (96 mg, 0.45 mmol), followed by addition of EDCI (108 mg, 0.56 mmol), HOBt (76 mg, 0.56 mmol) and DIEA (97 µL, 0.56 mmol) to the reaction under a N_2 environment. After the reaction mixture was stirred at room temperature for overnight, the mixture was poured into H_2O (50 mL) and extracted into CH_2Cl_2 (50 mL × 3). The combined organic layer was washed with brine (50 mL), dried over $MgSO_4$ and concentrated to minimum amount. The crude product was purified by silica-gel flash chromatography (97:3 CH_2Cl_2: MeOH) to give pure compound **9** as a red solid (322 mg, 82% yield). ^{1}H NMR (400 MHz, $CDCl_3$) δ 7.98 (d, J = 7.6 Hz, 2H), 7.69-7.28 (m, 5H), 7.10-6.75 (m, 2H), 6.72-6.37 (m, 6H), 4.79-4.02 (m, 7H), 3.33-2.73 (m, 10H), 1.81-1.54 (m, 14H), 1.43 (s, 24H), 1.17 (s, 2H). ^{13}C NMR (150 MHz, $CDCl_3$) δ 226.58, 171.00, 166.86, 156.10, 147.26, 146.02, 144.01, 128.42, 126.90, 121.77, 117.45, 109.56, 108.12, 107.78, 78.77, 39.15, 31.52, 29.64, 28.51, 28.42, 25.75, 25.66, 25.61, 22.59. HRESI-MS m/z calcd for $C_{53}H_{71}N_6O_{12}S_2$ [M-H]$^-$: 1047.4571, found 1047.4574.

Synthesis of Compound 10. Compound **7** (500 mg, 0.585 mmol) was dissolved in anhydrous CH_2Cl_2 (10 mL), followed by addition of NHS-CPβ (198.3 mg, 0.643 mmol) and triethylamine (Et_3N; 8.1 μL, 0.0585 mmol) to the reaction. After the mixture was stirred at room temperature for 3 h under a N_2 environment, the reaction mixture was poured into H_2O (100 mL) and the crude product was extracted into CH_2Cl_2 (100 mL × 3). The combined organic layer was washed with brine (50 mL), dried over $MgSO_4$ and concentrated to minimum amount. The crude product was purified by silica-gel flash chromatography (1:6 PE:EtOAc) to give compound **10** as a white solid (540 mg, 88% yield). ^{1}H NMR (400 MHz, $CDCl_3$) δ 8.04-7.30 (m, 2H), 7.12-6.73 (m, 2H), 6.57 (d, J = 29.6 Hz, 6H), 5.23-4.86 (m, 2H), 4.66 (br, 2H), 4.37 (m, 1H), 4.18 (m, 1H), 3.52 (dd, J = 6.8, 4.4 Hz, 1H), 3.33-2.68 (m, 12H), 2.62-2.36 (m, 2H), 2.23-1.95 (m, 2H), 1.94-1.69 (m, 2H), 1.66-1.49 (m, 16H), 1.39 (s, 24H). ^{13}C NMR (100 MHz, $CDCl_3$) δ 170.94, 167.89, 156.13, 156.08, 156.04, 156.00, 155.97, 147.87, 147.42, 146.61, 146.05, 121.47, 120.93, 118.15, 117.61, 117.59, 108.87, 108.27, 107.60, 79.09, 56.78, 54.23, 39.15, 31.69, 28.39, 25.77, 25.72, 25.67, 22.71. HRESI-MS m/z calcd for $C_{54}H_{77}N_7NaO_{14}$ $[M+Na]^+$: 1070.5426, found 1070.5416.

Polymer synthesis and characterization

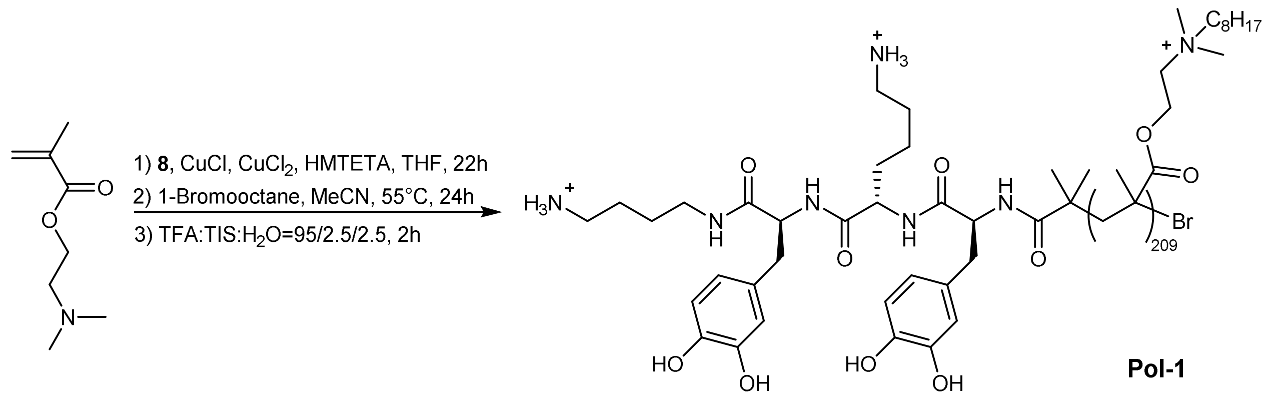

Supplementary Figure 6. Synthesis of Pol-1.

Pol-1 was obtained via atom transfer radical polymerization (ATRP) using 2-(dimethylamino)ethyl

methacrylate (DMAEMA) as the monomer and subsequent quaternization. CuCl (Aldrich; >99%) was dissolved in 12 M HCl in a flask, followed by addition of a large amount of water to precipitate out a white solid. After sediment, the supernatant was decanted and more water was added in to the flask with shaking. The solid was collected from filtration and washed alternately with ethanol and diethyl ether (Et_2O). The collected solid was dried under vacuum and stored under nitrogen for direct use as the polymerization catalyst. The polymer reaction was carried out in a N_2 purged glove box at room temperature. DMAEMA (340 μL, 2 mmol), compound **8** (10 mg, 0.01 mmol), 1,1,4,7,10,10-hexamethyltriethylenetetramine (HMTETA, 6.5 μL, 0.024 mmol) was dissolved in anhydrous THF (200 μL) in a reaction vial, followed by addition of CuCl (2.7 mg, 0.02 mmol) and $CuCl_2$ (0.4 mg, 0.004 mmol). After the reaction mixture was stirred at room temperature for 22 h, the mixture was diluted with 500 μL THF and passed through a 4 g neutral alumina column to remove the catalyst. Solvent was removed under reduced pressure and the collected residue was dissolved in THF (1 mL) followed by slow addition of cold PE (45 mL) to precipitate out the crude polymer as white solid. The polymer was collected after centrifugation, removal of the supernatant and dried under N_2 flow. After three cycles of the dissolution/precipitation procedure and drying under vacuum, the purified polymer before quanternization was collected as a white solid (150 mg, 43% yield). The obtained polymer was characterized by GPC.

The above polymer (50 mg) was dissolved in acetonitrile (1 mL) followed by addition of an excess amount 1-bromooctane (550 μL) to the polymer solution. After the reaction mixture was stirred at 55 °C for 24 h, the solvent was removed from the mixture to give an oil. This oil was dissolved in MeOH (0.5 mL), followed by slowly addition of cold Et_2O (45 mL) to precipitate out the crude polymer. The acetonide and Boc protecting groups were removed in a mixture of 95% trifluoroacetic acid (TFA), 2.5% triisopropylsilane (TIS) and 2.5% H_2O for 2 h under gentle shaking. After the solvent was evaporated under a N_2 flow, the mixture was dissolved in MeOH (0.5 mL) followed by addition of cold Et_2O (45 mL) to precipitate out a

white fluffy solid. This precipitate was collected by centrifugation and dried under a N_2 flow. After three dissolution-precipitation cycles and drying under vacuum, the final polymer was obtained as a white solid in a form of TFA salt (93 mg, 83% yield). Complete deprotection and purity of Pol-1 was confirmed by ^1H NMR.

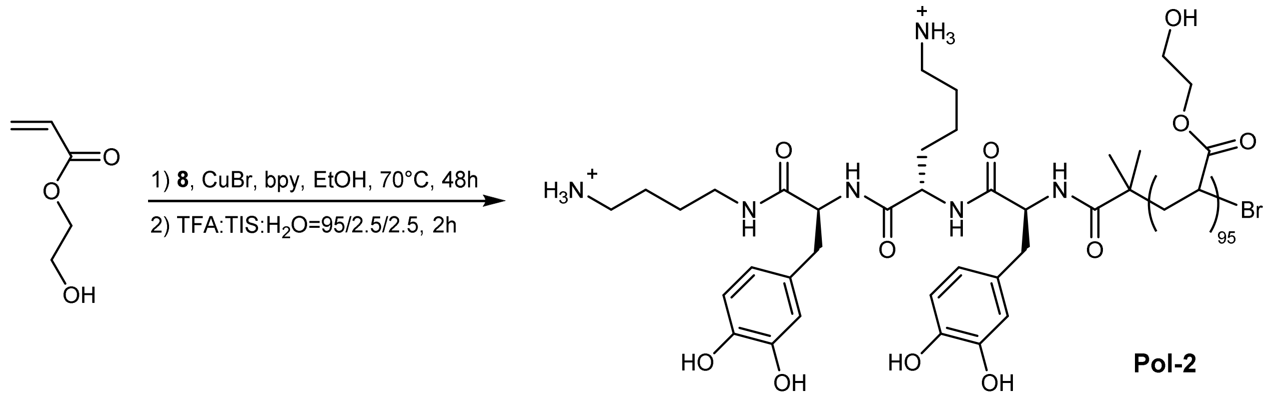

Supplementary Figure 7. Synthesis of Pol-2.

The reaction was carried out in a N_2 purged glove box at room temperature using a similar condition as mentioned above for Pol-1 synthesis. 2-hydroxyethyl acrylate (HEA, 104 μL, 1 mmol), compound **8** (5.0 mg, 0.005 mmol), 2,2'-bipyridine (bpy, 2.3 mg, 0.015 mmol) were dissovled in anhydrous ethanol (100 μL) in a vial. Then CuBr (0.7 mg, 0.005 mmol) was added to the reaction and the reaction mixture was stirred at 70 °C for 48 h. The reaction mixture was then diluted with 500 μL MeOH and passed through a 4 g neutral alumina column to remove the catalyst. The collected solution was concentrated and the resulting oil was dissolved in MeOH (0.5 mL), followed by slowly addition of cold methyl tert-butyl ether (MTBE, 45 mL) to the mixture to precipitate out the crude polymer as white solid. The polymer was collected after centrifugation and removal of the supernatant, and then the polymer was dried under a N_2 flow. After three cycles of the dissolution/precipitation procedure, the white solid was dried under vacuum. The above polymer was characterized by GPC. The protecting groups were removed by following aforementioned protocol in Pol-1 synthesis to give the final product of Pol-2 as a white solid (41 mg, 34% yield). Complete deprotection and

purity of Pol-2 was confirmed by ^{1}H NMR.

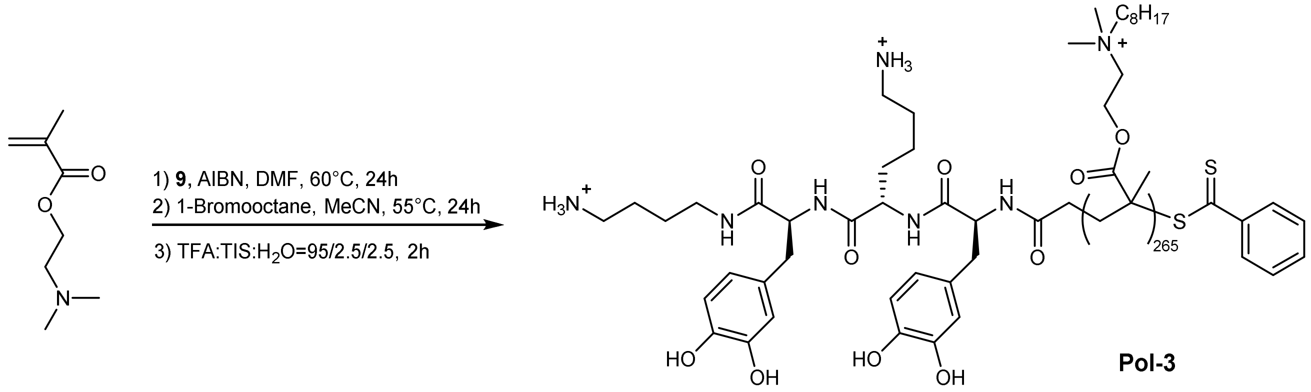

Supplementary Figure 8. Synthesis of Pol-3.

Pol-3 was obtained by reversible addition-fragmentation chain transfer polymerization (RAFT). 2,2'-Azobis(2-methylpropionitrile) (AIBN) was recrystallized from MeOH before use. The polymerization was conducted in a N_2 purged glove box to control the moisture level less than 5 ppm. DMAEMA (340 μL, 1 mmol), compound **9** (10.5 mg, 0.01 mmol), AIBN (0.82 mg, 0.005 mmol) were dissolved in anhydrous DMF (600 μL) in a reaction vial. The reaction was stirred at 60 °C for 24 h and stopped by cooling the reaction bottle in an ice-water bath and exposing to air. The crude polymer was dissolved in THF (1 mL) and precipitated out with PE (45 mL). This dissolution-precipitation process was repeated for another four cycles, followed by drying under vacuum to give the purified polymer at the protected stage as a pale pink solid (170 mg, 49% yield). The polymer at protected stage was characterized by GPC. The quaternization process of the polymer is similar to that described above for Pol-1. The polymer (50 mg) and 1-bromooctane (550 μL) was added in 1 mL acetonitrile and the mixture was stirred at 80 °C for 24 h. Quaternized PDMAEMA polymer was purified by dissolving the crude polymer in MeOH (0.5 mL) and precipitated in Et_2O (45 mL) for three times. The collected final product was dried under vacuum. The deprotection process was the same as the protocol aforementioned for Pol-1 to give final polymer of Pol-3 as a pale pink solid (95 mg, 85% yield). Complete deprotection and purity of Pol-3 was confirmed by ^{1}H NMR.

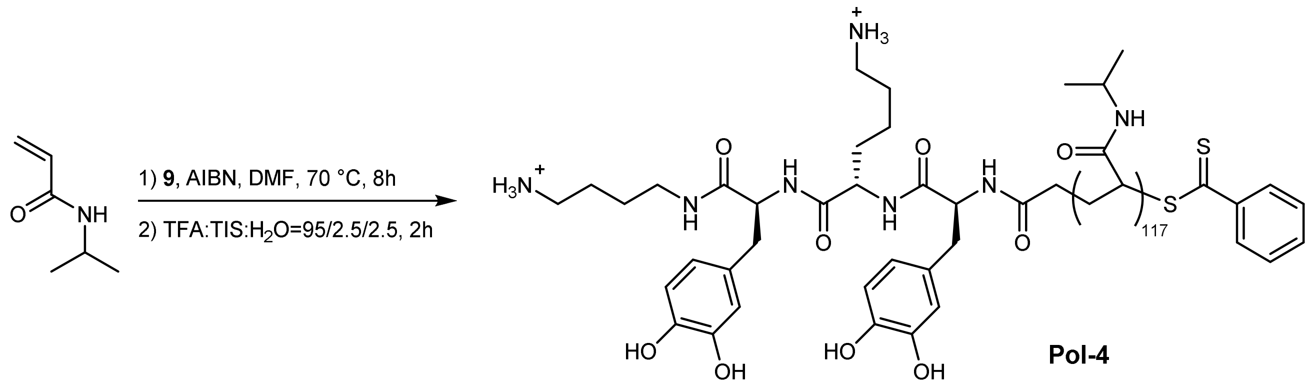

Supplementary Figure 9. Synthesis of Pol-4.

The polymerization was conducted in a N_2 purged glove box to control the moisture level less than 5 ppm. N-isopropylacrylamide (NIPAM, 45.3 mg, 0.4 mmol), compound **9** (2.1 mg, 0.002 mmol) and AIBN (0.2 mg, 0.001 mmol) was dissolved in anhydrous DMF (40 μL) in a reaction vial. The reaction mixture was stirred at 70 °C for 8 h and stopped by cooling the reaction vial in an ice-water bath and exposing to air. Polymer purification by precipitation and further removal of the protecting groups were conducted by followed the aforementioned protocol in Pol-1 synthesis to give final fully deprotected Pol-4 as a pale pink solid in the form of TFA salt (25 mg, 53% yield). The side-chain protected polymer was characterized by GPC. Complete deprotection and purity of Pol-4 was confirmed by ^{1}H NMR.

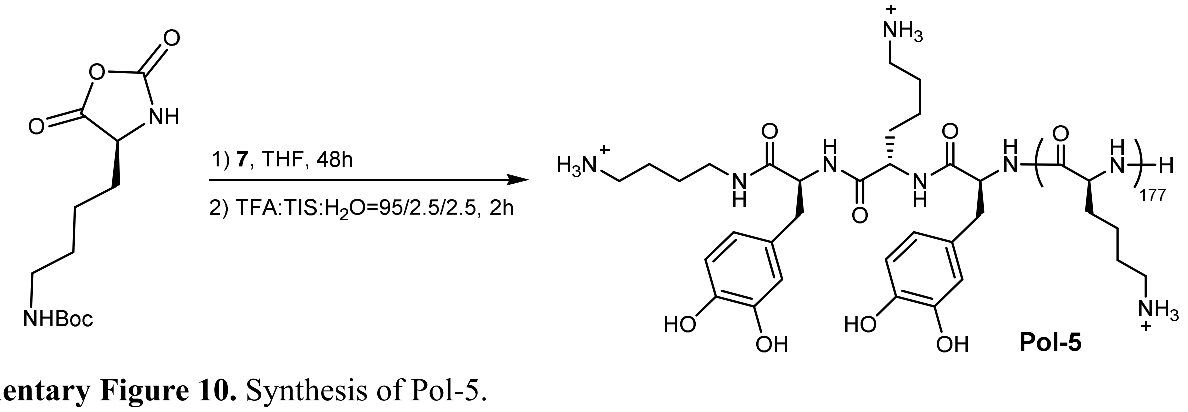

Supplementary Figure 10. Synthesis of Pol-5.

The polymerization was conducted in a N_2 purged glove box to control the moisture level less than 5 ppm. Nε-tert-butyloxycarbonyl-L-lysine N-carboxyanhydride (Boc-L-Lys-NCA) was prepared by following

previous literature[4]. To a solution of Boc-L-Lys-NCA (68.1 mg, 0.25 mmol) in anhydrous THF (1 mL) was added a solution of compound **7** (2.1 mg, 0.0025 mmol) in THF (1 mL) and the reaction mixture was stirred at room temperature for 2 days. Polymer purification by precipitation and further removal of the protecting groups were conducted by followed the aforementioned protocol in Pol-2 synthesis to give final fully deprotected Pol-5 as a white solid in the form of TFA salt (60 mg, 86% yield). The side-chain protected polymer was characterized by GPC. Complete deprotection and purity of Pol-5 was confirmed by ^{1}H NMR.

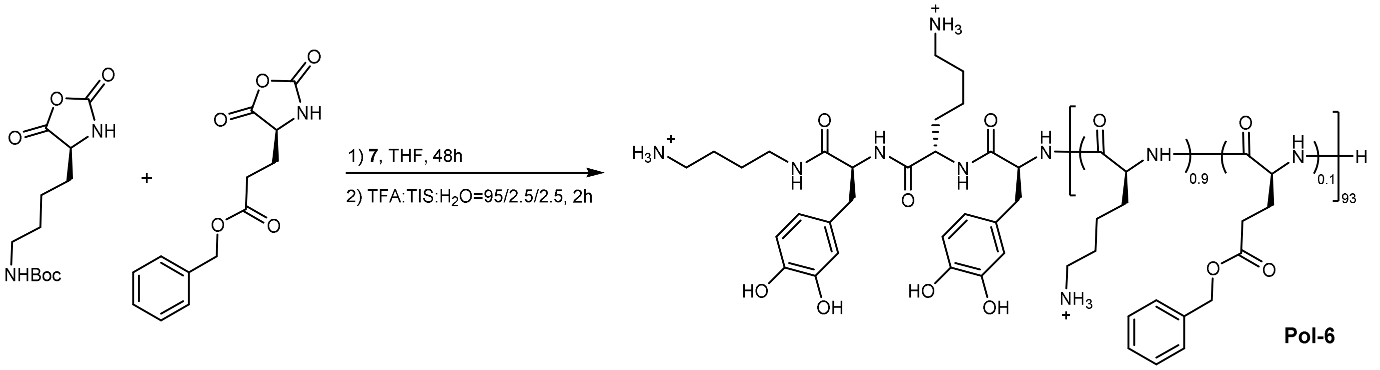

Supplementary Figure 11. Synthesis of Pol-6.

The polymerization was conducted in a N_2 purged glove box to control the moisture level less than 5 ppm. N-ε-tert-butyloxycarbonyl-L-lysine N-carboxyanhydride (Boc-L-Lys-NCA) and γ-benzyl-L-glutamate NCA (BLG-NCA) were prepared by following previous literature[4]. To a solution of Boc-L-Lys-NCA (49.0 mg, 0.18 mmol) and BLG-NCA (5.3 mg, 0.02 mmol) in anhydrous THF (1 mL) was added a solution of compound **7** (3.4 mg, 0.004 mmol) in THF (1 mL) and the reaction mixture was stirred at room temperature for 2 days. Polymer purification by precipitation and further removal of the protecting groups were conducted by followed the aforementioned protocol in Pol-1 synthesis to give final fully deprotected Pol-6 as a white solid in the form of TFA salt (51 mg, 88% yield). The side-chain protected polymer was characterized by GPC. Complete deprotection and purity of Pol-6 was confirmed by ^{1}H NMR.

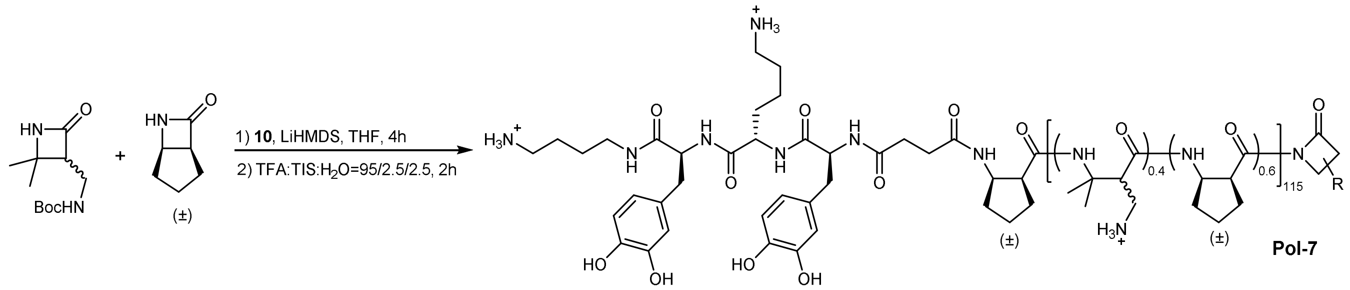

Supplementary Figure 12. Synthesis of Pol-7.

The polymerization was conducted in a N_2 purged glove box to control the moisture level less than 5 ppm. The racemic mixture of β-lactam monomers DMβ and CPβ were obtained by following previous method[3]. DMβ (27.4 mg, 0.12 mmol), CPβ (20.0 mg, 0.18 mmol) and compound **10** (3.1 mg, 0.003 mmol) were dissolved in anhydrous THF (1 mL), followed by quick addition of a solution of LiHMDS (1.3 mg, 0.0075 mmol) in THF (0.5 mL) and then the reaction mixture was stirred at room temperature for 4 h. Polymer purification by precipitation and further removal of the protecting groups were conducted by followed the aforementioned protocol in Pol-1 synthesis to give final fully deprotected Pol-7 as a white solid in the form of TFA salt (41 mg, 85% yield). The side-chain protected polymer was characterized by GPC. Complete deprotection and purity of Pol-7 was confirmed by ^{1}H NMR.

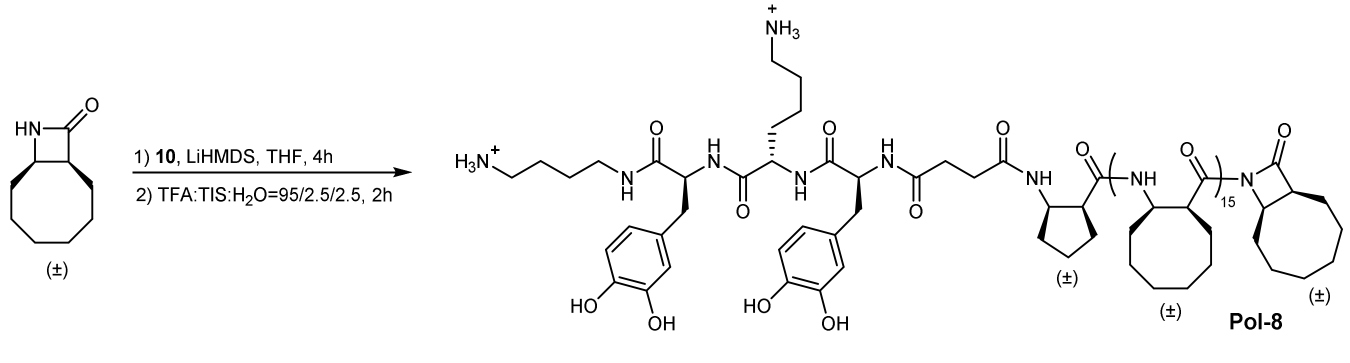

Supplementary Figure 13. Synthesis of Pol-8.

The polymerization was conducted in a N_2 purged glove box to control the moisture level less than 5 ppm. The racemic mixture of β-lactam monomers COβ was obtained by the following a previous method[5]. COβ (30.6 mg, 0.2 mmol) and compound **10** (10.5 mg, 0.01 mmol) were dissolved in anhydrous THF (1 mL)

followed by quick addition of a solution of LiHMDS (4.2 mg, 0.025 mmol) in THF (0.5 mL), and the reaction mixture was stirred at room temperature for 4 h. Polymer purification by precipitation and further removal of the protecting groups were conducted by followed the aforementioned protocol in Pol-1 synthesis to give final fully deprotected Pol-8 as a white solid in the form of TFA salt (37 mg, 90% yield). The side-chain protected polymer was characterized by GPC. Complete deprotection and purity of Pol-8 was confirmed by ^1H NMR.

Synthesis of DbaYKY-OEG8-Mal for AFM force spectroscopy measurements

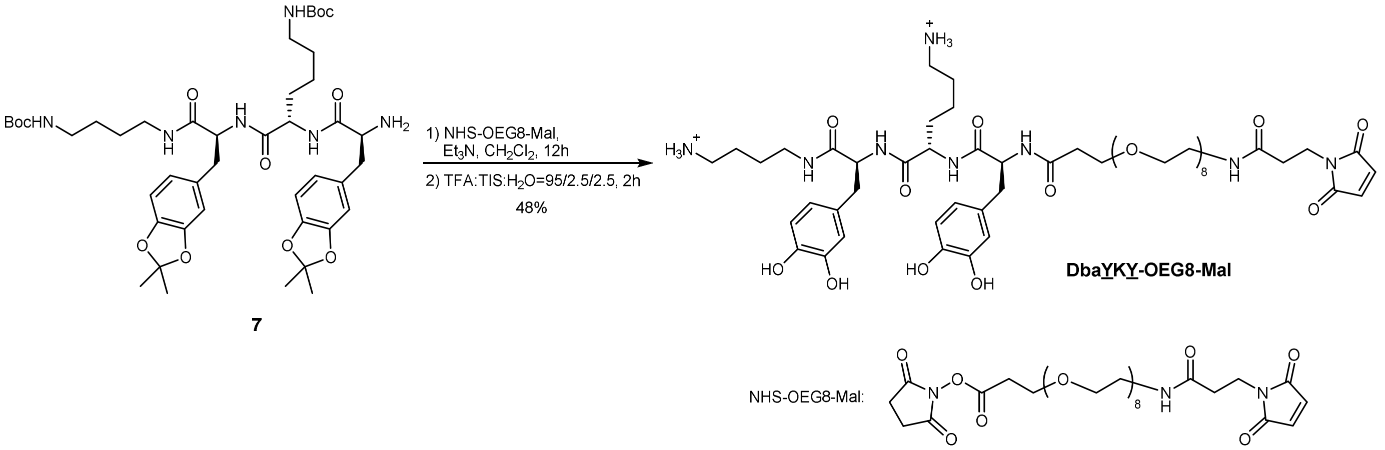

Supplementary Figure 14. Synthesis of DbaYKY-OEG8-M al.

Compound **7** (200 mg, 0.234 mmol) was dissolved in anhydrous CH_2Cl_2 (3.5 mL), followed by addition of NHS-OEG8-Mal (242 mg, 0.351 mmol) and Et_3N (49 μL, 0.351 mmol) to the reaction. After the mixture was stirred at room temperature for 12 h under a N_2 environment, the reaction mixture was diluted by CH_2Cl_2 (20 mL), and washed with saturated $NaHCO_3$ (20 mL) twice, dried over $MgSO_4$ and concentrated. The crude product at protected stage was purified by silica-gel flash chromatography using 20:1 CH_2Cl_2:MeOH as eluent. The acetonide and Boc protecting groups were removed in a 2 mL mixture of 95% TFA, 2.5% TIS and 2.5% H_2O for 2 h under gentle shaking. After the solvent was evaporated under a N_2 flow, the mixture was dissolved in MeOH (0.5 mL) followed by addition of cold methyl tert-butyl ether (45 mL) to precipitate

out a white solid. This precipitate was collected by centrifugation and dried under a N_2 flow. After three dissolution-precipitation cycles and drying under vacuum, the final product DbaYKY-OEG8-Mal was obtained as a light yellow solid in a form of TFA salt (154 mg, 48% yield). ^{1}H NMR (600 MHz, D_2O) δ 6.88-6.80 (m, 4H), 6.75 (d, J = 2.2 Hz, 2H), 6.70 (dd, J = 8.1, 2.1 Hz, 1H), 6.65 (dd, J = 8.1, 2.1 Hz, 1H), 4.50 (t, J = 7.6 Hz, 1H), 4.35 (t, J = 8.1 Hz, 1H), 4.19 (dd, J = 8.4, 6.1 Hz, 1H), 3.77 (dd, J = 7.1, 5.9 Hz, 2H), 3.73-3.69 (m, 2H), 3.68-3.62 (m, 24H), 3.57 (m, J = 21.0, 10.6, 4.8 Hz, 6H), 3.31 (t, J = 5.3 Hz, 2H), 3.26-3.20 (m, 1H), 3.00-2.85 (m, 9H), 2.57 (ddd, J = 15.2, 7.7, 5.1 Hz, 1H), 2.50 (td, J = 7.1, 5.3 Hz, 3H), 1.68-1.56 (m, 4H), 1.45-1.30 (m, 4H), 1.28-1.16 (m, 3H). ^{13}C NMR (150 MHz, D_2O) δ 174.07, 173.42, 173.24, 172.68, 172.43, 172.40, 162.99, 162.76, 143.95, 143.81, 142.91, 142.81, 134.29, 128.66, 128.47, 121.51, 121.31, 116.76, 116.61, 116.14, 116.07, 69.61, 69.51, 69.47, 69.40, 69.32, 69.24, 69.22, 68.57, 66.50, 55.45, 55.14, 53.30, 39.06, 38.97, 38.86, 38.38, 36.14, 35.56, 34.56, 34.34, 30.26, 26.16, 25.20, 23.94, 21.73. HRESI-MS m/z calcd for $C_{54}H_{86}N_8O_{19}$ $[M+2H]^{2+}/2$: 575.3005, found 575.3007.

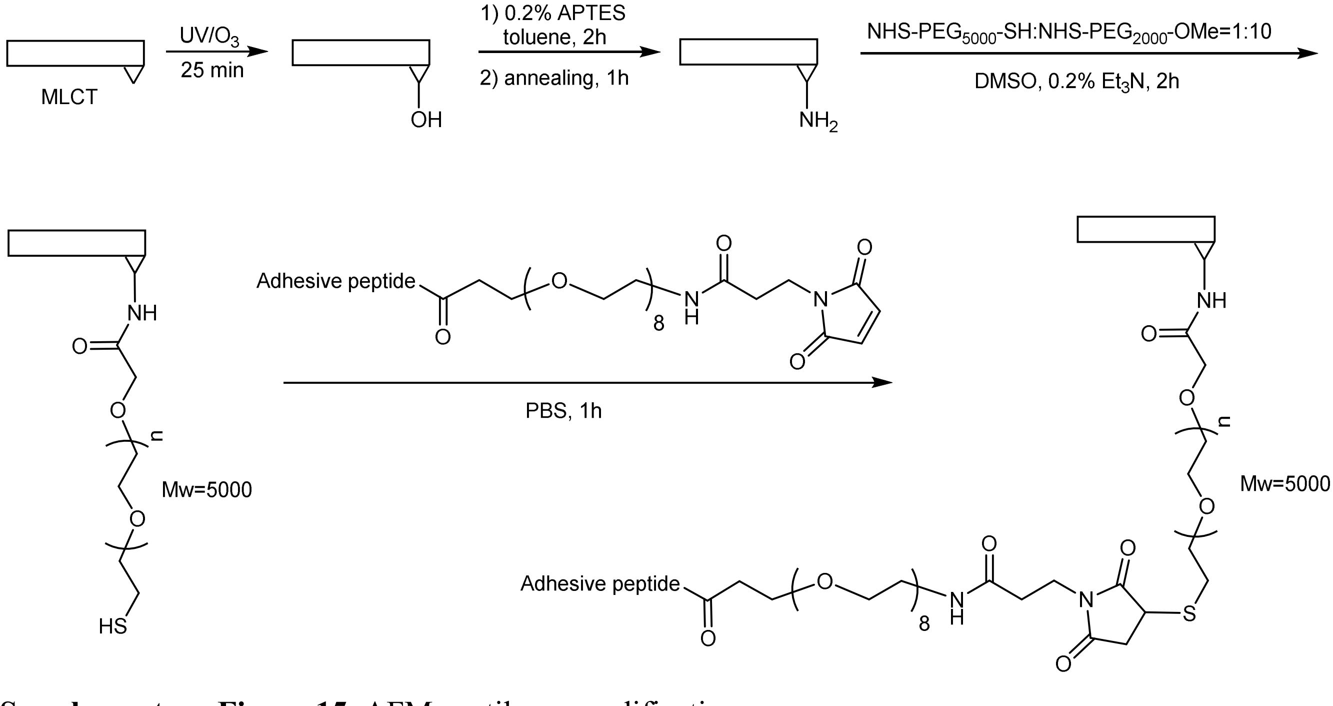

Supplementary Figure 15. AFM cantilever modification.

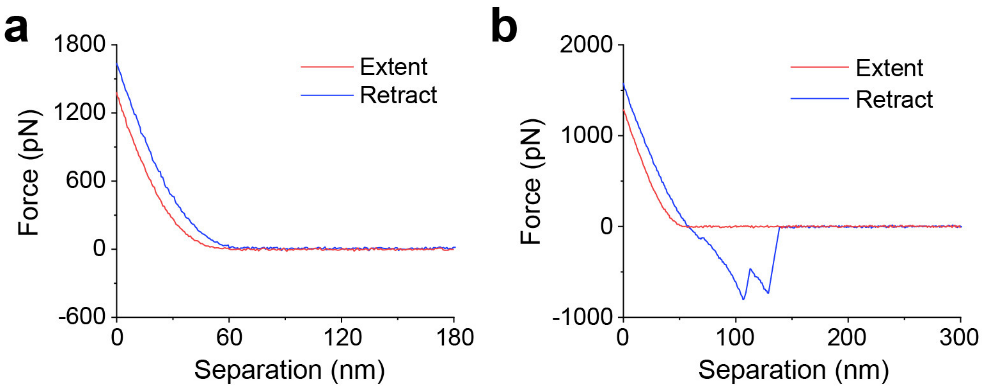

Supplementary Figure 16. Representative discarded data of F-D curves that the DbaYKY is not in contact with PEG hydrogel (**a**) and that multiple chains of DbaYKY interact with PEG hydrogel (**b**).

Solid-phase synthesis of OEG8-Mal conjugated peptides containing DOPA or lysine

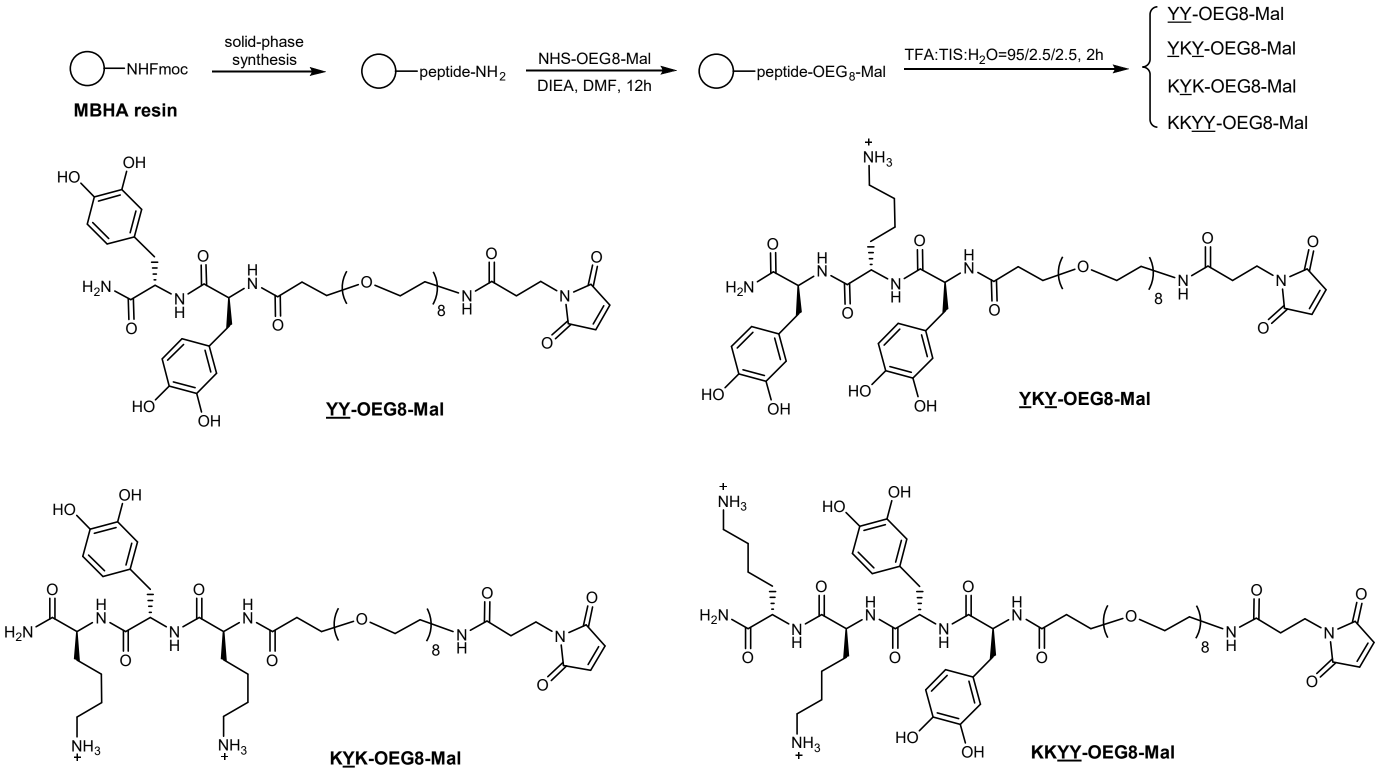

Supplementary Figure 17. Solid-phase synthesis of OEG8-Mal conjugated peptides containing DOPA or lysine.

The OEG8-Mal conjugated peptides were synthesized by solid phase synthesis on rink amide-MBHA resins (0.516 mmol/g). The resin (1 equiv) was swollen in CH_2Cl_2 (2 mL) for overnight. After draining the solvent, the resin was treated with 20% piperidine in DMF (2 mL) for 12 min and washed with DMF (3 × 5

mL) and CH_2Cl_2 (3 × 5 mL). The synthesis of YY, YKY, KYK and KKYY was conducted by following aforementioned protocol in protected HOOC-YKY-NHFmoc synthesis. After completion of the couplings reaction, the Fmoc group was removed. Then the conjugation with OEG8-Mal were continued for overnight in the presence of 2 mL of NHS-OEG8-Mal (2 equiv) and DIEA (6 equiv) in DMF. Cleavage of OEG8-Mal conjugated peptide was achieved by using 2 mL of a scavenging mixture of TFA/TIS/H_2O (95/2.5/2.5 v/v/v) for 2 h. The resin was filtered and rinsed with 1 mL of TFA. The filtrate containing the deprotected product was concentrated by a stream of N_2 to a small volume, and the product was precipitated out with cold methyl tert-butyl ether (15 mL) as a white solid. This precipitate was collected by centrifugation and dried under a N_2 flow. After another dissolution-precipitation cycle and drying under vacuum, the peptide was purified by preparative RP-HPLC (SHIMADZU LC-20AR, 00G-4252-P2-AX Luna 5u C18(2) column, 1 mL injection volume, 15 mL/min flow rate, MeCN:H_2O 1:4→3:2, with 0.1% TFA over 25 minutes).

For YY-OEG8-Mal: ^{1}H NMR (600 MHz, D_2O) δ 6.84-6.78 (m, 4H), 6.74 (dd, J = 18.3, 2.1 Hz, 2H), 6.64 (dd, J = 8.1, 2.1 Hz, 1H), 6.55 (dd, J = 8.1, 2.1 Hz, 1H), 4.48 (td, J = 7.7, 6.8, 5.2 Hz, 2H), 3.81-3.73 (m, 2H), 3.72-3.60 (m, 26H), 3.60-3.49 (m, 6H), 3.31 (t, J = 5.3 Hz, 2H), 3.03 (dd, J = 14.1, 5.6 Hz, 1H), 2.89-2.73 (m, 3H), 2.54-2.41 (m, 4H). ^{13}C NMR (150 MHz, D_2O) δ 175.21, 173.87, 173.41, 172.68, 172.41, 143.89, 143.81, 142.84, 142.81, 134.26, 128.89, 128.66, 121.48, 121.28, 116.68, 116.59, 116.07, 115.98, 69.48, 69.46, 69.23, 68.57, 68.18, 66.45, 55.06, 54.27, 38.86, 36.11, 36.10, 35.58, 34.55, 34.33. HRESI-MS m/z calcd for $C_{44}H_{64}N_5O_{18}$ $[M+H]^+$: 950.4246, found 950.4237.

For YKY-OEG8-Mal: ^{1}H NMR (600 MHz, D_2O) δ 6.85-6.80 (m, 4H), 6.78 (dd, J = 3.8, 2.1 Hz, 2H), 6.69 (dd, J = 8.1, 2.1 Hz, 1H), 6.63 (dd, J = 8.1, 2.1 Hz, 1H), 4.51 (td, J = 6.9, 6.1, 1.9 Hz, 2H), 4.17 (dd, J = 8.0, 6.3 Hz, 1H), 3.82-3.75 (m, 2H), 3.75-3.62 (m, 26H), 3.62-3.51 (m, 6H), 3.32 (t, J = 5.3 Hz, 2H), 3.07 (dd, J = 14.1, 6.0 Hz, 1H), 2.96-2.78 (m, 5H), 2.57 (ddd, J = 15.0, 7.6, 5.1 Hz, 1H), 2.53-2.45 (m, 3H), 1.65-1.53 (m, 4H), 1.25-1.08 (m, 2H). ^{13}C NMR (150 MHz, D_2O) δ 175.42, 174.07, 173.43, 173.35, 172.87, 172.43,

162.79, 143.92, 143.80, 142.90, 142.82, 134.29, 128.90, 128.50, 121.43, 121.33, 121.19, 121.04, 117.22, 116.66, 116.61, 116.07, 116.04, 115.29, 69.47, 69.24, 68.57, 67.66, 66.49, 54.98, 54.41, 53.55, 39.03, 38.86, 36.09, 35.89, 35.57, 34.56, 34.34, 30.13, 26.15, 21.66. HRESI-MS m/z calcd for $C_{50}H_{76}N_7O_9$ $[M+H]^+$: 1078.5196, found 1078.5199;

For KYK-OEG8-Mal: ^{1}H NMR (600 MHz, D_2O) δ 6.88-6.84 (m, 3H), 6.77 (d, J = 2.1 Hz, 1H), 6.71 (dd, J = 8.2, 2.1 Hz, 1H), 4.57 (dd, J = 8.2, 7.1 Hz, 1H), 4.26-4.16 (m, 2H), 3.83-3.74 (m, 4H), 3.74-3.63 (m, 27H), 3.56 (t, J = 5.4 Hz, 2H), 3.33 (t, J = 5.4 Hz, 2H), 3.18-3.07 (m, 1H), 3.05-2.90 (m, 6H), 2.66-2.50 (m, 4H), 2.44 (t, J = 7.3 Hz, 1H), 2.09-2.02 (m, 1H), 1.91-1.76 (m, 2H), 1.75-1.60 (m, 7H), 1.44 (d, J = 3.4 Hz, 3H), 1.41-1.20 (m, 4H). ^{13}C NMR (150 MHz, D_2O) δ 175.95, 174.17, 173.73, 173.46, 172.80, 172.46, 163.02, 162.78, 143.94, 142.88, 134.34, 128.51, 121.43, 119.16, 118.75, 117.23, 116.75, 116.16, 116.14, 115.29, 69.47, 69.24, 68.57, 66.45, 62.12, 54.72, 53.84, 53.26, 39.06, 39.04, 38.86, 36.85, 35.86, 35.46, 34.57, 34.35, 30.95, 30.15, 30.12, 27.57, 27.55, 26.20, 26.15, 25.80, 21.90, 21.87, 18.00. HRESI-MS m/z calcd for $C_{47}H_{79}N_8O_{17}$ $[M+H]^+$: 1027.5563, found 1027.5562;

For KKYY-OEG8-Mal: ^{1}H NMR (600 MHz, D_2O) δ ^{1}H NMR (600 MHz, Deuterium Oxide) δ 6.86-6.80 (m, 4H), 6.73 (dd, J = 21.3, 2.1 Hz, 2H), 6.61 (dt, J = 8.4, 2.4 Hz, 2H), 4.50 (t, J = 7.3 Hz, 2H), 4.25-4.19 (m, 2H), 3.81-3.74 (m, 2H), 3.73-3.62 (m, 26H), 3.61-3.51 (m, 6H), 3.32 (t, J = 5.4 Hz, 2H), 3.03-2.99 (m, 4H), 2.95-2.78 (m, 4H), 2.57-2.43 (m, 4H), 1.89-1.62 (m, 8H), 1.52-1.26 (m, 4H). ^{13}C NMR (150 MHz, D_2O) δ 176.26, 174.02, 173.44, 172.98, 172.73, 172.43, 172.16, 162.79, 143.89, 142.88, 142.86, 138.28, 134.29, 128.62, 128.32, 121.36, 121.25, 119.15, 117.22, 116.71, 116.56, 116.04, 116.02, 115.29, 69.51, 69.48, 69.46, 69.24, 68.57, 66.46, 55.03, 54.43, 53.30, 53.17, 39.08, 38.86, 36.11, 35.96, 35.61, 34.56, 34.34, 30.28, 30.20, 26.23, 26.19, 21.98, 21.83. HRESI-MS m/z calcd for $C_{56}H_{88}N_9O_{20}$ $[M+H]^+$: 1206.6146, found 1206.6149.

Synthesis of polymers without the adhesive short peptide DbaYKY

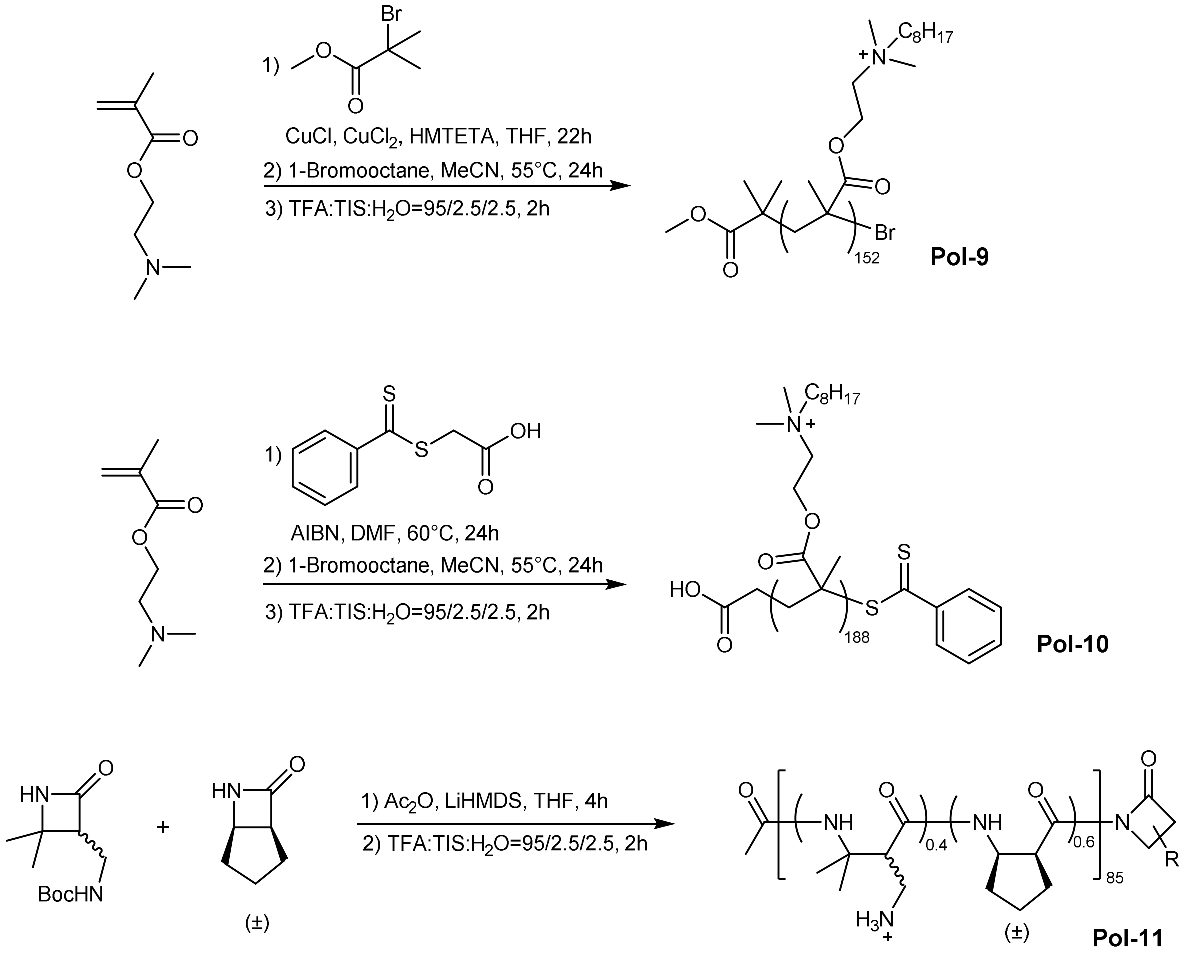

Supplementary Figure 18. Synthesis of Pol-9, Pol-10 and Pol-11.

The synthesis of Pol-9, Pol-10 and Pol-11 are conducted by following the protocols described above for Pol-1, Pol-3 and Pol-7, respectively. The side-chain protected polymer was characterized by GPC. Complete deprotection and purity of Pol-11 was confirmed by ^{1}H NMR.

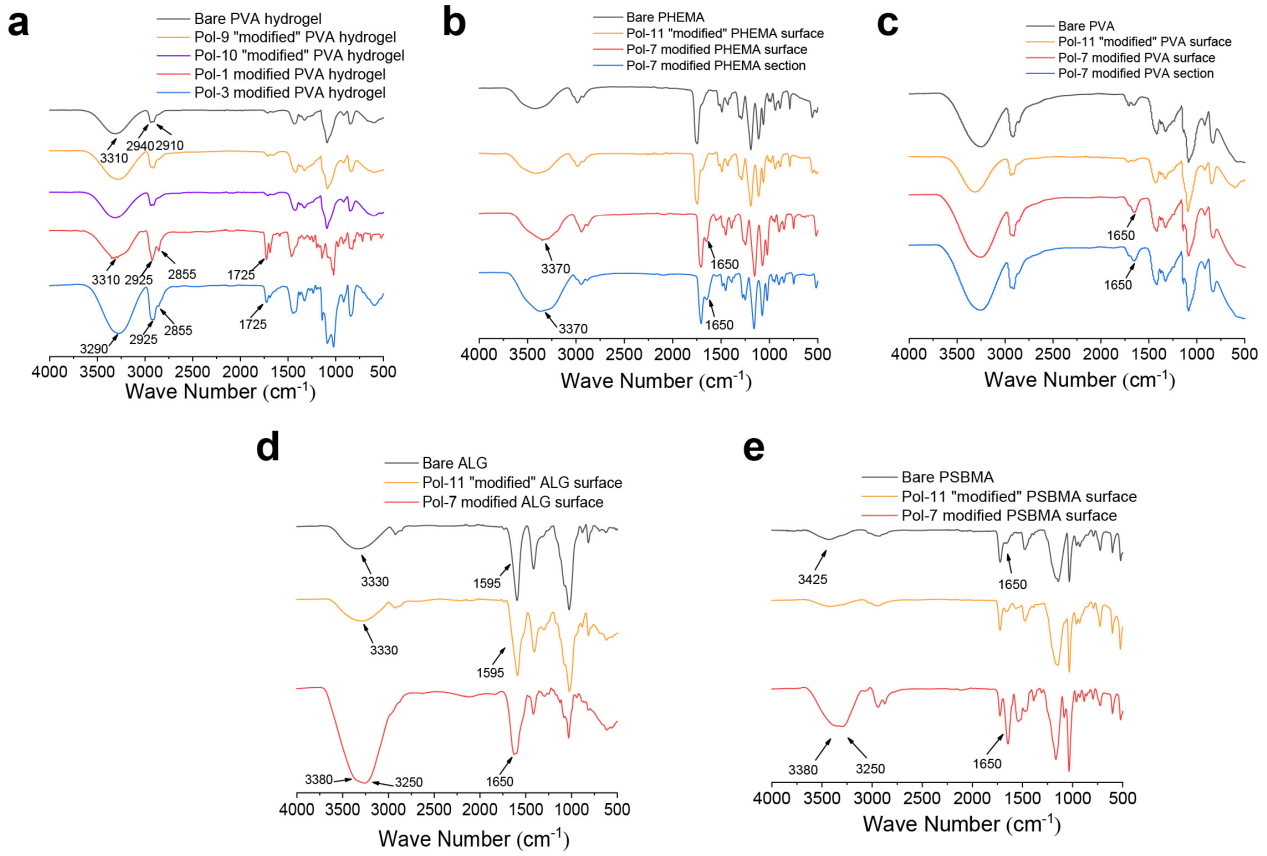

Supplementary Figure 19. ATR-FTIR characterization of bare and polymer-modified hydrogels.

Dry weight ratio of hydrogels

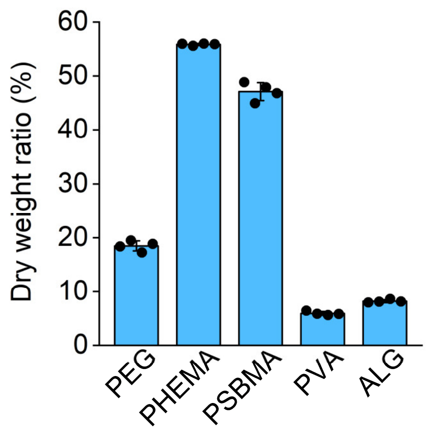

Supplementary Figure 20. Dry weight ratio of hydrogels. $n = 4$, mean values ± s.d.

Quantitative analysis using fluorescamine method

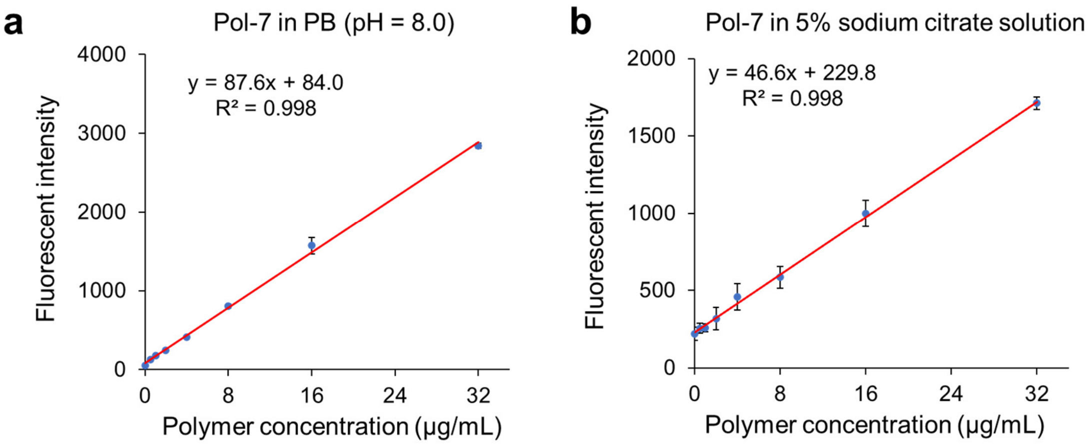

Supplementary Figure 21. Calibration curve for quantification of hydrogel modification by fluorescamine method. (a) Pol-7 in Phosphate buffer (PB) (pH = 8.0) for PVA calibration; (b) Pol-7 in 5% sodium citrate solution for ALG calibration.

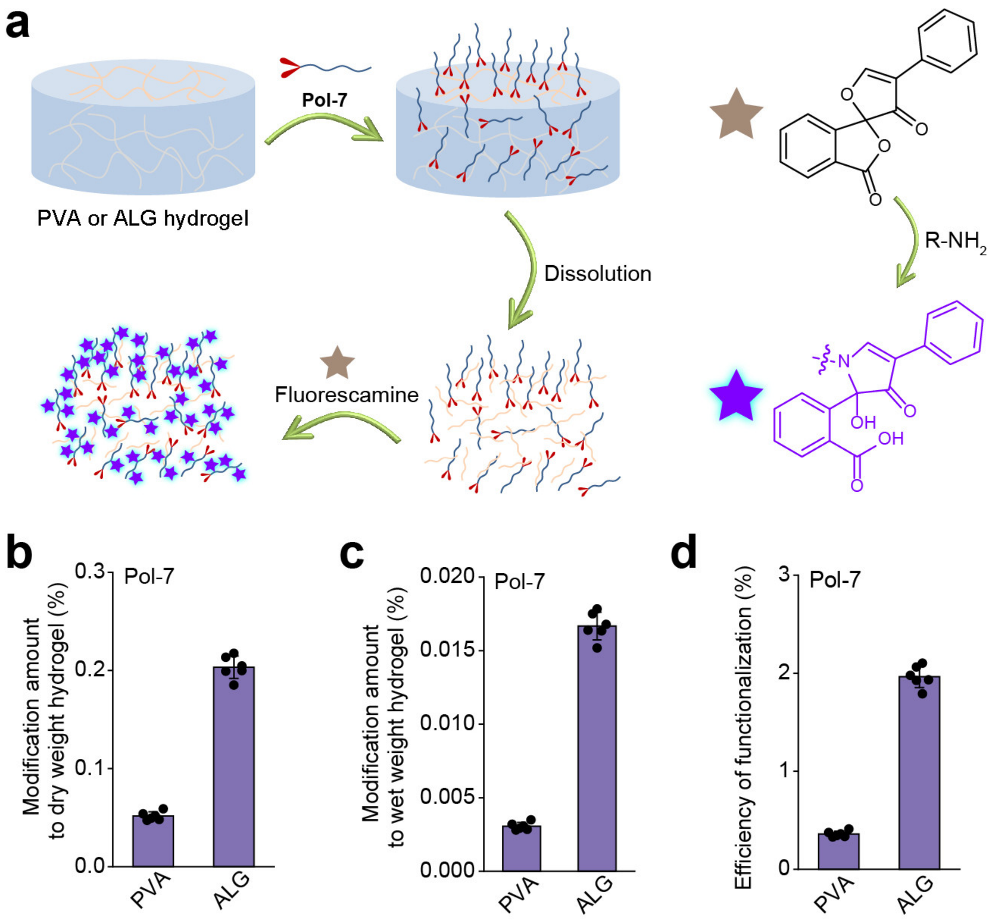

Supplementary Figure 22. Quantitative analysis of hydrogel modification by fluorescamine method. (**a**) A schematic representation for quantifying the modification amount of Pol-7 by fluorescamine method. Pol-7 modified PVA and ALG hydrogels were dissolved in Milli-Q water at 90 °C and 5% sodium citrate solution at room temperature, respectively, and then quantified by fluorescamine. (**b-d**) Quantitative analysis of Pol-

7 on PVA and ALG hydrogels, including modification amount to dry weight hydrogel (%) that is calculated by the formula: (weight of molecules bound to hydrogel/dry weight of initial hydrogel) × 100 (**b**), modification amount to wet weight hydrogel (%) that is calculated by the formula: (weight of molecules bound to hydrogel/wet weight of initial hydrogel) × 100 (**c**), and efficiency of functionalization (%) that is calculated by the formula: (peptide bound to hydrogel/binding peptide used initially) × 100 (**d**). $n = 6$, mean values ± s.d.

Synthesis of rhodamine conjugated DbaYKY

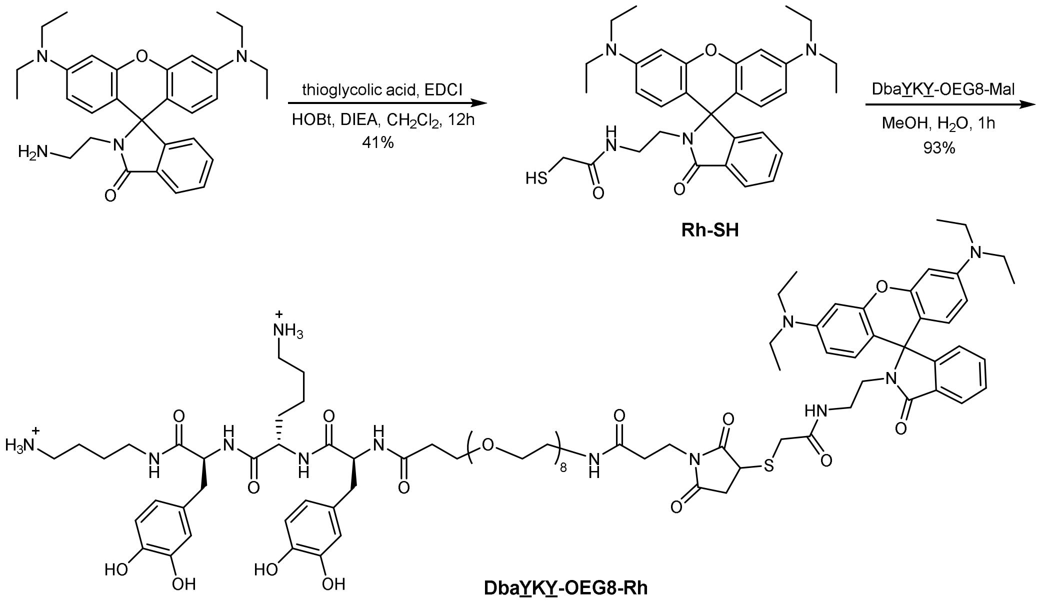

Supplementary Figure 23. Synthesis of Rhodamine conjugated DbaYKY.

Synthesis of Rh-SH. 2-(2-aminoethyl) rhodamine B amide (500 mg, 1.03 mmol) in CH_2Cl_2 (15 mL) was mixed with thioglycolic acid (358 μL, 5.16 mmol), followed by addition of EDCI (989 mg, 5.16 mmol), HOBt (697 mg, 5.16 mmol) and DIEA (853 μL, 5.16 mmol) to the reaction. The reaction mixture was stirred at room temperature for 12 h in a dark condition. The mixture was washed with H_2O (30 mL × 3), brine (10 mL), dried over $MgSO_4$ and concentrated. The crude product was purified by silica-gel flash chromatography using 50:1 CH_2Cl_2:MeOH as the eluent to obtain Rh-SH (spirolactam:xanthylium = 8:1) as a light purple solid (233 mg, 41% yield). ^1H NMR (spirolactam form; 400 MHz, CD_3OD) δ 7.94-7.90 (m, 1H), 7.61-7.55 (m, 2H), 7.10-7.07 (m, 1H), 6.92 (s, 2H), 6.78-6.67 (m, 2H), 6.64 (d, J = 8.8 Hz, 2H), 3.52 (q, J = 7.1 Hz, 8H), 3.25 (t, J = 6.7 Hz, 2H), 2.98 (s, 2H), 2.94 (t, J = 6.7 Hz, 2H), 1.17 (t, J = 7.1 Hz, 12H). ^{13}C NMR

(spirolactam form; 100 MHz, CD_3OD) δ 171.63, 169.51, 169.41, 169.25, 153.83, 153.40, 153.38, 148.99, 148.93, 132.81, 130.54, 130.49, 128.33, 128.24, 128.12, 123.75, 122.14, 108.19, 108.16, 104.59, 104.56, 97.65, 65.78, 65.72, 43.95, 39.31, 38.32, 26.86, 11.59, 11.52. HRESI-MS m/z calcd for $C_{32}H_{39}N_4O_3S$ $[M+H]^+$: 559.2743, found 559.2744.

Synthesis of Rhodamine conjugated DbaYKY (DbaYKY-OEG8-Rh). DbaYKY-OEG8-Mal (15 mg, 10.9 μmol) was added to a solution of Rh-SH (7.3 mg, 13.1 μmol) in MeOH (0.5 mL). The mixture was stirred at room temperature for 1 h, and filtered through 0.22 μm nylon membrane. The product was purified by preparative RP-HPLC (SHIMADZU LC-20AR, 00G-4252-P2-AX Luna 5u C18(2) column, 1 mL injection volume, 13 mL/min flow rate, MeCN:H_2O 1:4→19:1, with 0.1% TFA over 30 minutes) to give DbaYKY-OEG8-Rh as a light pink solid (19.8 mg, 93% yield). ^1H NMR (600 MHz, D_2O) δ 8.01-7.96 (m, 1H), 7.69 (tt, J = 7.4, 5.9 Hz, 2H), 7.62 (dd, J = 3.5, 2.3 Hz, 2H), 7.24-7.18 (m, 3H), 7.05 (ddd, J = 8.8, 6.9, 2.1 Hz, 2H), 6.82 (dd, J = 19.8, 8.1 Hz, 2H), 6.75 (t, J = 2.3 Hz, 2H), 6.68 (dd, J = 8.2, 2.1 Hz, 1H), 6.63 (dd, J = 8.2, 2.1 Hz, 1H), 4.50 (t, J = 7.6 Hz, 1H), 4.34 (dd, J = 8.7, 7.4 Hz, 1H), 4.19 (dd, J = 8.4, 6.1 Hz, 1H), 3.94 (dd, J = 9.1, 4.1 Hz, 1H), 3.74-3.60 (m, 38H), 3.56 (m, 6H), 3.43 (d, J = 15.6 Hz, 1H), 3.37 (td, J = 6.9, 3.6 Hz, 2H), 3.33-3.26 (m, 3H), 3.25-3.17 (m, 2H), 2.99-2.81 (m, 11H), 2.64 (dd, J = 18.9, 4.1 Hz, 1H), 2.57 (ddd, J = 15.2, 7.7, 5.2 Hz, 1H), 2.52-2.42 (m, 3H), 1.68-1.55 (m, 4H), 1.44-1.30 (m, 4H), 1.28-1.17 (m, 2H), 1.14 (t, J = 7.2 Hz, 13H). ^{13}C NMR (150 MHz, D_2O) δ 177.80, 177.19, 174.04, 173.19, 172.90, 172.66, 172.37, 171.19, 170.67, 166.69, 163.19, 162.96, 162.72, 162.48, 151.98, 151.88, 151.76, 143.95, 143.81, 142.92, 142.81, 138.41, 134.43, 130.80, 129.99, 128.94, 128.90, 128.63, 128.45, 123.87, 123.84, 123.19, 121.49, 121.29, 120.13, 120.10, 119.13, 118.17, 118.14, 118.09, 117.20, 116.75, 116.60, 116.51, 116.12, 116.04, 115.97, 115.27, 114.47, 114.19, 114.07, 113.33, 112.00, 111.59, 106.36, 69.46, 69.26, 68.56, 68.52, 66.49, 64.29, 55.45, 55.12, 55.05, 53.73, 53.26, 40.15, 39.05, 38.96, 38.89, 38.84, 38.76, 38.37, 38.13, 36.17, 36.14,

35.68, 35.57, 35.25, 35.20, 34.14, 34.10, 33.35, 30.26, 30.22, 26.42, 26.15, 25.19, 23.92, 21.73, 21.18, 9.58.

HRESI-MS m/z calcd for $C_{86}H_{124}N_{12}O_{22}S$ $[M+2H]^{2+}/2$: 854.4337, found 854.4258.

Quantitative analysis using rhodamine method

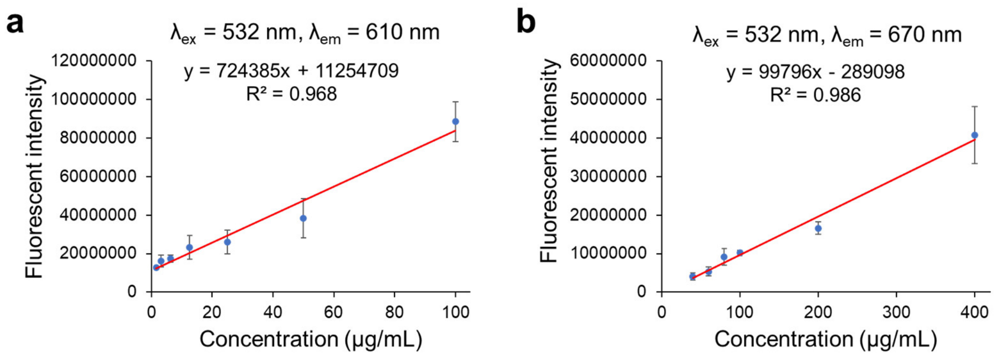

Supplementary Figure 24. Calibration curve for quantification of hydrogel modification by rhodamine method. (a) λ_{ex} = 532 nm, λ_{em} = 610 nm; (b) λ_{ex} = 532 nm, λ_{em} = 670 nm

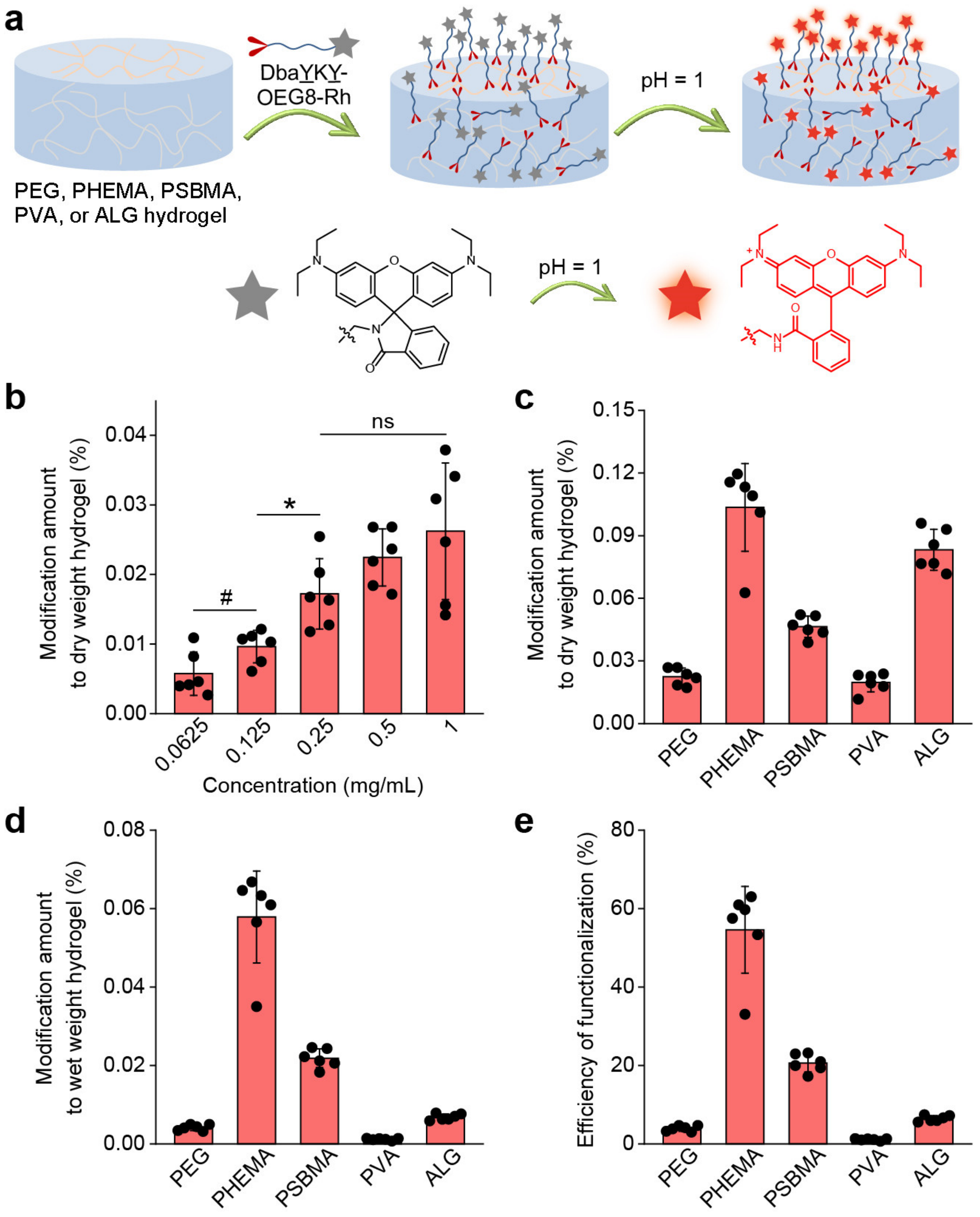

Supplementary Figure 25. Quantitative analysis of hydrogel modification by rhodamine method. (**a**) A schematic representation for quantifying the modification amount of DbaYKY-OEG8-Rh by rhodamine method. (**b**) Quantitative analysis of DbaYKY-OEG8-Rh in different concentrations (0.0625-1 mg/mL) on PEG hydrogels. (**c-e**) Quantitative analysis of DbaYKY-OEG8-Rh (0.5 mg/mL) on various hydrogels, including modification amount to dry weight hydrogel (%) that is calculated by the formula: (weight of molecules bound to hydrogel/dry weight of initial hydrogel) × 100 (**c**), modification amount to wet weight hydrogel (%) that is calculated by the formula: (weight of molecules bound to hydrogel/wet weight of initial hydrogel) × 100 (**d**), and efficiency of functionalization (%) that is calculated by the formula: (peptide bound to hydrogel/binding peptide used initially) × 100 (**e**). $n = 6$, mean values ± s.d. Statistical analysis: two-tailed t-test. $^{\#}P < 0.05$, $^{*}P < 0.01$. ns: not significant.

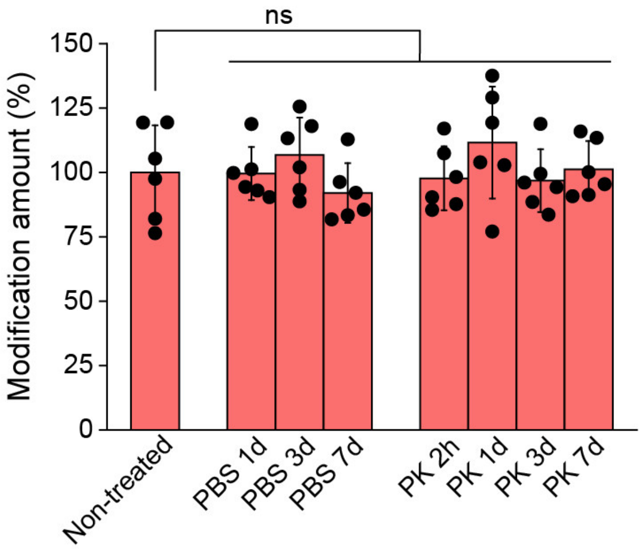

Supplementary Figure 26. Stability of DbaYKY-OEG8-Rh modified PEG hydrogels in PBS under staking for 1 day, 3 days and 7 days or in protease K (PK) solution at 37 °C for 2 h, 1 day, 3 days and 7 days. $n = 6$, mean values ± s.d. Statistical analysis: two-tailed t-test. ns: not significant.

Synthesis of DbaYKY-Ac

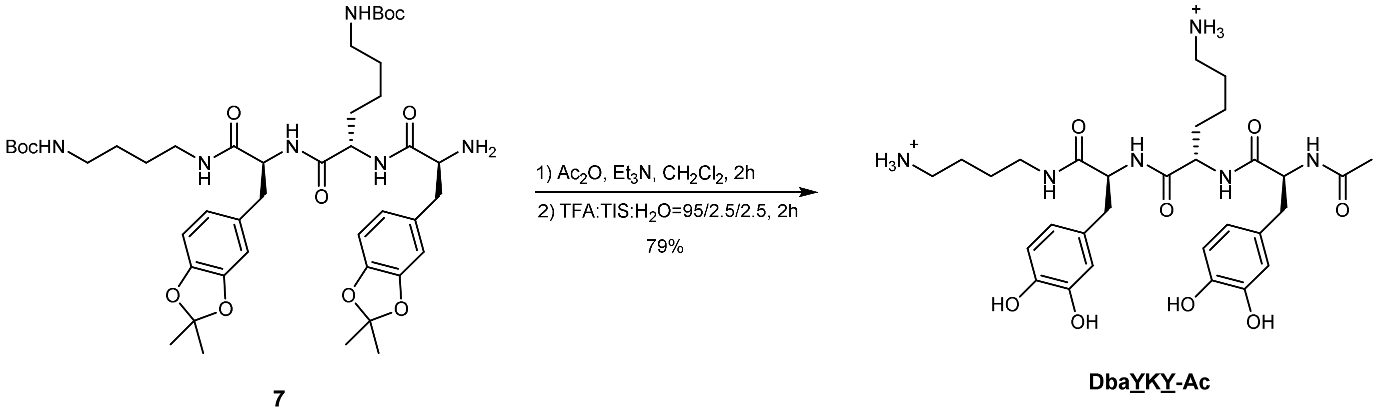

Supplementary Figure 27. Synthesis of DbaYKY-Ac.

Compound **7** (40 mg, 46.8 μmol) was dissolved in CH_2Cl_2 (0.4 mL), followed by addition of Ac_2O (8.8 μL, 93.6 μmol) and Et_3N (13 μL, 93.6 μmol) to the reaction. After the mixture was stirred at room temperature for 2 h, the reaction mixture was diluted with CH_2Cl_2 (30 mL), washed with saturated $NaHCO_3$ (30 mL), dried over $MgSO_4$ and concentrated to minimum amount. The crude protected product was purified by silica-gel flash chromatography using 20:1 CH_2Cl_2:MeOH as the eluent. The protecting groups were removed by

following aforementioned protocol in DbaYKY-OEG8-Mal synthesis to give the final product of DbaYKY-Ac as a light grey solid in a form of TFA salt (31 mg, 79% yield). ^{1}H NMR (600 MHz, D_2O) δ 6.85 (dd, J = 22.2, 8.1 Hz, 2H), 6.76 (dd, J = 5.4, 2.1 Hz, 2H), 6.71 (dd, J = 8.1, 2.1 Hz, 1H), 6.64 (dd, J = 8.1, 2.1 Hz, 1H), 4.43 (t, J = 7.7 Hz, 1H), 4.32 (dd, J = 8.8, 7.5 Hz, 1H), 4.16 (dd, J = 8.3, 6.0 Hz, 1H), 3.23 (dt, J = 12.4, 6.2 Hz, 1H), 3.00-2.81 (m, 9H), 1.99 (s, 3H), 1.63-1.53 (m, 4H), 1.43-1.31 (m, 4H), 1.25-1.13 (m, 2H). ^{13}C NMR (150 MHz, D_2O) 175.45, 174.09, 173.40, 172.55, 172.43, 163.03, 162.79, 162.55, 143.92, 143.79, 142.85, 142.79, 128.71, 128.52, 121.54, 121.30, 119.15, 117.22, 116.78, 116.64, 116.17, 116.12, 115.28, 107.54, 103.91, 55.48, 53.18, 39.04, 38.96, 38.37, 36.06, 36.02, 30.36, 26.13, 25.17, 23.92, 21.64, 21.47. HRESI-MS m/z calcd for $C_{30}H_{44}N_6NaO_8$ $[M+Na]^+$: 639.3118, found 639.3152.

Analysis of possible adhesion molecule transformation

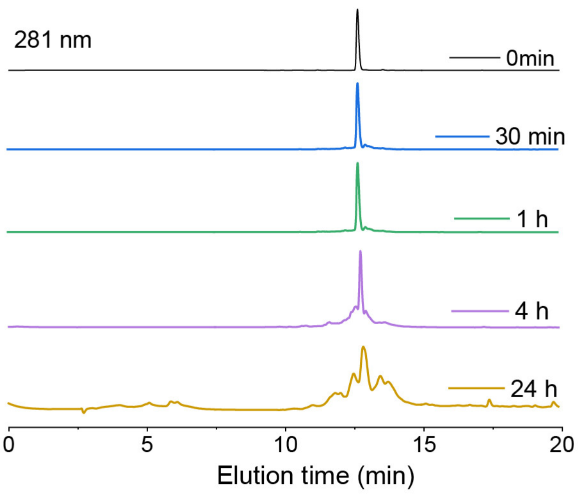

Supplementary Figure 28. HPLC trace of DbaYKY-Ac in Tris buffer (100 mM, pH 8.5) at different time.

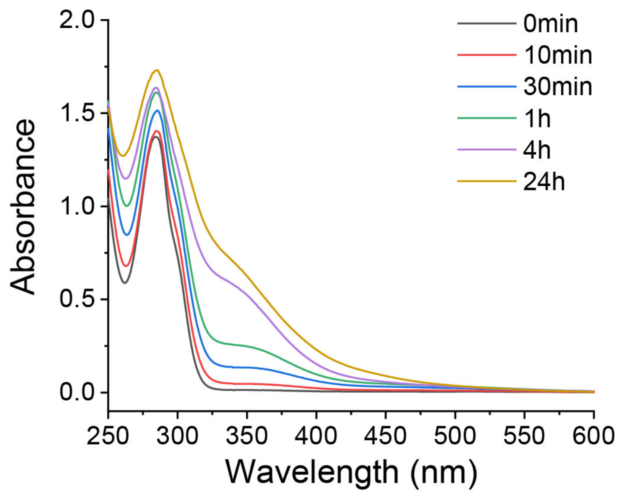

Supplementary Figure 29. UV-vis spectra of Dba<u>Y</u>K<u>Y</u>-Ac in Tris buffer (100 mM, pH 8.5) at different time.

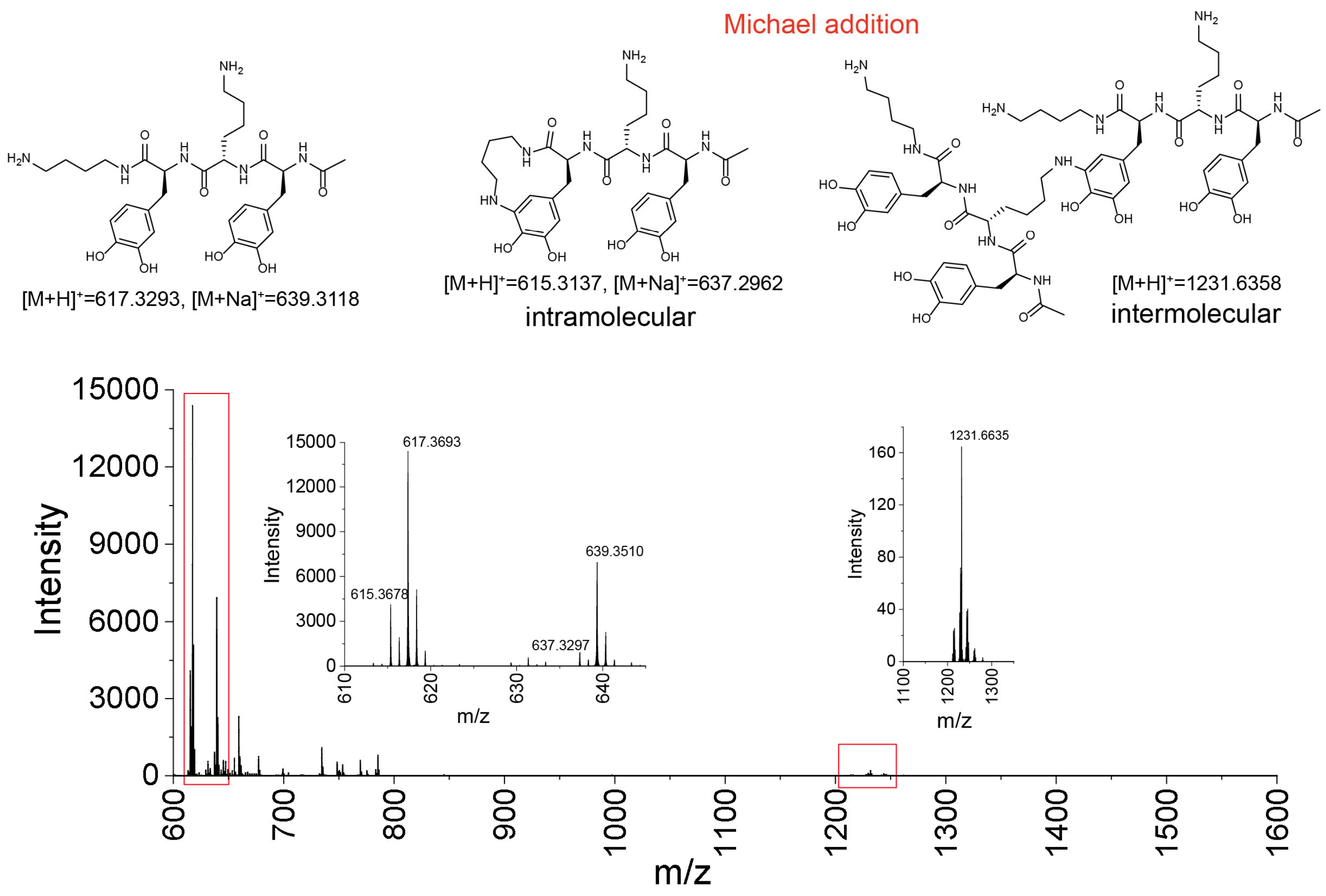

Supplementary Figure 30. MALDI-TOF-MS spectrum of Dba<u>Y</u>K<u>Y</u>-Ac after dissolution in Tris buffer (100 mM, pH 8.5) for 24 h.

Kaiser test of bare hydrogels and Pol-7 modified hydrogels

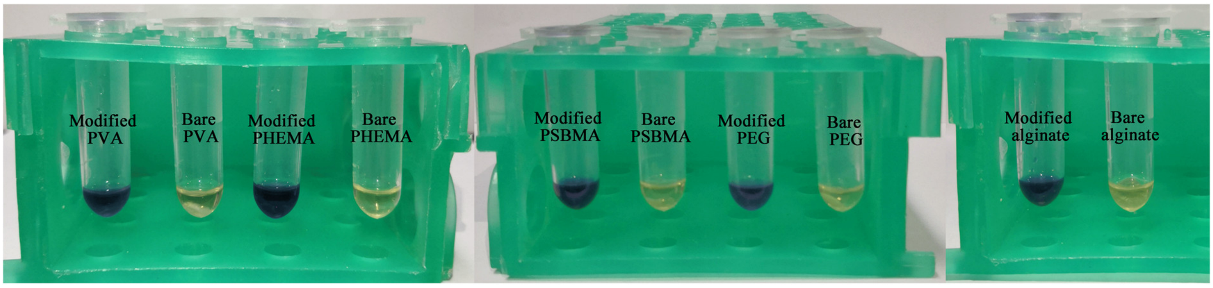

Supplementary Figure 31. The Kaiser test of bare hydrogels and Pol-7 modified hydrogels (PVA, PHEMA, PSBMA, PEG and alginate hydrogels respectively).

Fluorescent microscopy images of cell adhesion to the TCPS surface

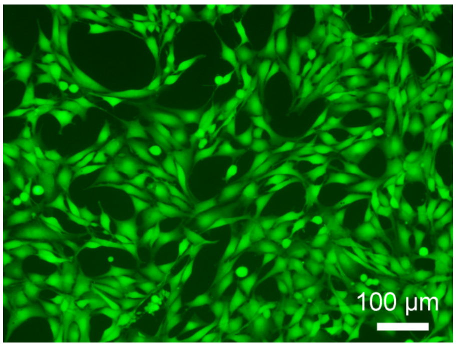

Supplementary Figure 32. Fluorescent microscopy images of 3T3 fibroblast cell adhesion to the TCPS surface. After incubation for 24 hours, cells were stained by LIVE/DEAD kit, calcein AM (green) for live cells.

Permeability test of polymers into hydrogels

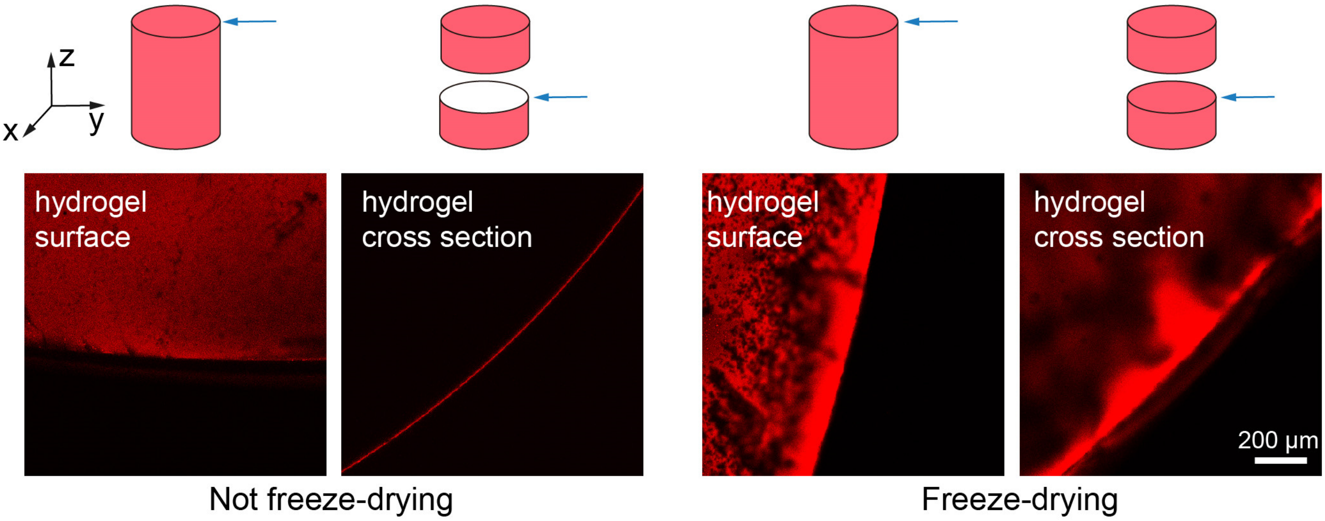

Supplementary Figure 33. Confocal images of surface and z-axis cross section of Dba<u>Y</u>K<u>Y</u>-OEG8-Rh functionalized PHEMA hydrogels that are processed with or without the freeze-drying step.

NMR spectra of compounds and polymers

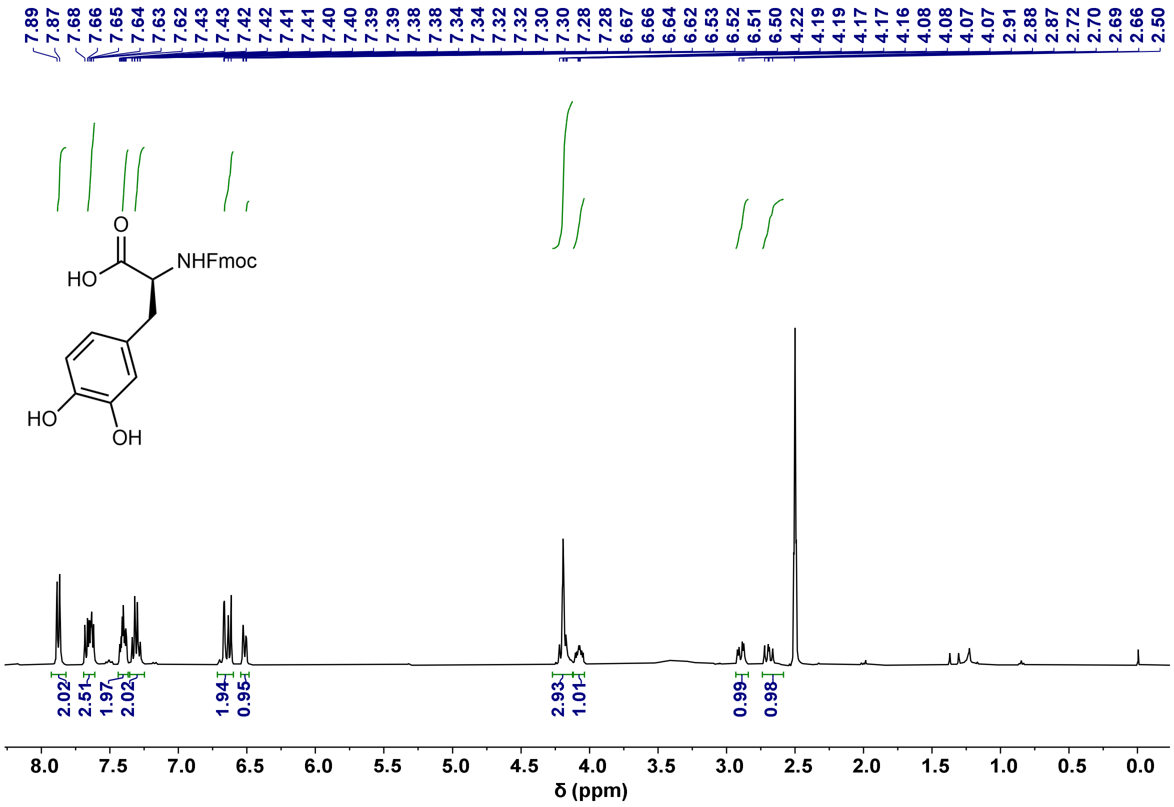

Supplementary Figure 34. ^{1}H NMR spectrum for in coumpound **1** in DMSO-*d*6, 400 MHz.

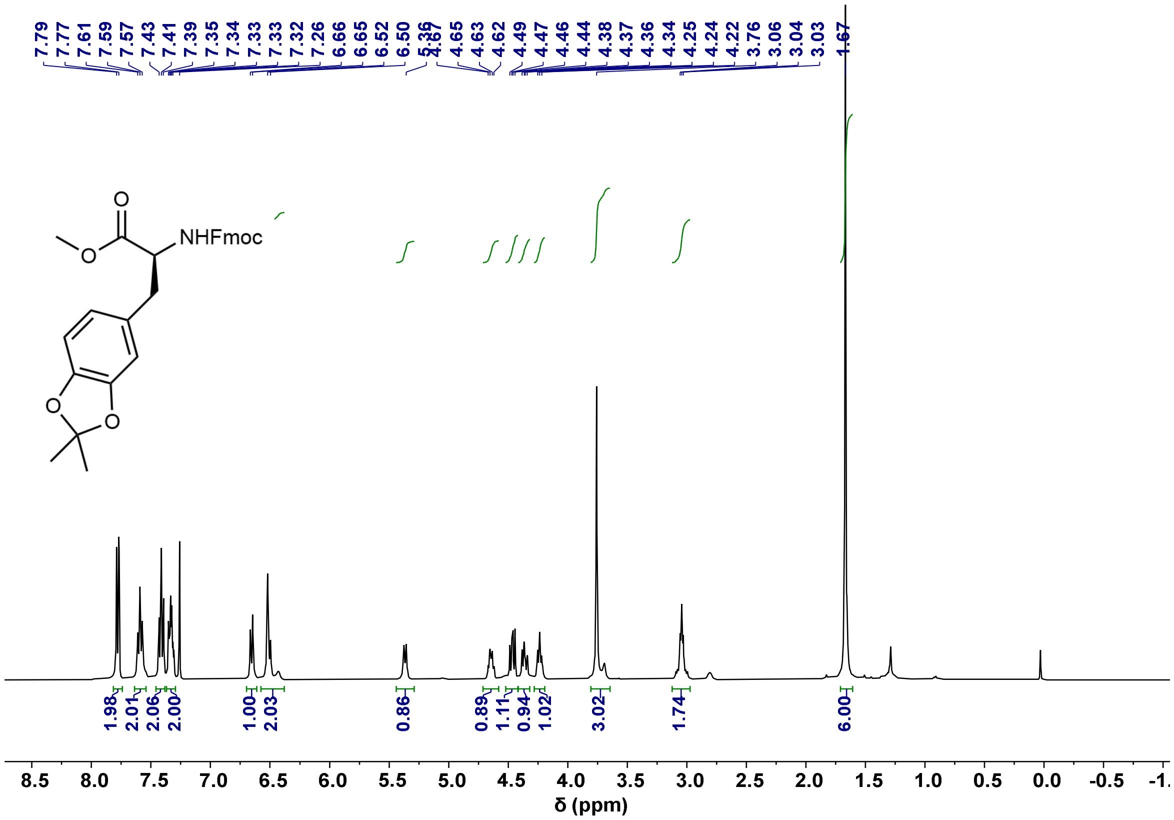

Supplementary Figure 35. ^{1}H NMR spectrum for in coumpound **2** in $CDCl_3$, 400 MHz.

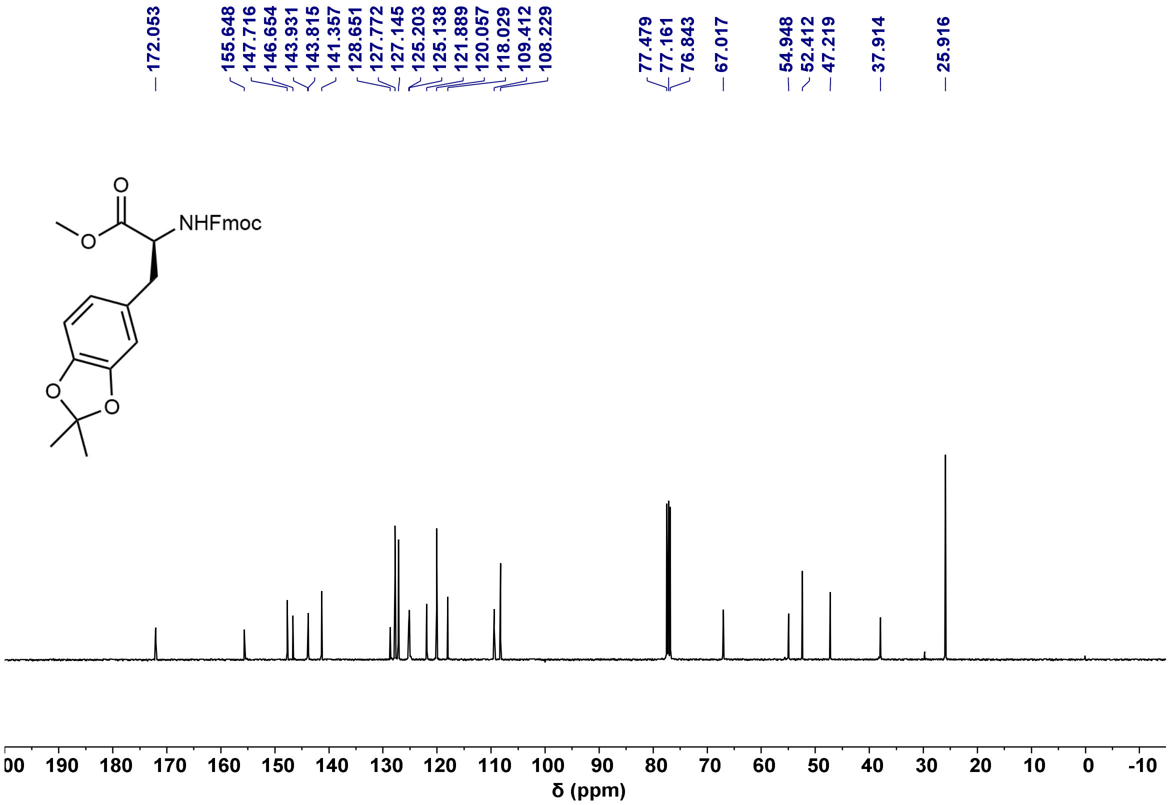

Supplementary Figure 36. ^{13}C NMR spectrum of compound **2** in $CDCl_3$, 100MHz.

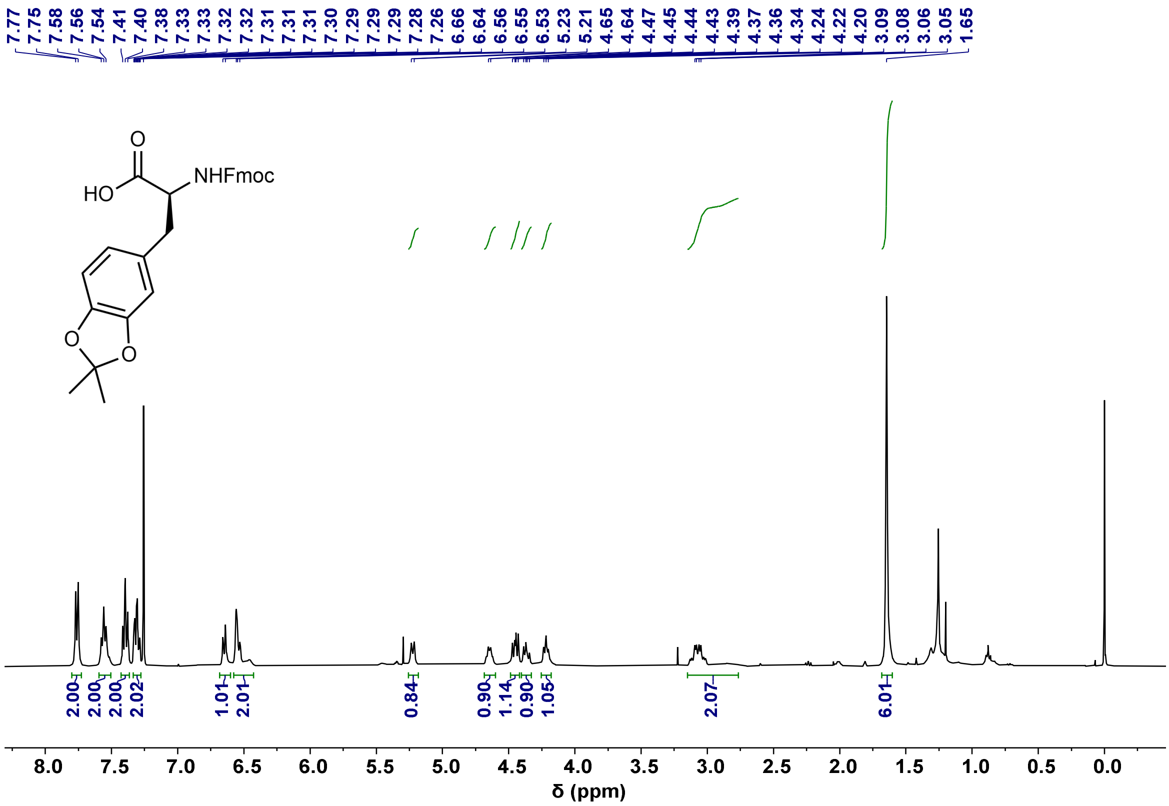

Supplementary Figure 37. ^{1}H NMR spectrum for in coumpound **3** in $CDCl_3$, 400 MHz.

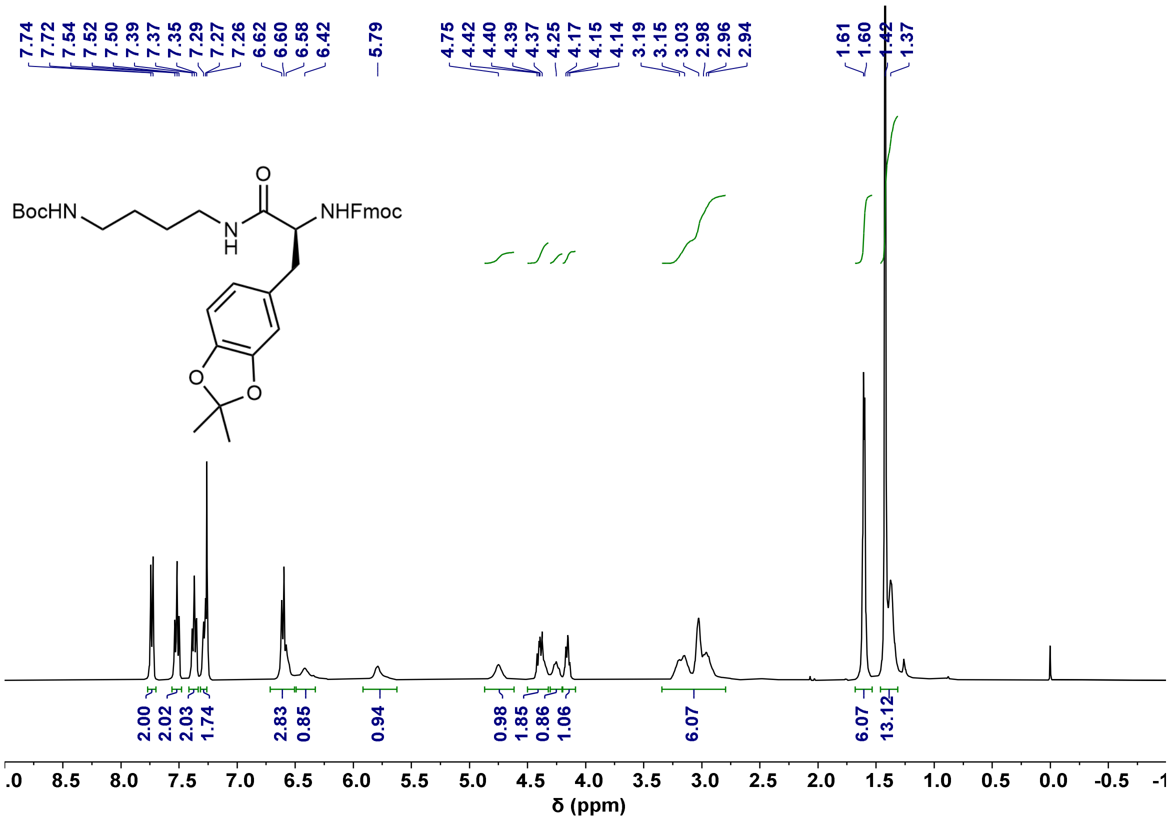

Supplementary Figure 38. ^{1}H NMR spectrum of compound **4** in $CDCl_3$, 400 MHz.

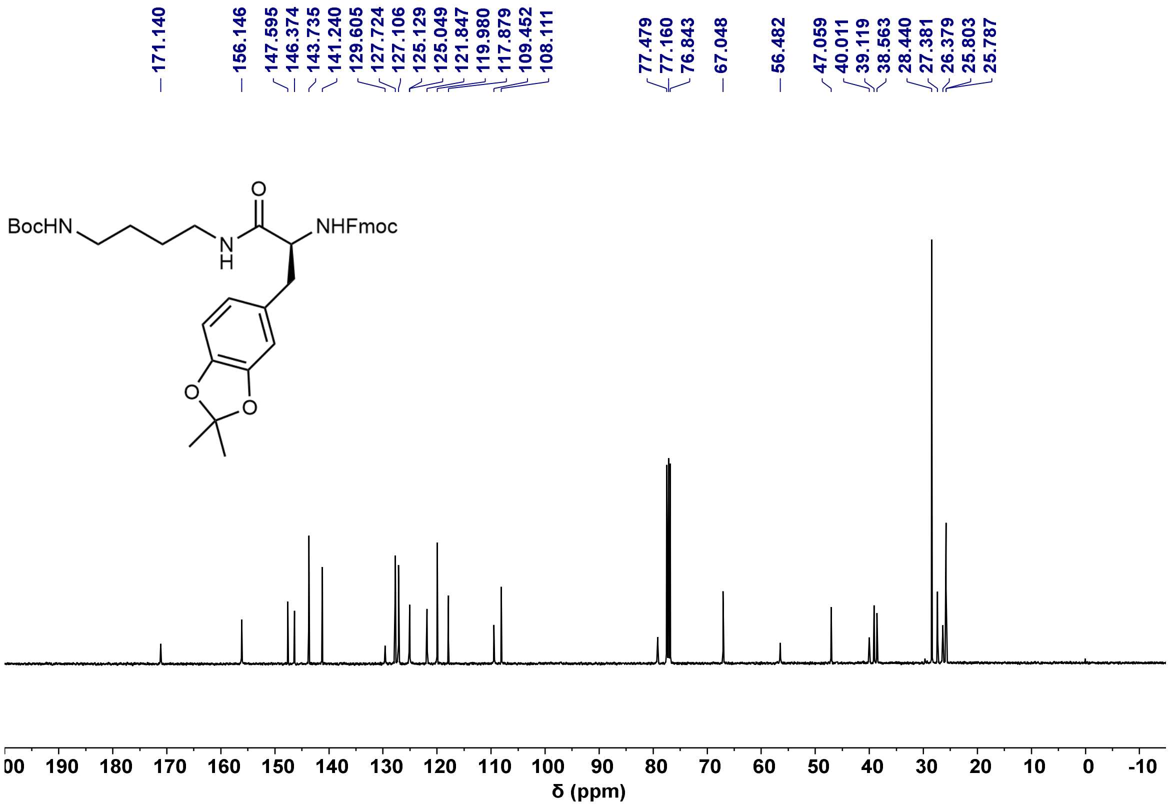

Supplementary Figure 39. ^{13}C NMR spectrum of compound **4** in $CDCl_3$, 100MHz.

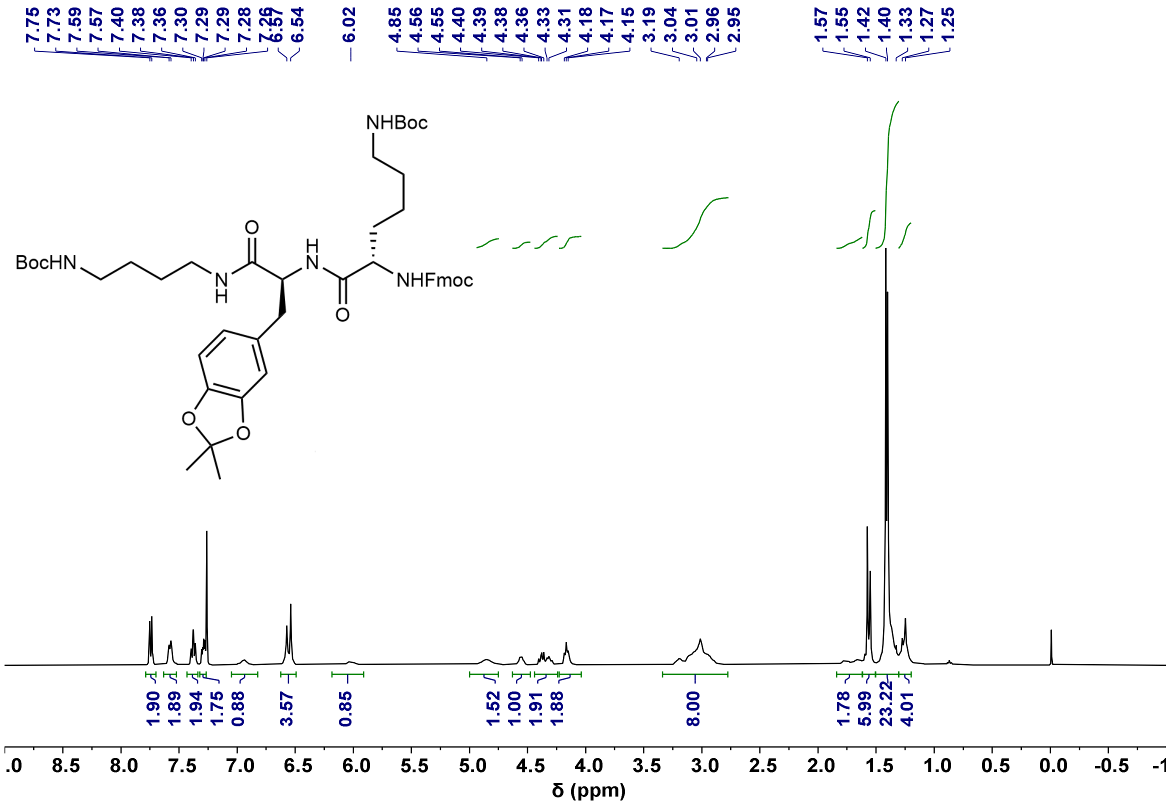

Supplementary Figure 40. ^{1}H NMR spectrum of compound **5** in $CDCl_3$, 400 MHz.

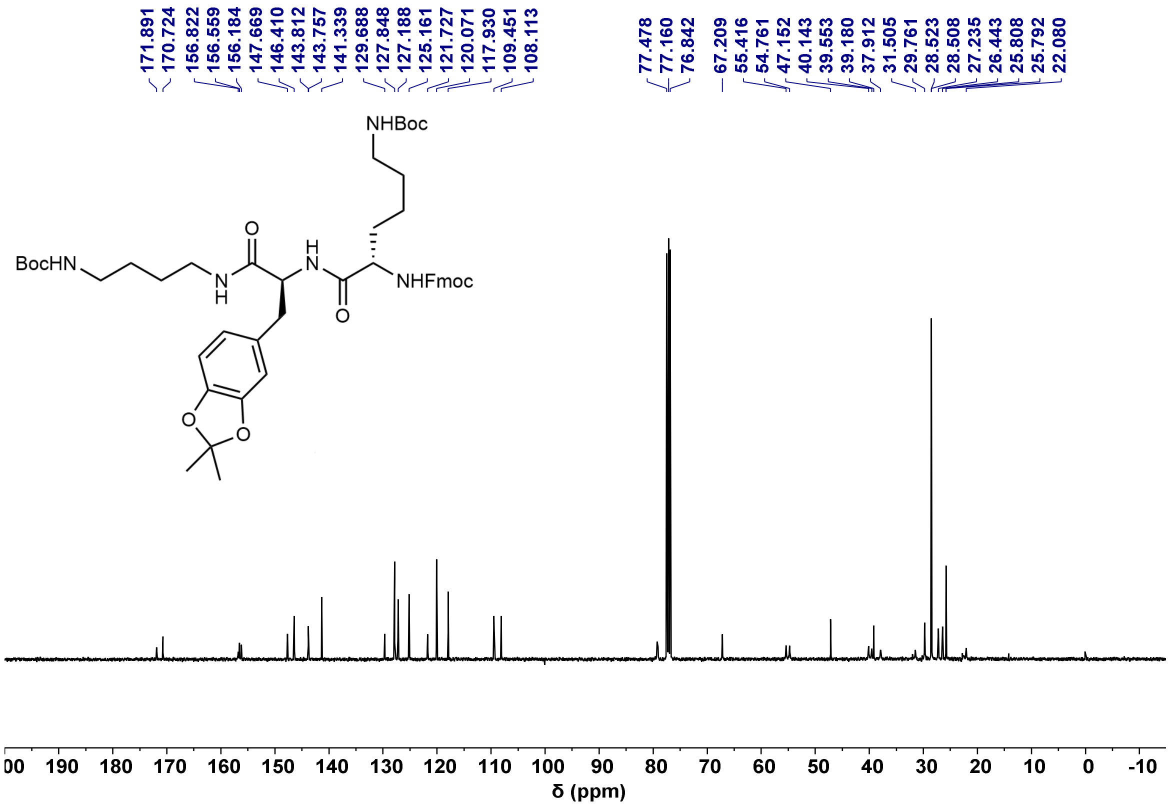

Supplementary Figure 41. ^{13}C NMR spectrum of compound **5** in $CDCl_3$, 100MHz.

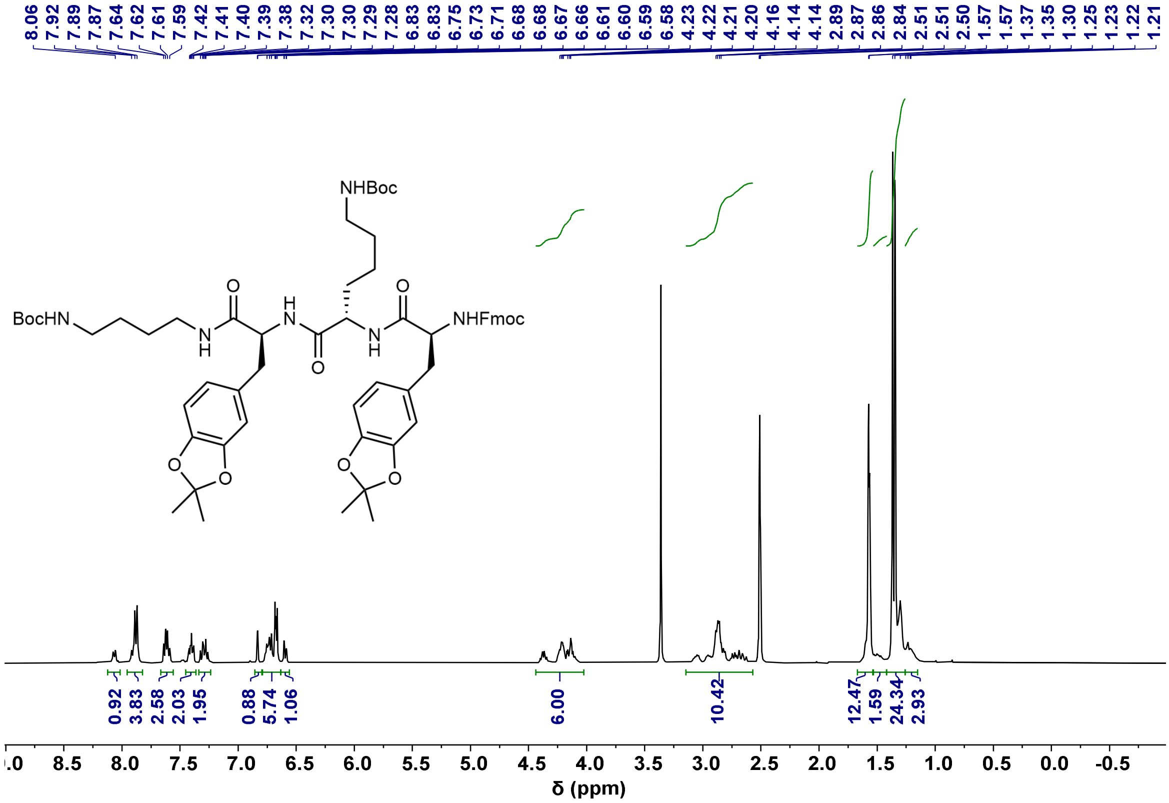

Supplementary Figure 42. ^{1}H NMR spectrum of compound **6** in DMSO-*d*6, 400 MHz.

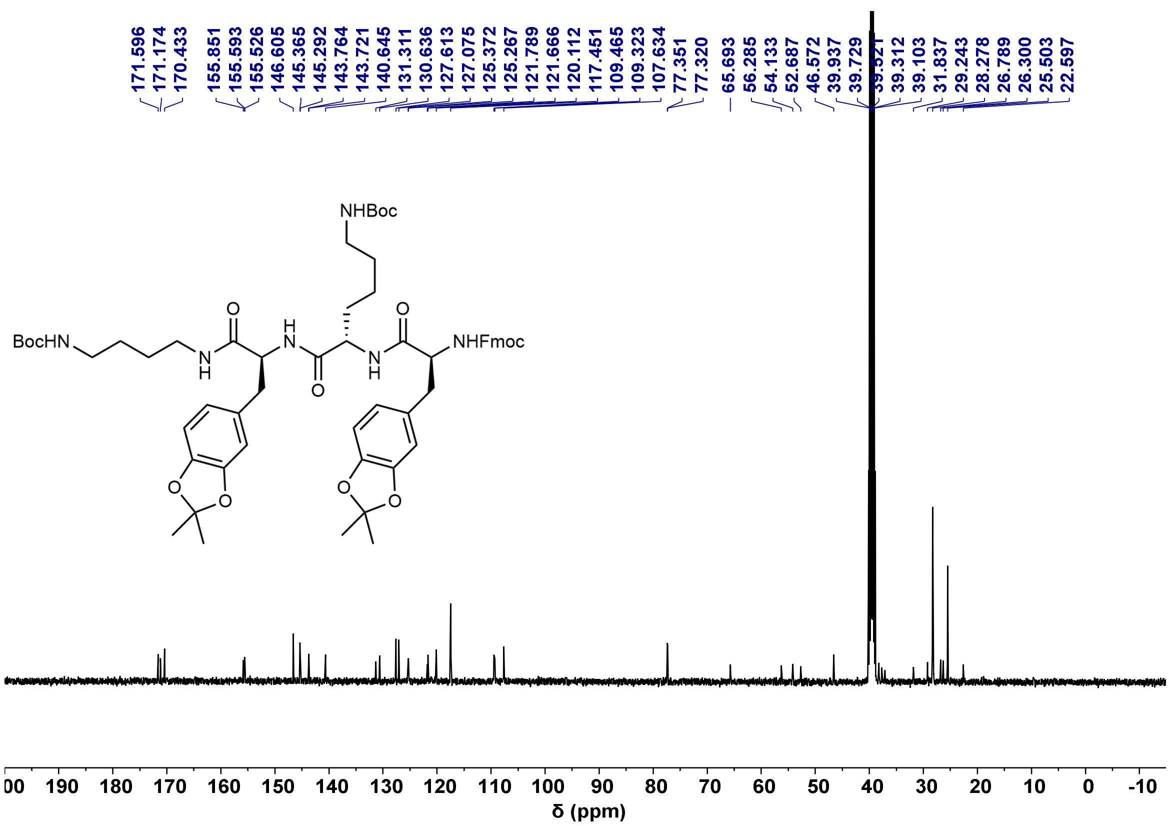

Supplementary Figure 43. ^{13}C NMR spectrum of compound **6** in DMSO-*d*6, 100MHz.

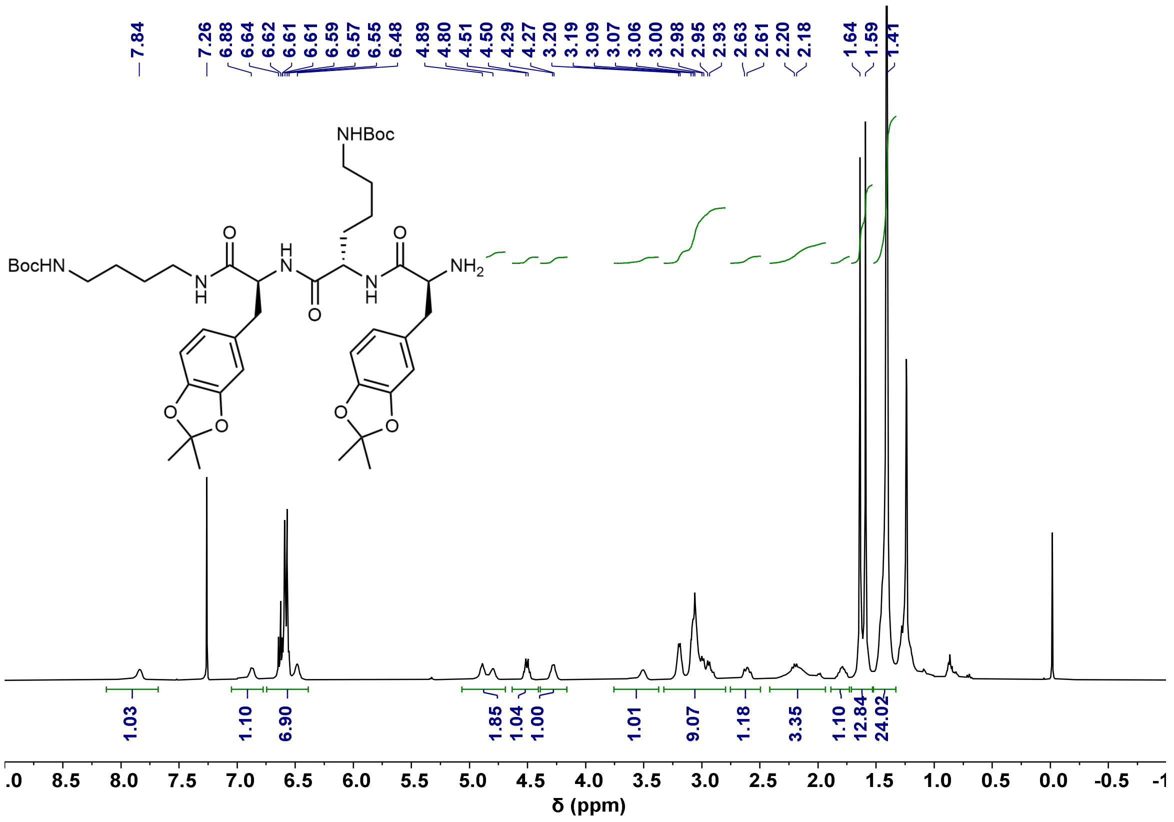

Supplementary Figure 44. ^{1}H NMR spectrum of compound **7** in $CDCl_3$, 400 MHz.

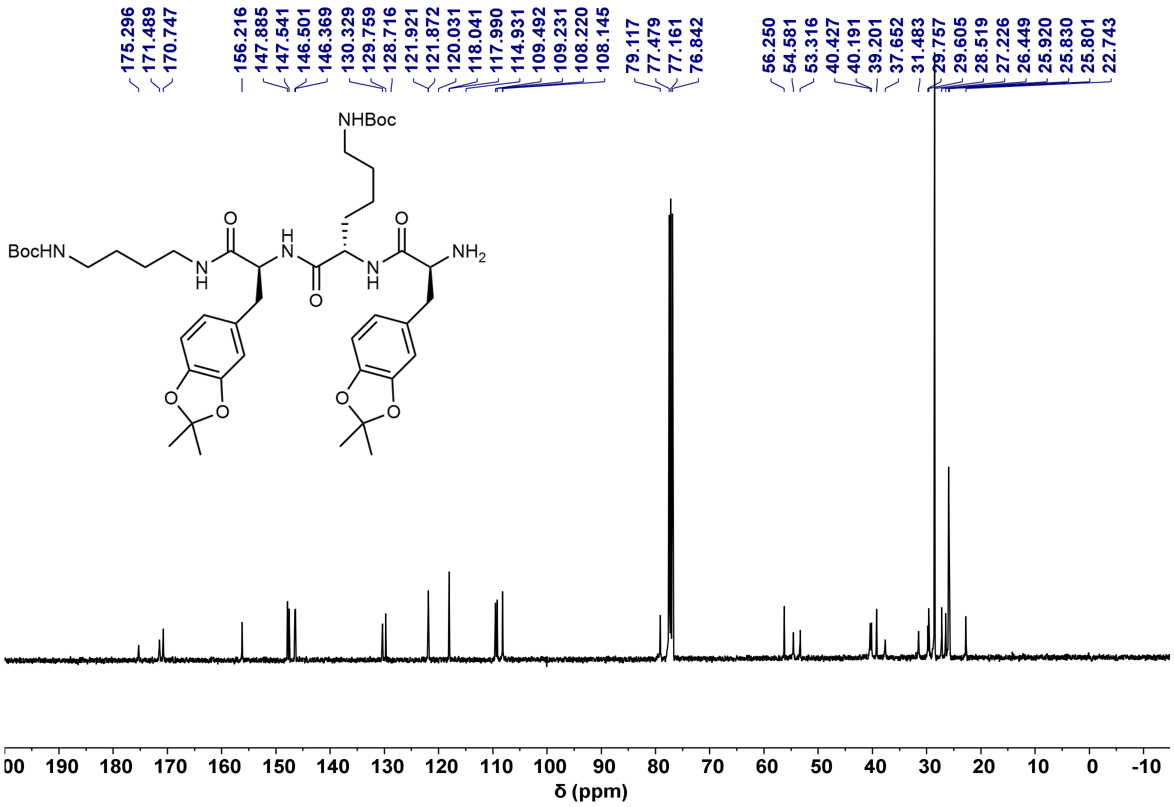

Supplementary Figure 45. ^{13}C NMR spectrum of compound **7** in $CDCl_3$, 100MHz.

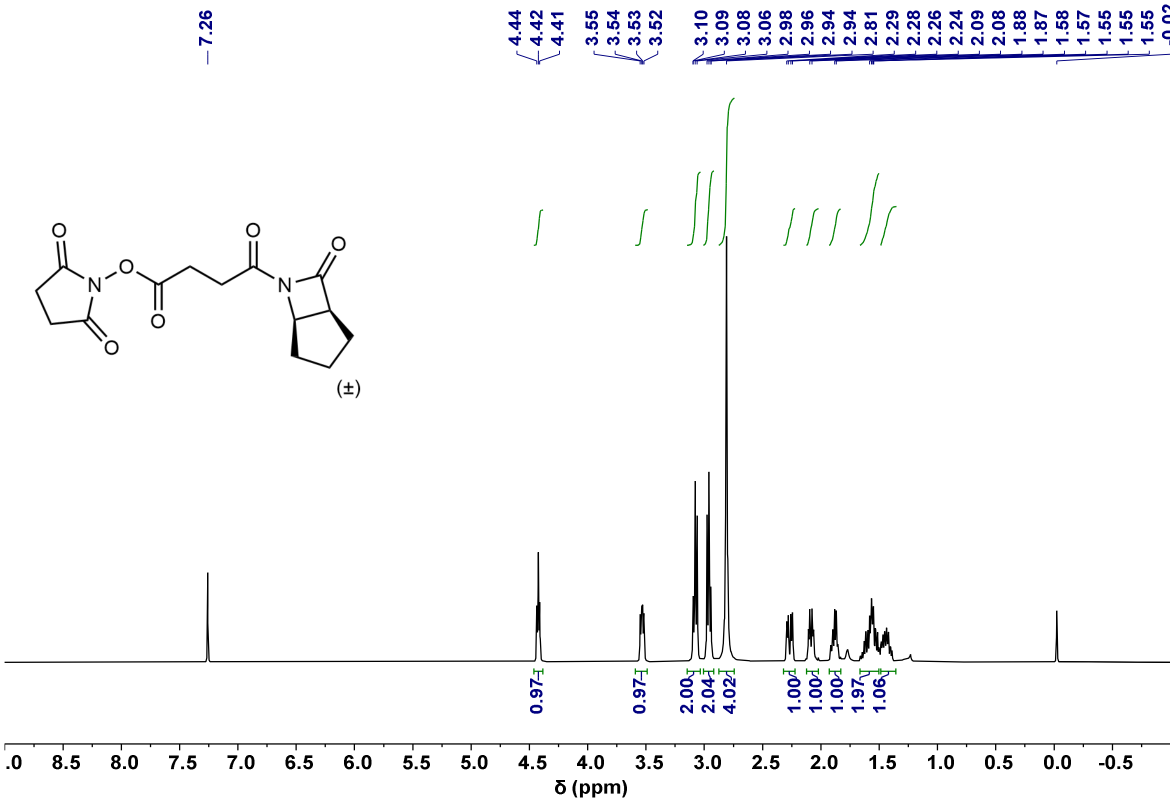

Supplementary Figure 46. ^{1}H NMR spectrum of NHS-CPβ in $CDCl_3$, 400 MHz.

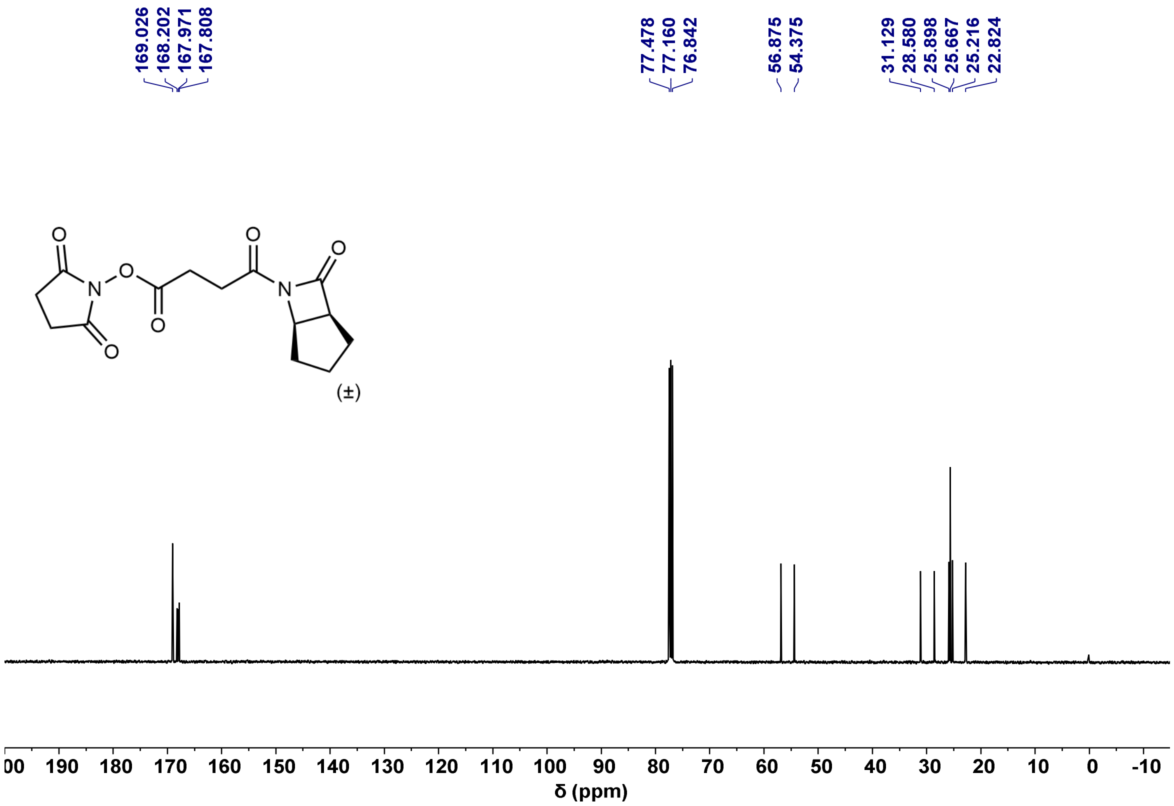

Supplementary Figure 47. ^{13}C NMR spectrum for in NHS-CPβ in $CDCl_3$, 100MHz.

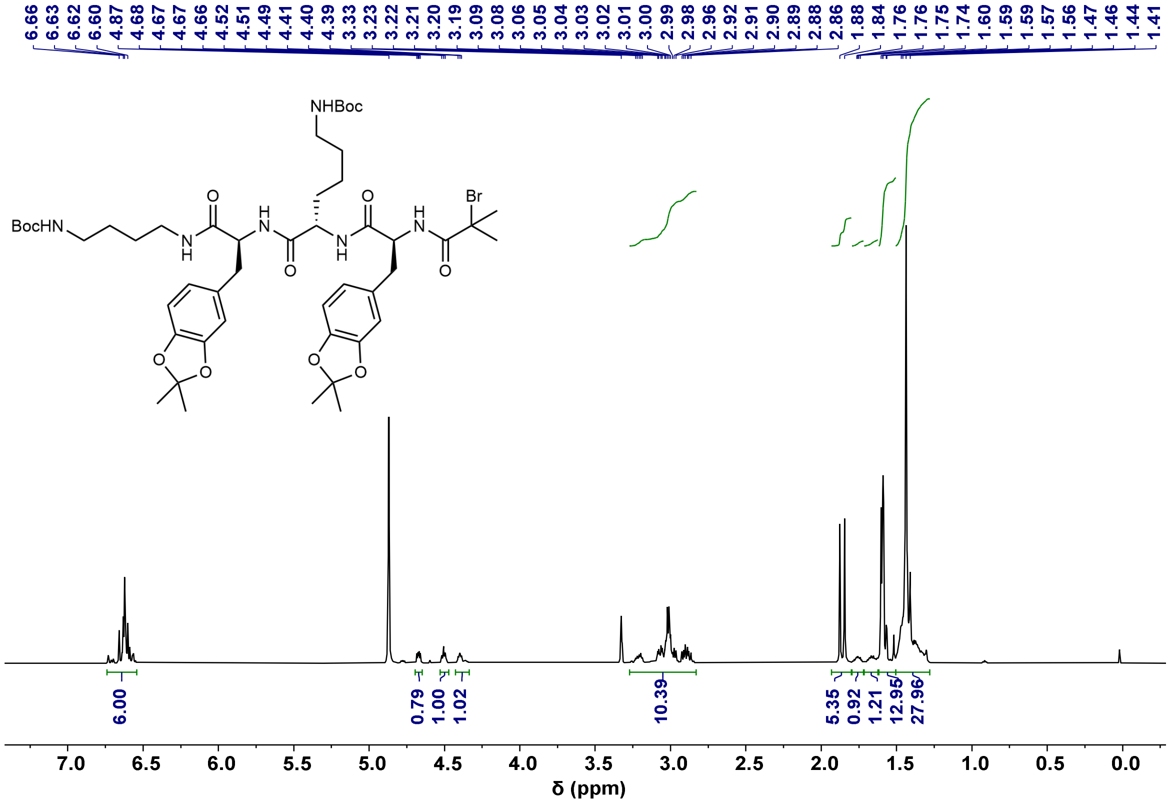

Supplementary Figure 48. ^{1}H NMR spectrum of compound **8** in CD_3OD, 600 MHz.

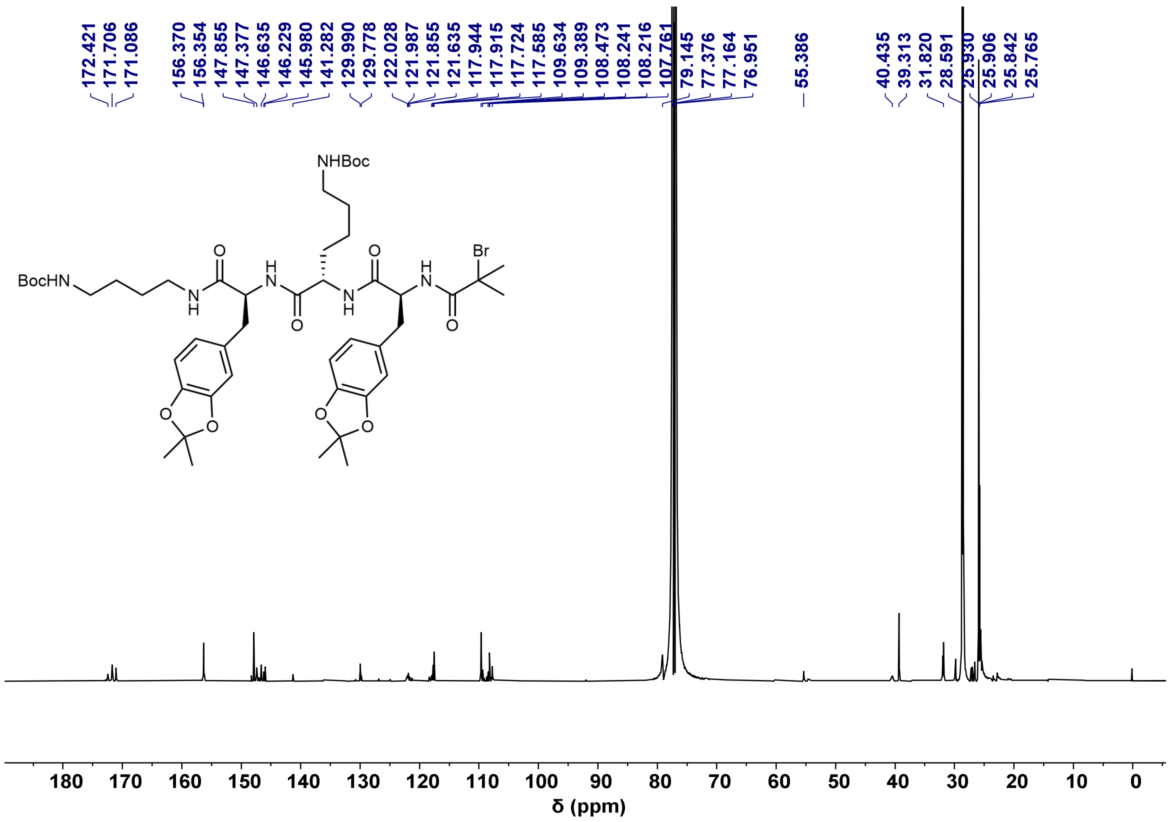

Supplementary Figure 49. ^{13}C NMR spectrum of compound **8** in $CDCl_3$, 150 MHz.

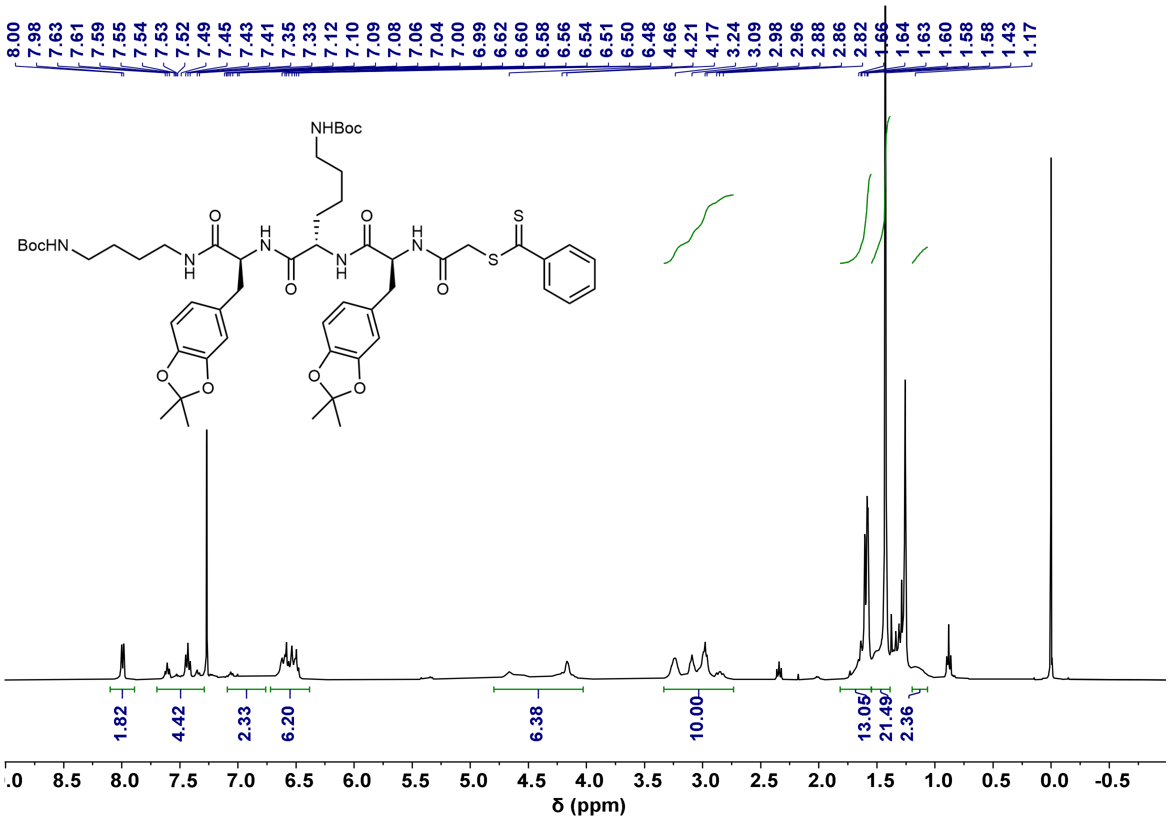

Supplementary Figure 50. ^{1}H NMR spectrum of compound **9** in $CDCl_3$, 400 MHz.

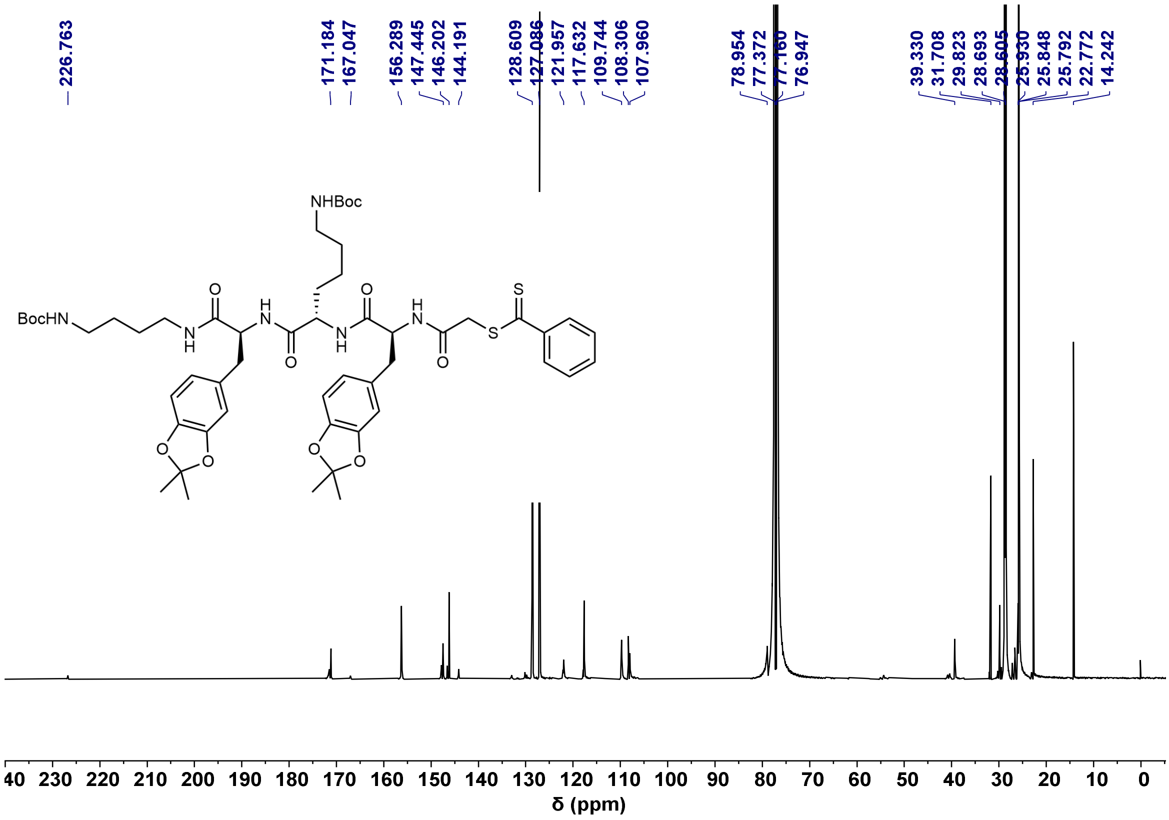

Supplementary Figure 51. ^{13}C NMR spectrum of compound **9** in $CDCl_3$, 150 MHz.

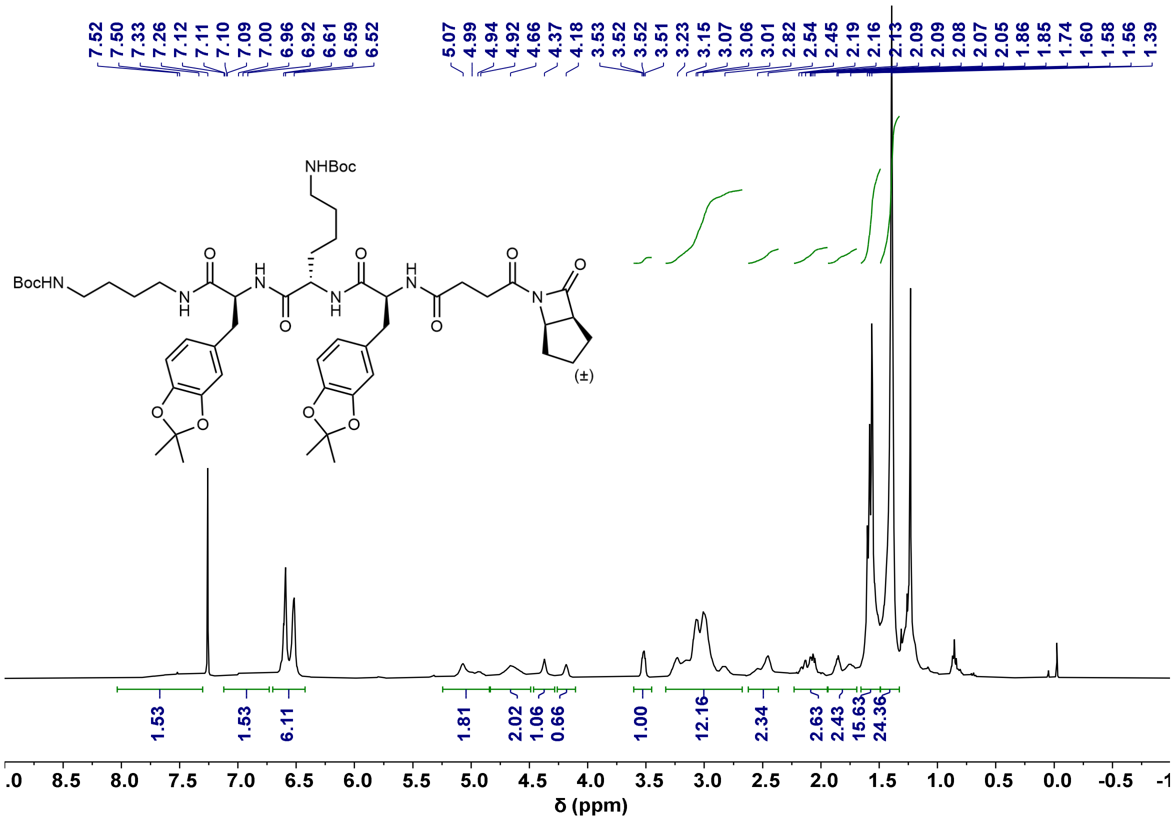

Supplementary Figure 52. ^{1}H NMR spectrum of compound **10** in $CDCl_3$, 400 MHz.

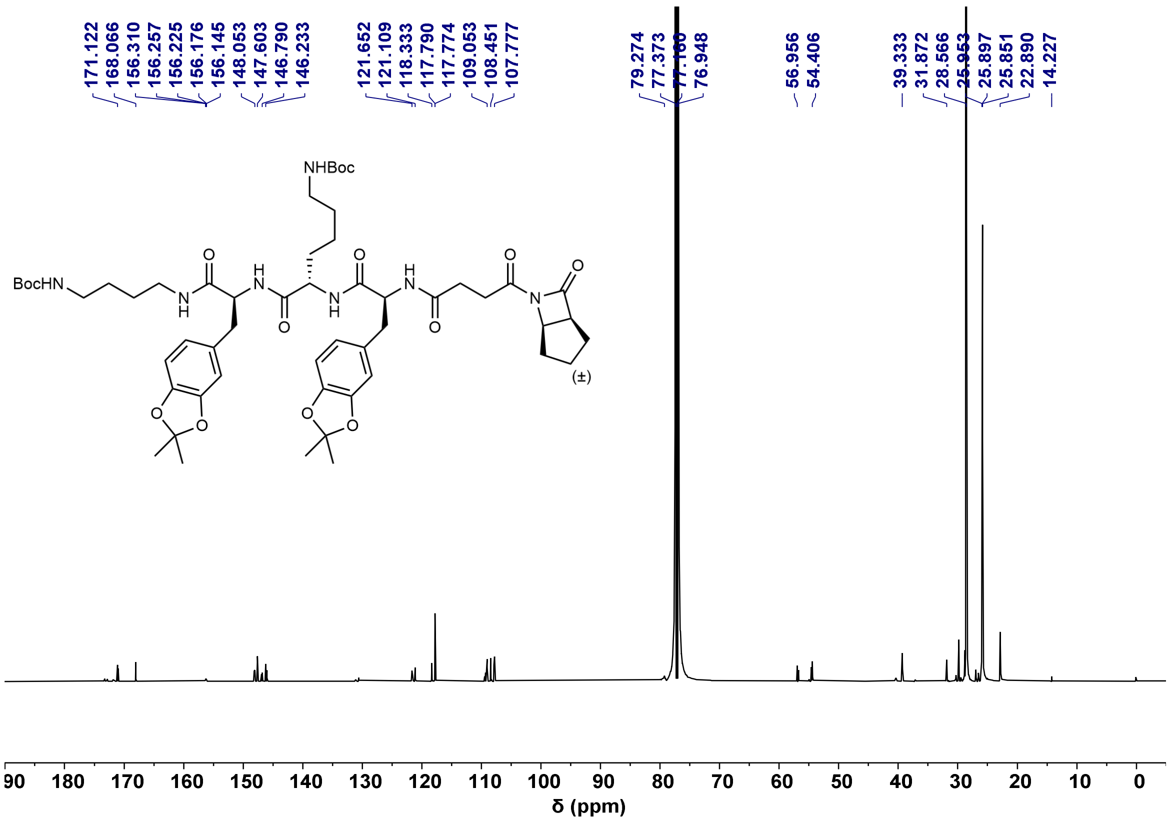

Supplementary Figure 53. ^{13}C NMR spectrum of compound **10** in $CDCl_3$, 150 MHz.

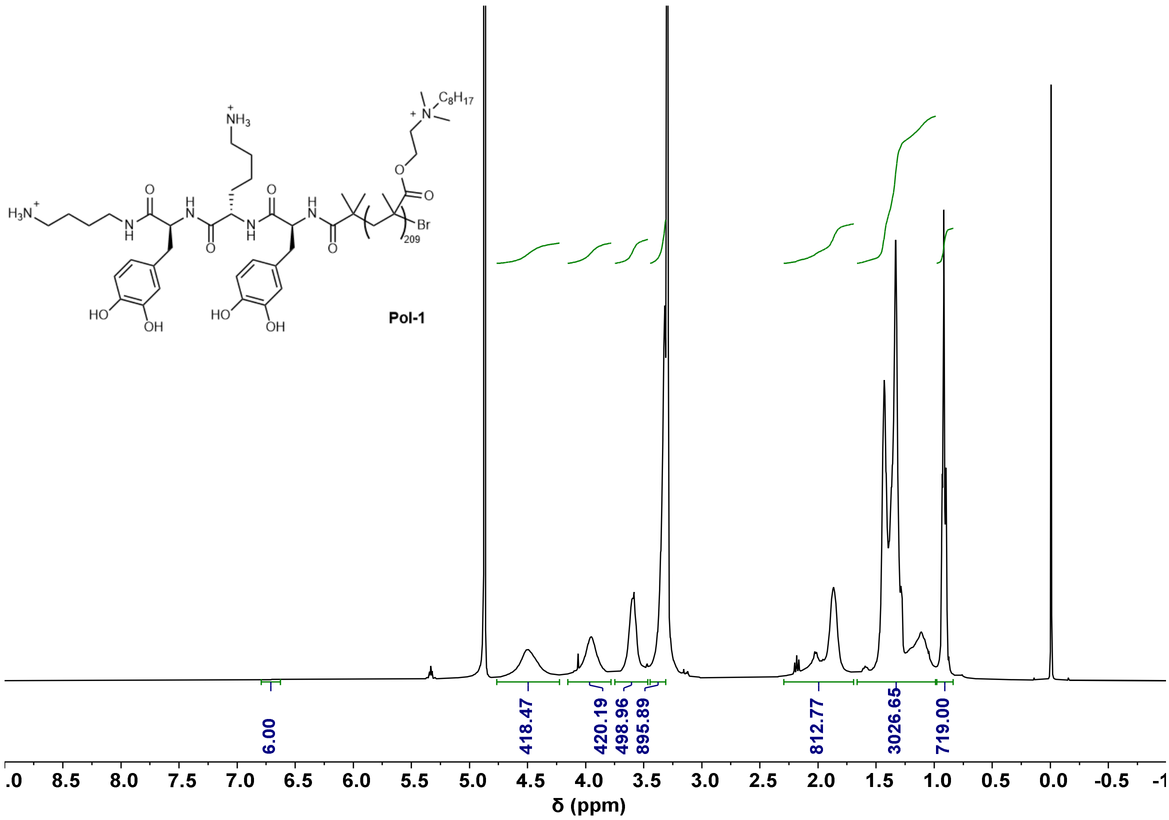

Supplementary Figure 54. ^{1}H NMR of Pol-1 in CD_3OD, 400 MHz.

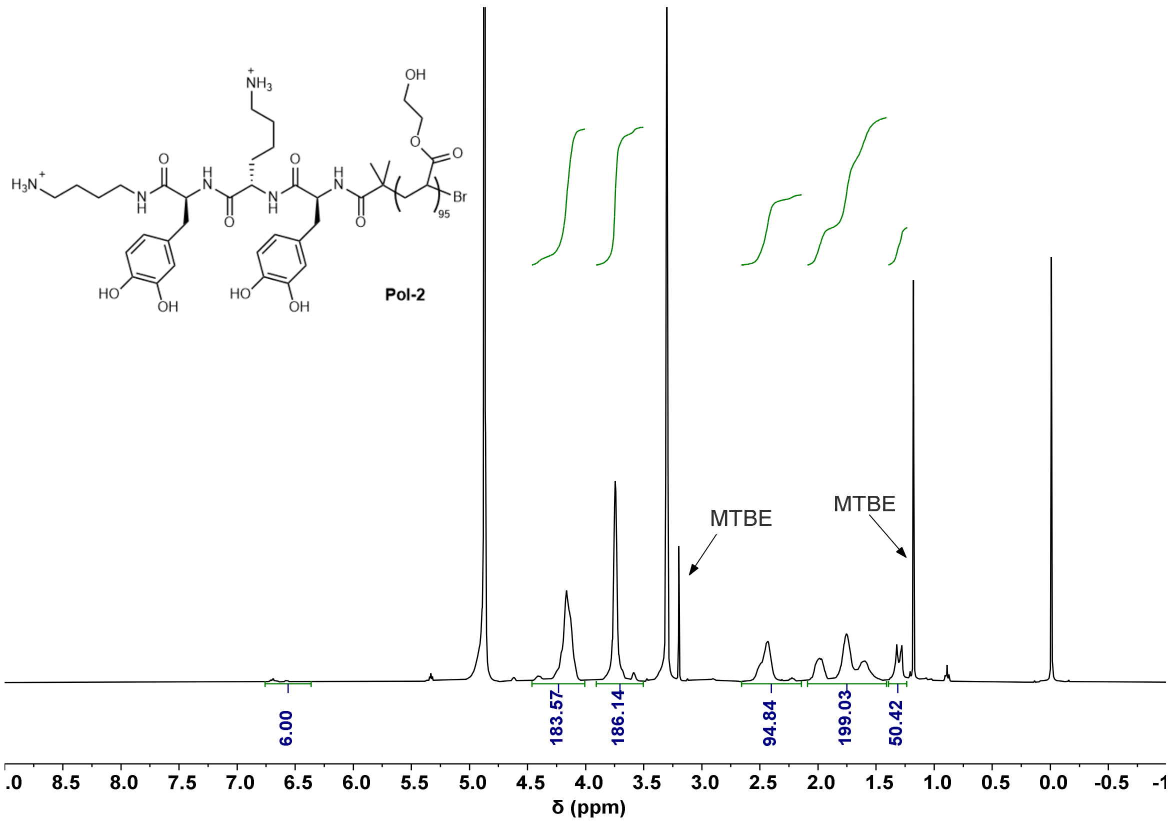

Supplementary Figure 55. ^{1}H NMR of Pol-2 in CD_3OD, 400 MHz. (MTBE: methyl tert-butyl ether used for polymer precipitation)

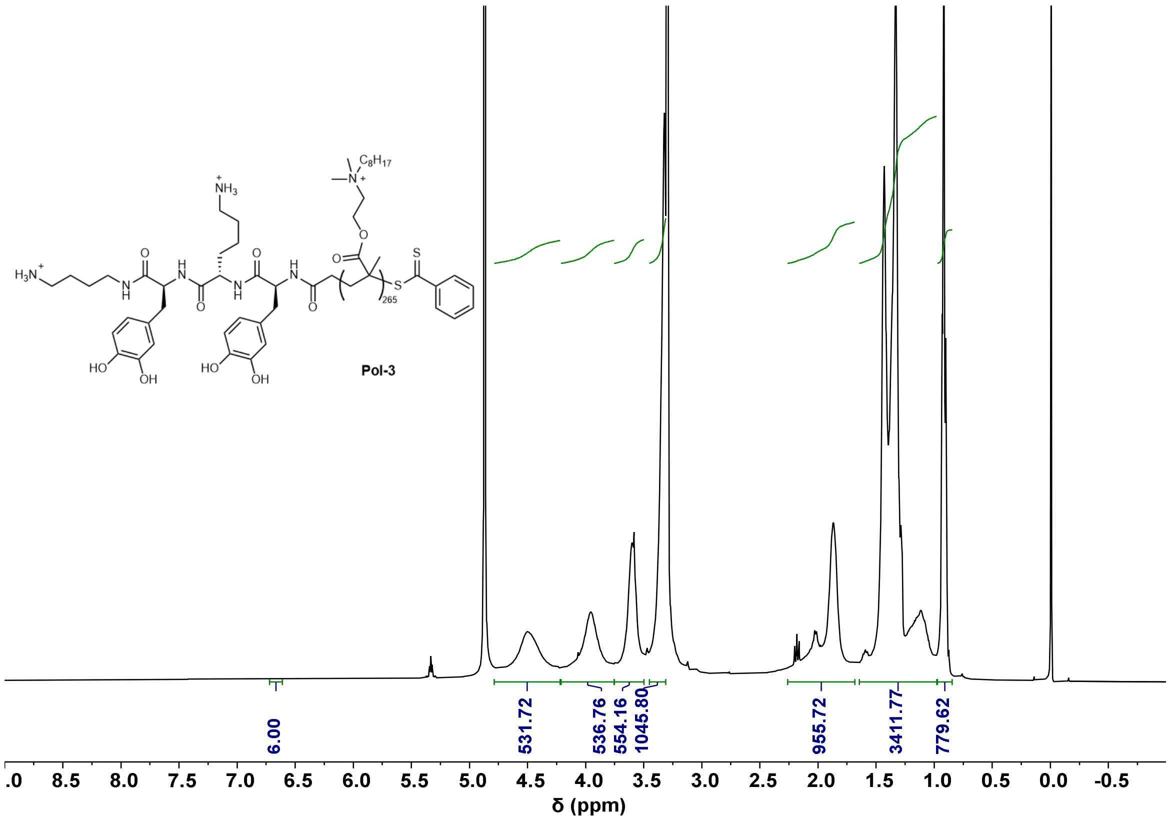

Supplementary Figure 56. ^{1}H NMR of Pol-3 in CD_3OD, 400 MHz.

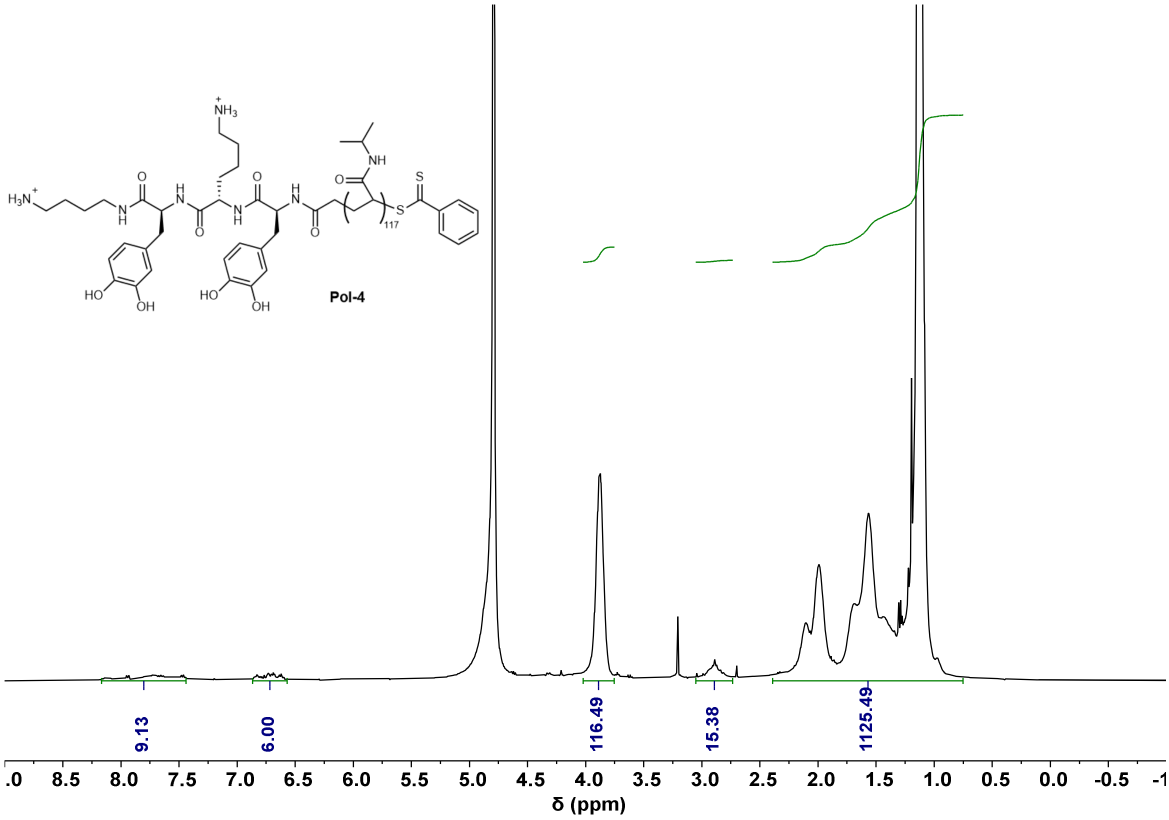

Supplementary Figure 57. ^{1}H NMR of Pol-4 in D_2O, 400 MHz.

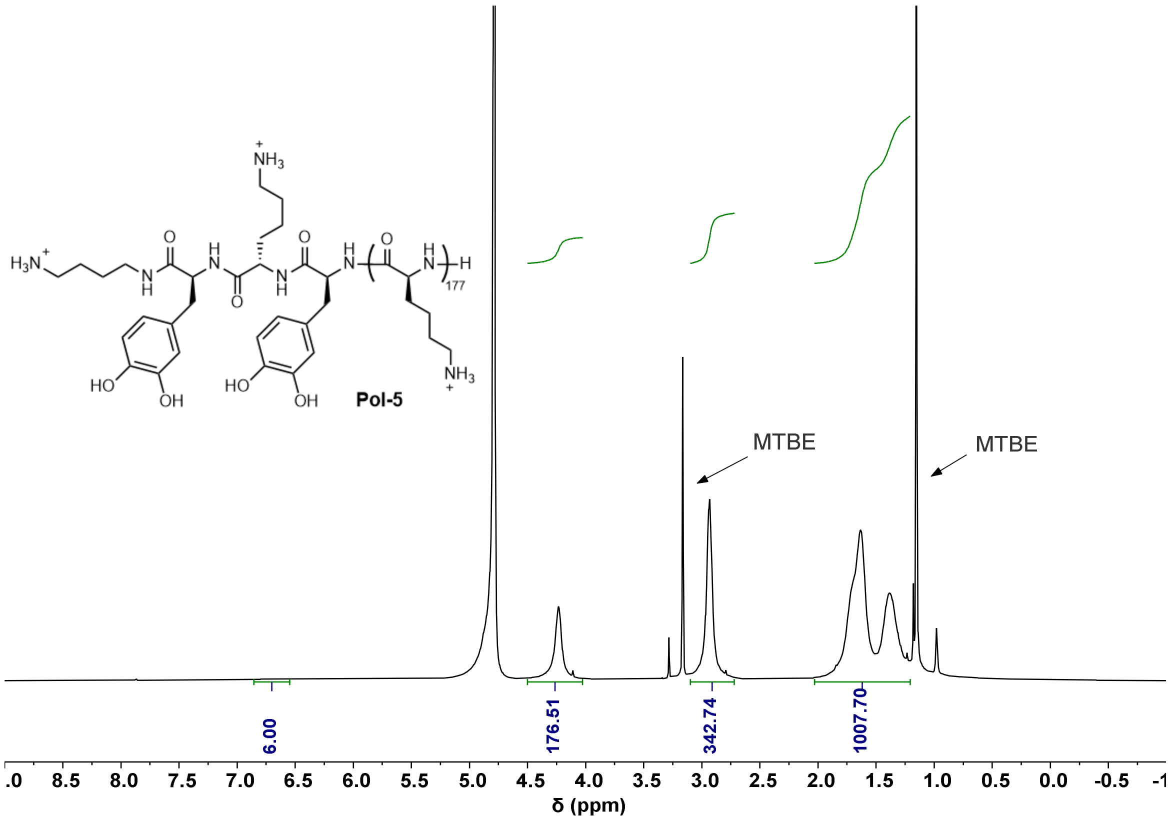

Supplementary Figure 58. ^{1}H NMR of Pol-5 in D_2O, 400 MHz. (MTBE: methyl tert-butyl ether used for polymer precipitation)

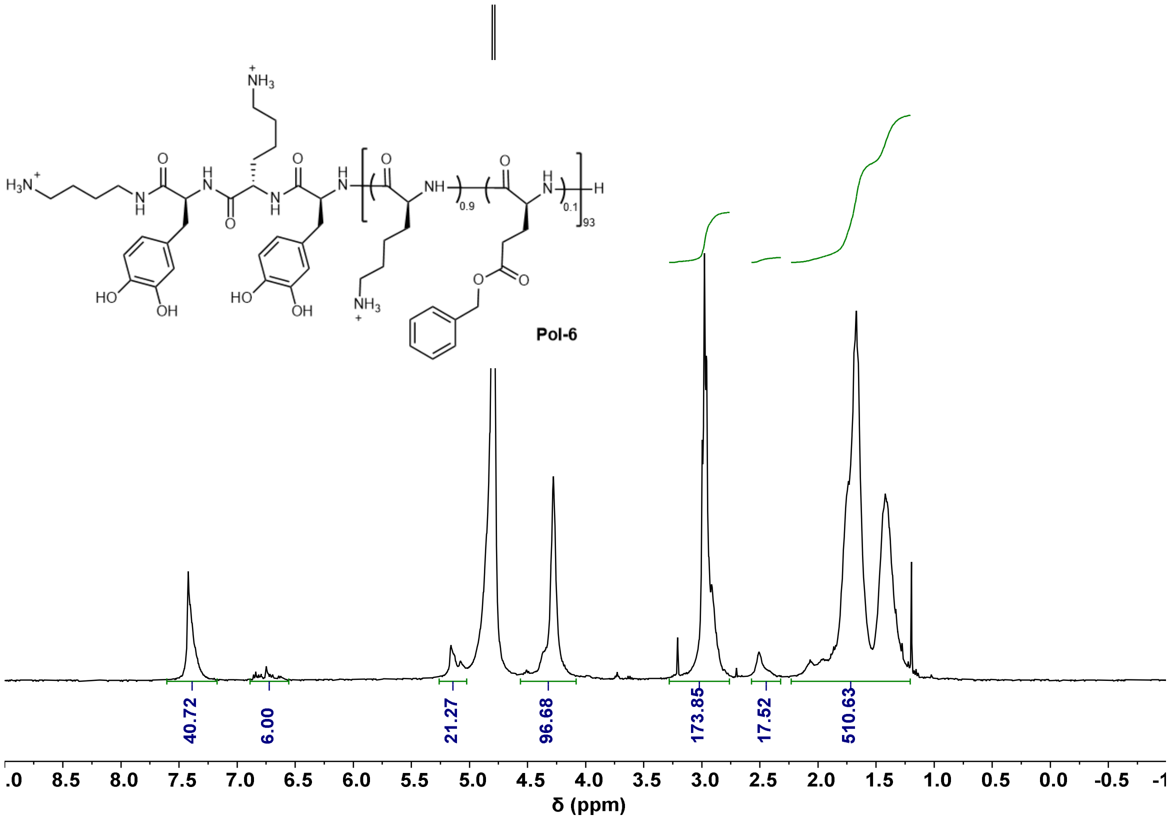

Supplementary Figure 59. ^{1}H NMR of Pol-6 in D_2O, 400 MHz.

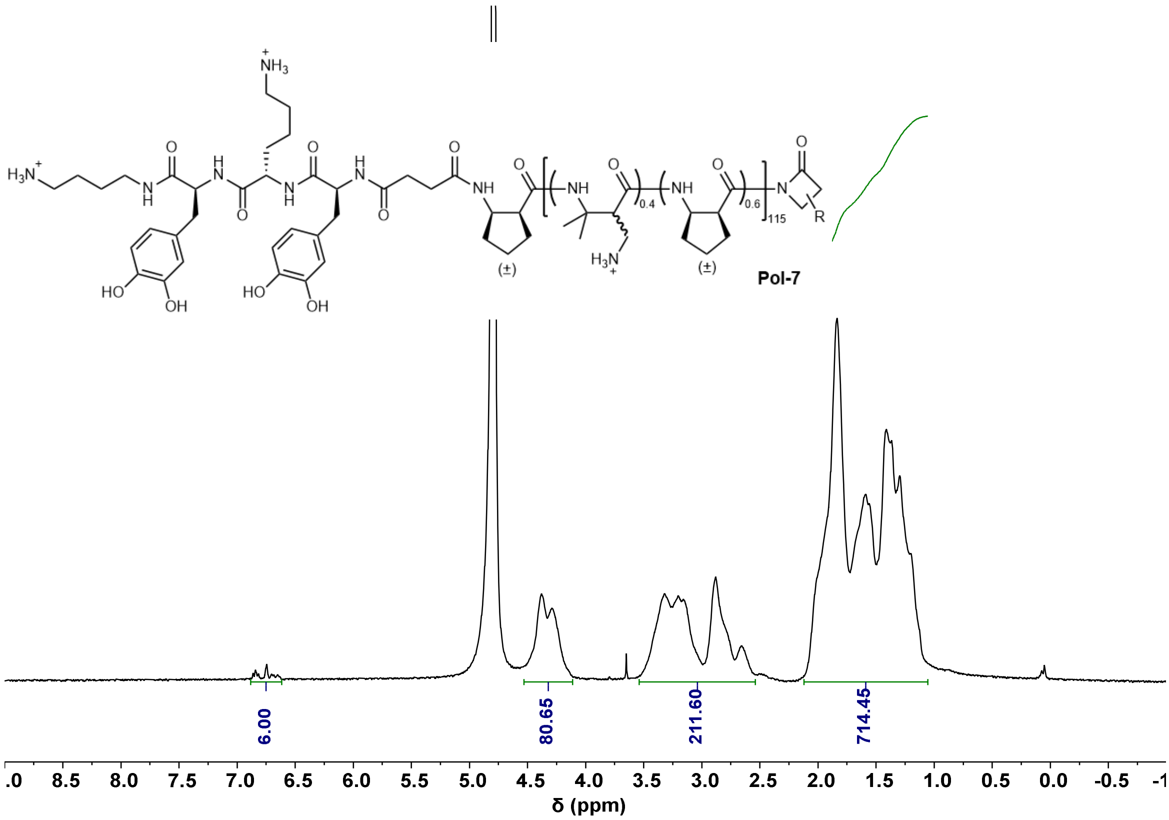

Supplementary Figure 60. ^{1}H NMR of Pol-7 in D_2O, 400 MHz.

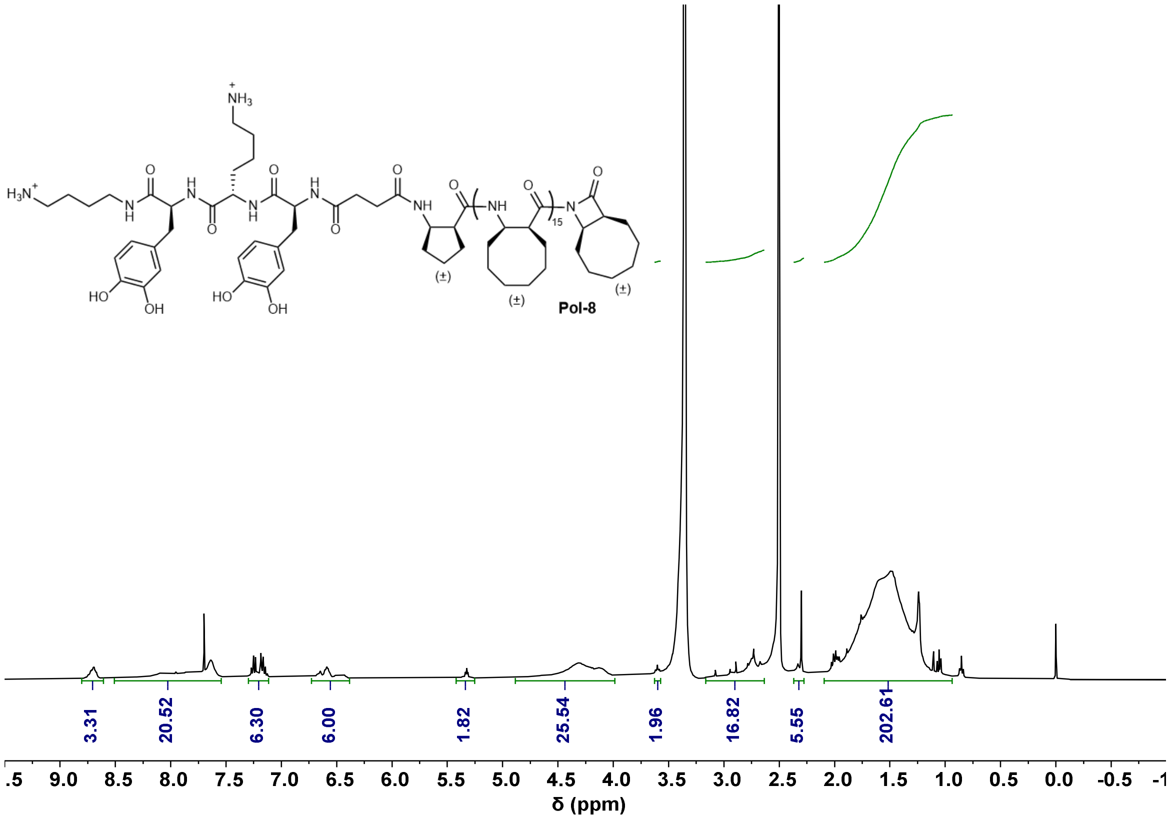

Supplementary Figure 61. ^{1}H NMR of Pol-8 in DMSO-*d*6, 400 MHz.

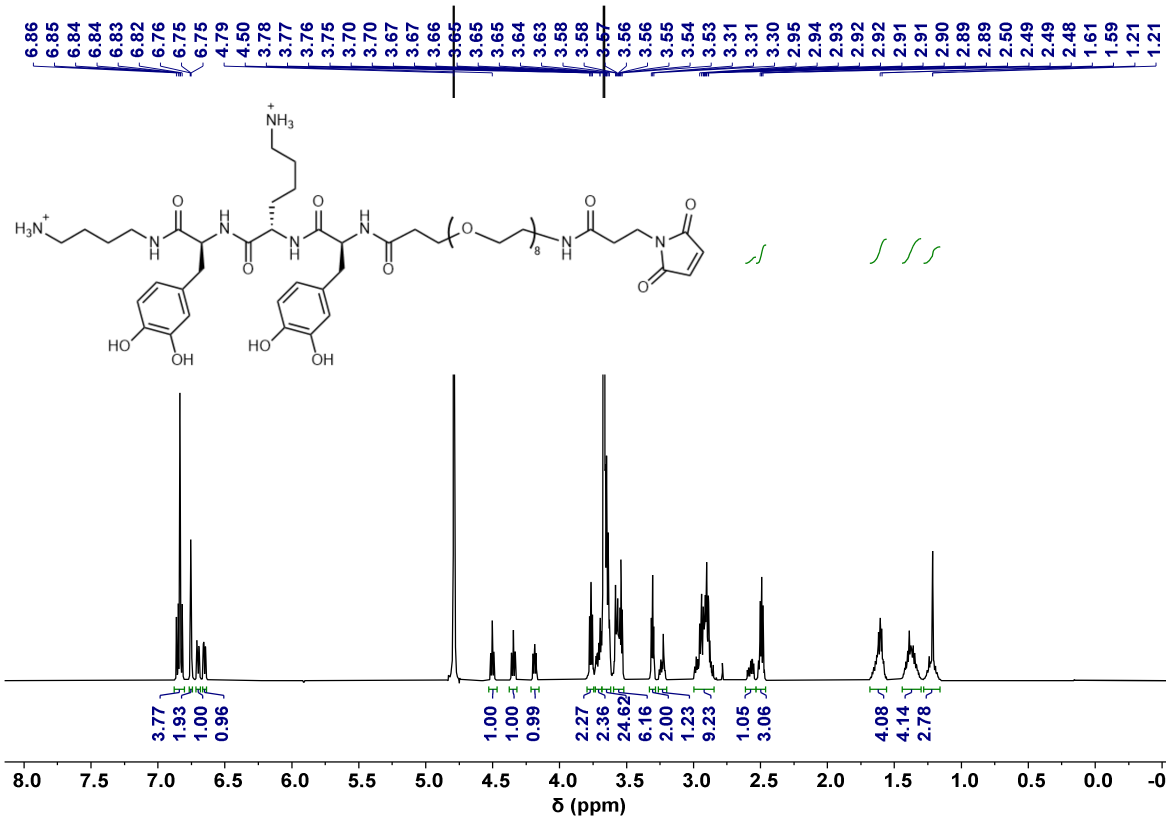

Supplementary Figure 62. ^{1}H NMR spectrum for DbaYKY-OEG8-Mal in D_2O, 600 MHz.

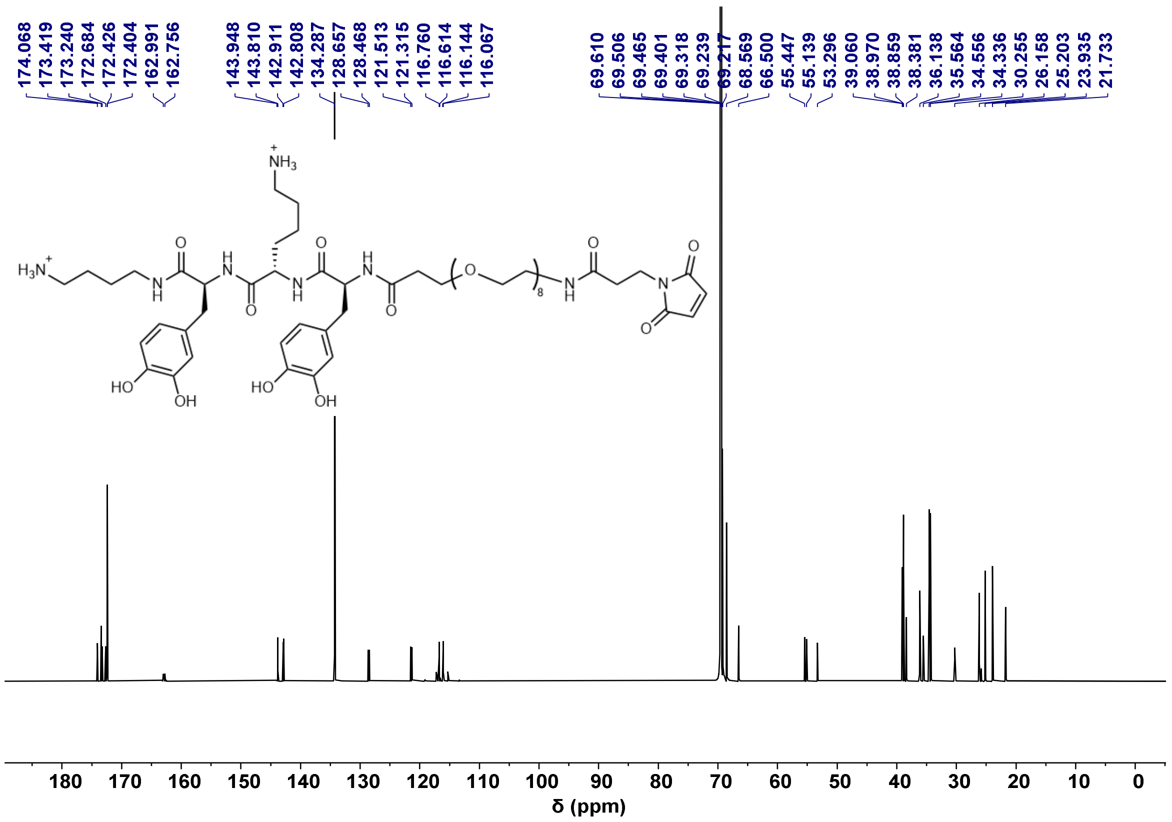

Supplementary Figure 63. ^{13}C NMR spectrum for DbaYKY-OEG8-Mal in D_2O, 150 MHz.

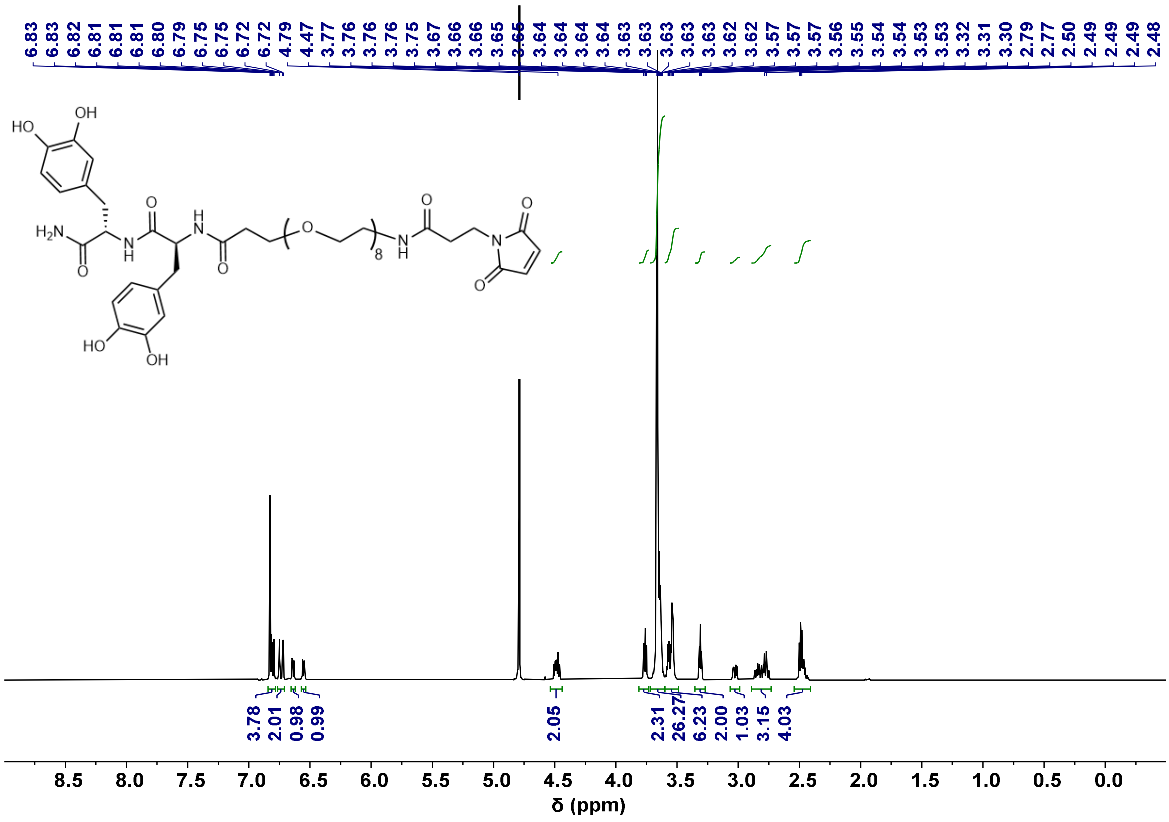

Supplementary Figure 64. ^{1}H NMR spectrum for YY-OEG8-Mal in D_2O, 600 MHz.

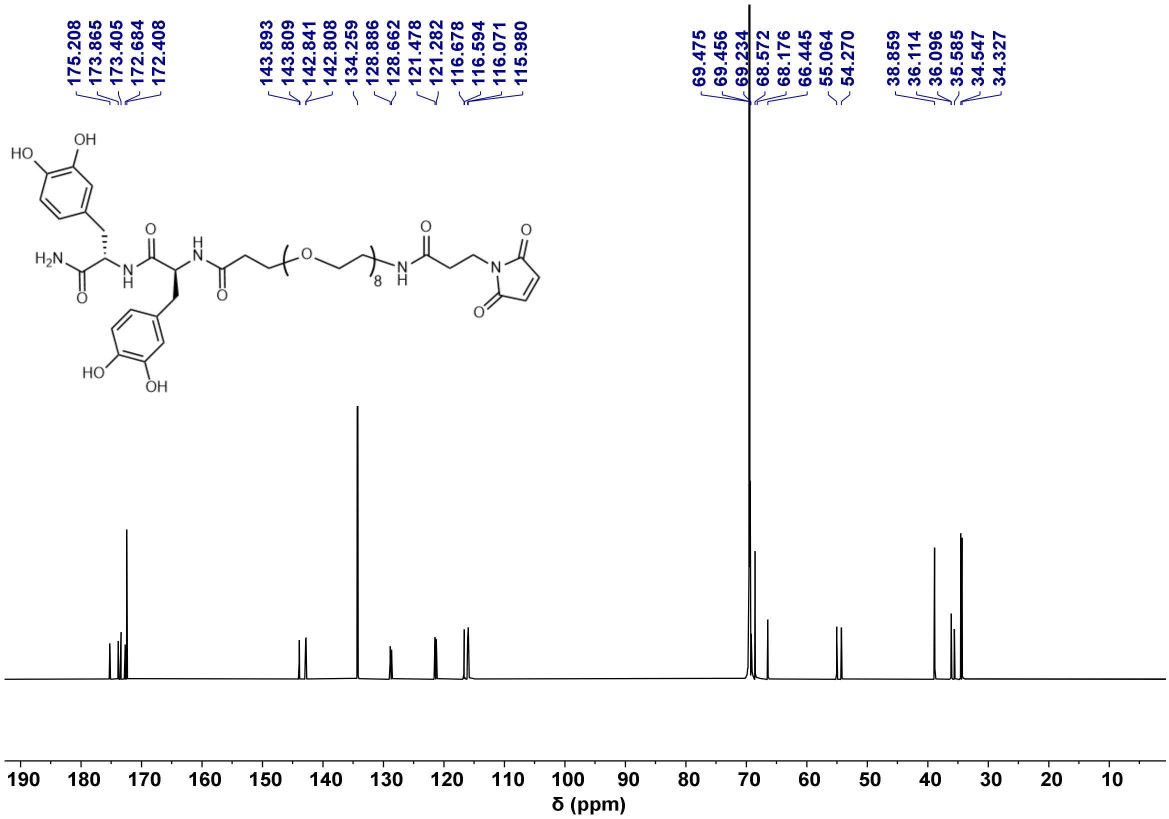

Supplementary Figure 65. ^{13}C NMR spectrum for YY-OEG8-Mal in D_2O, 150 MHz.

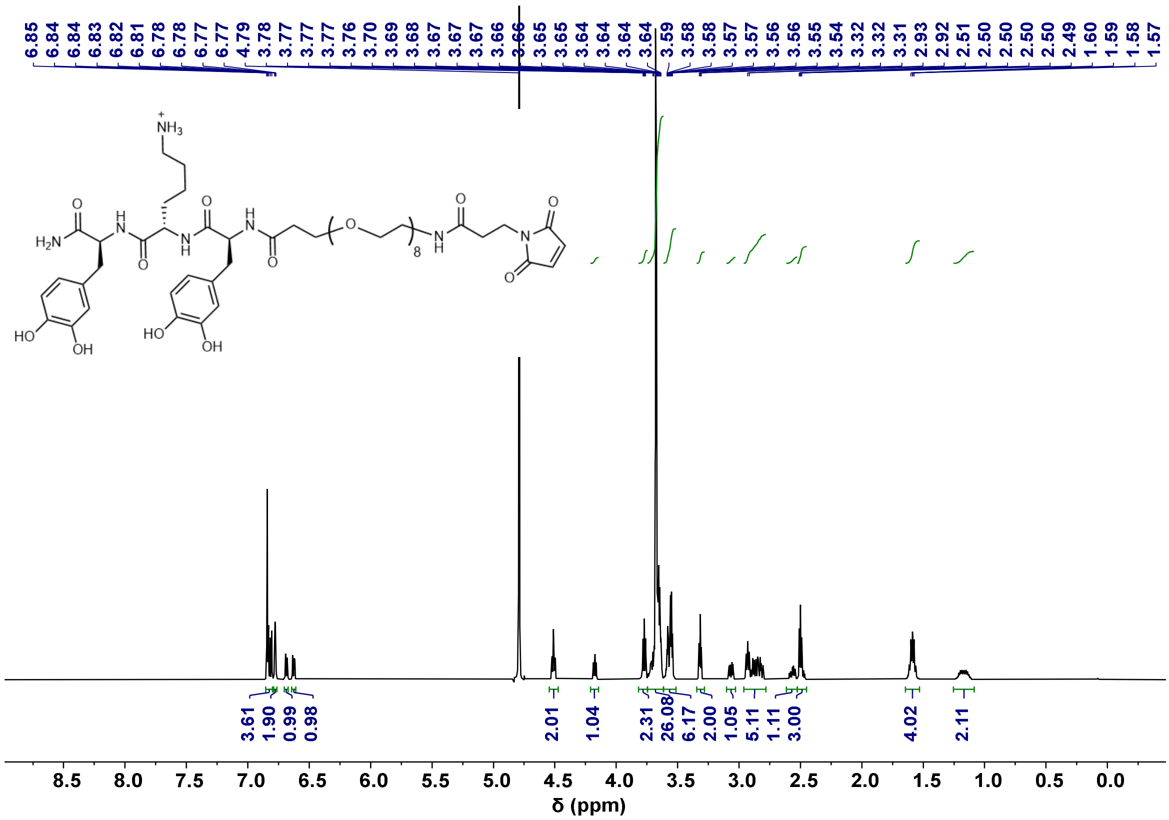

Supplementary Figure 66. ^{1}H NMR spectrum for YKY-OEG8-Mal in D_2O, 600 MHz.

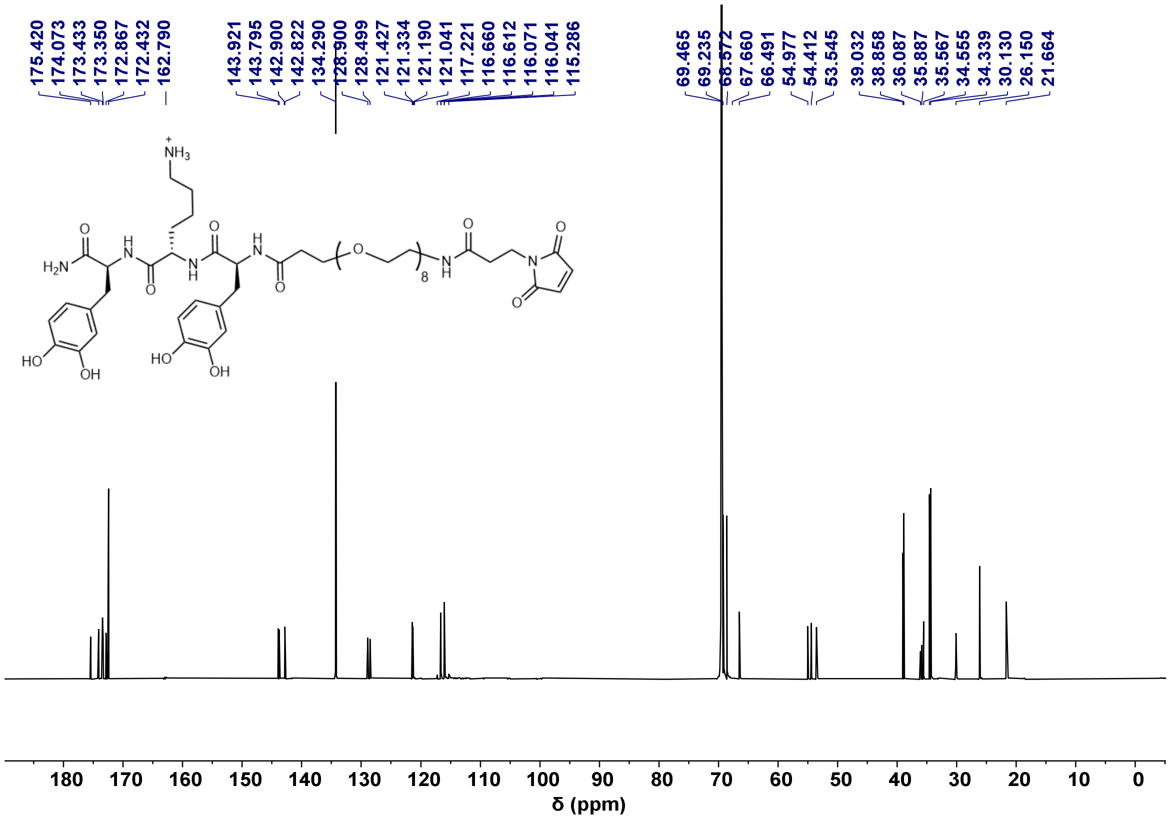

Supplementary Figure 67. ^{13}C NMR spectrum for YKY-OEG8-Mal in D_2O, 150 MHz.

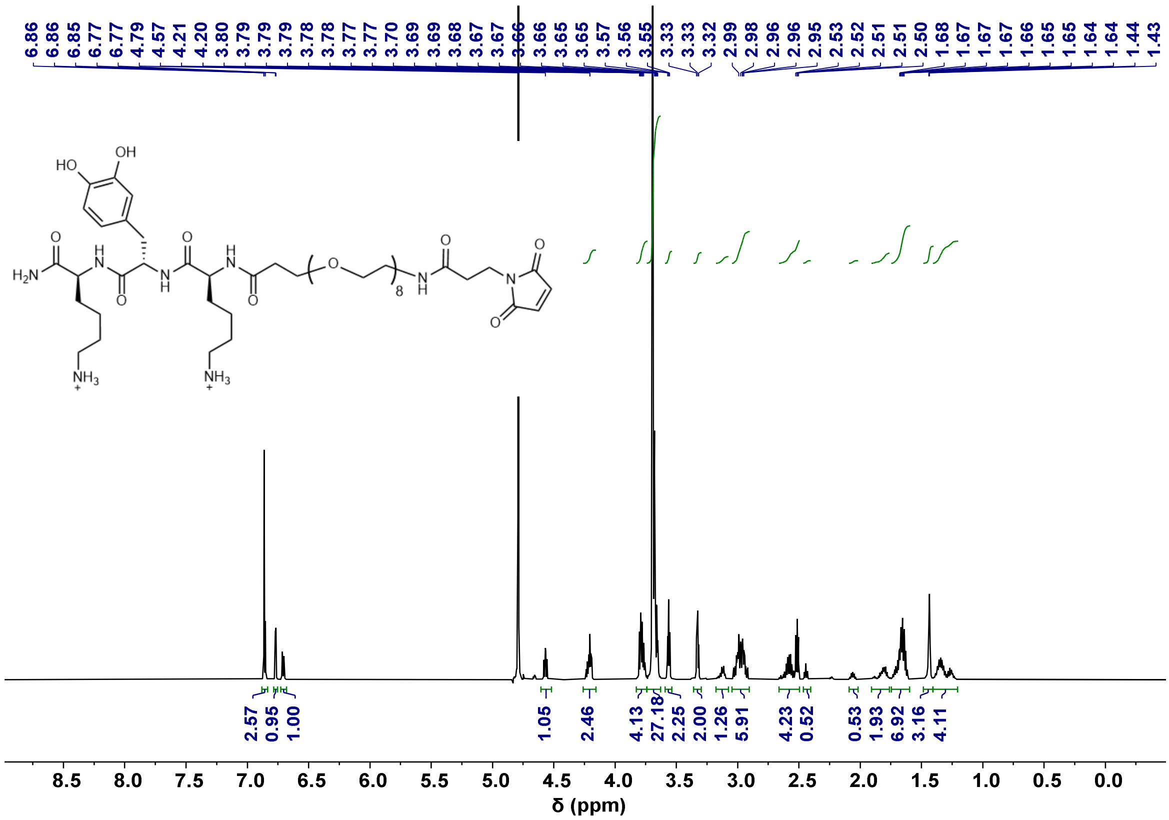

Supplementary Figure 68. ^{1}H NMR spectrum for K<u>Y</u>KOEG8-Mal in D_2O, 600 MHz.

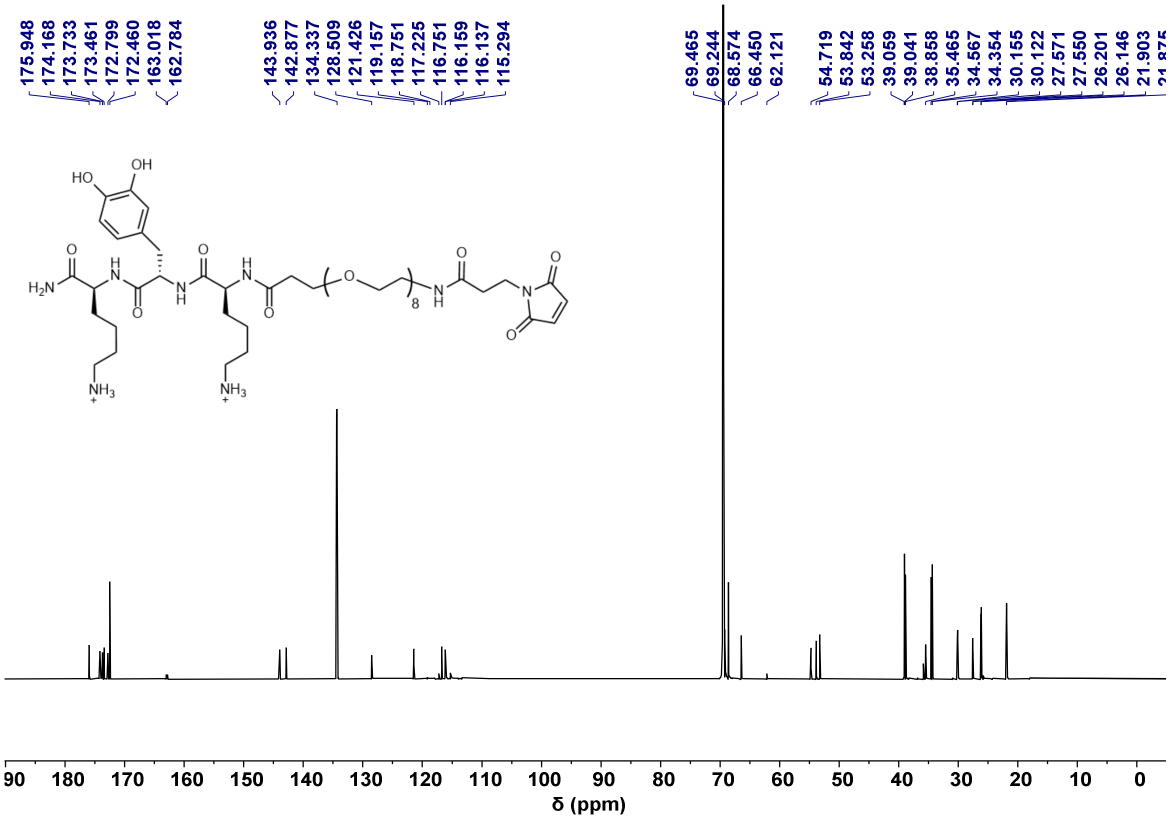

Supplementary Figure 69. ^{13}C NMR spectrum for K<u>Y</u>K-OEG8-Mal in D_2O, 150 MHz.

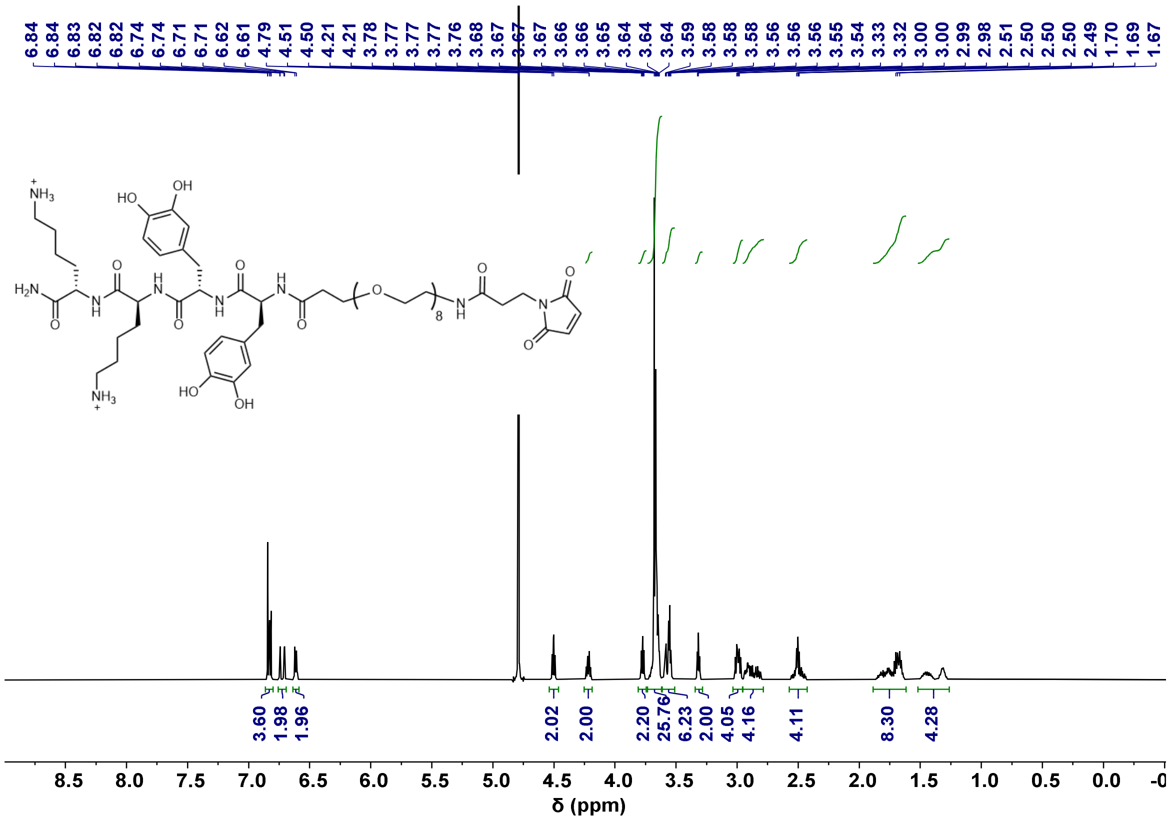

Supplementary Figure 70. ^{1}H NMR spectrum for KK<u>YY</u>-OEG8-Mal in D_2O, 600 MHz.

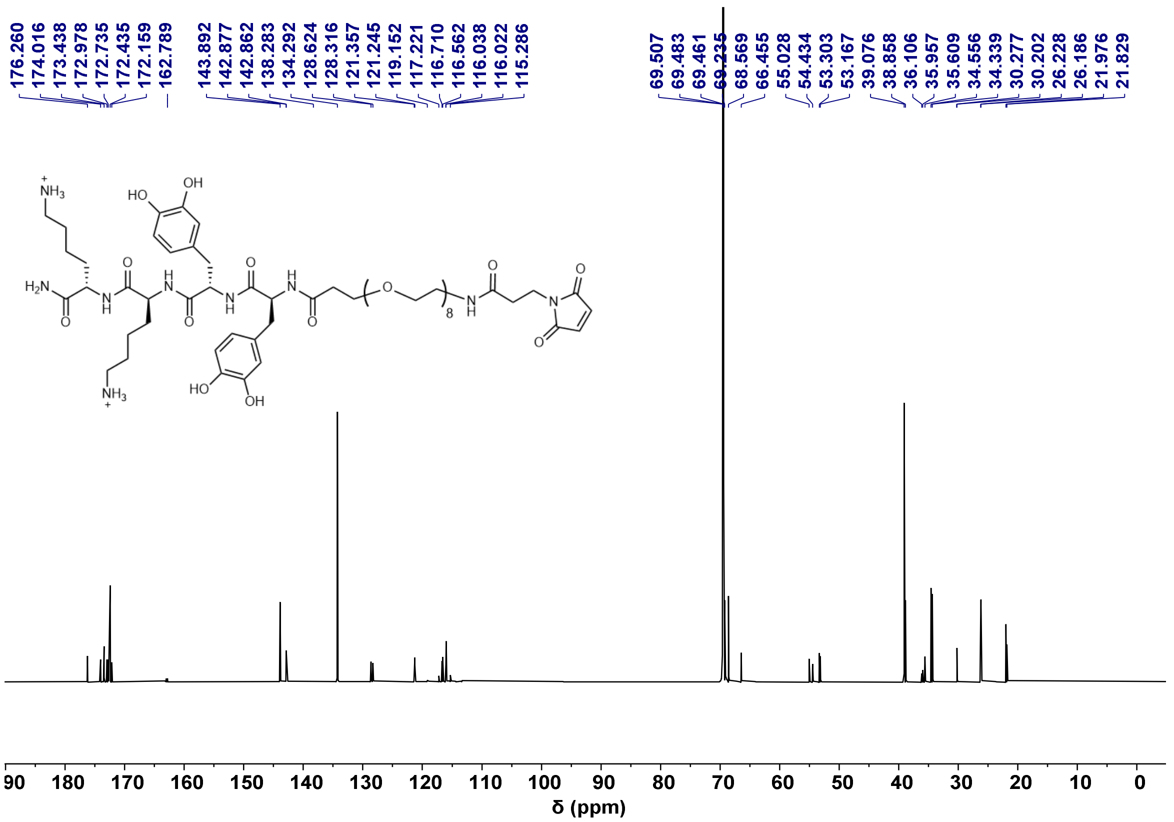

Supplementary Figure 71. ^{13}C NMR spectrum for KK<u>YY</u>-OEG8-Mal in D_2O, 150 MHz.

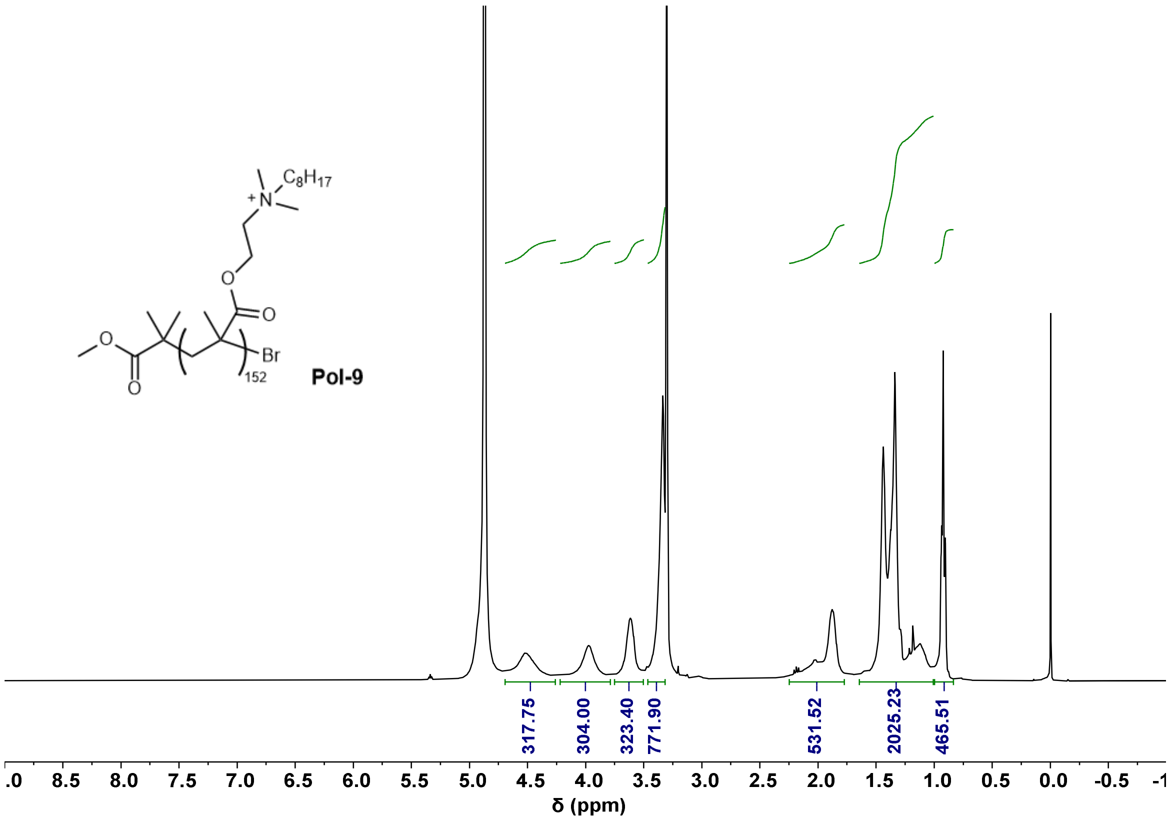

Supplementary Figure 72. ^{1}H NMR of Pol-9 in CD_3OD, 400 MHz.

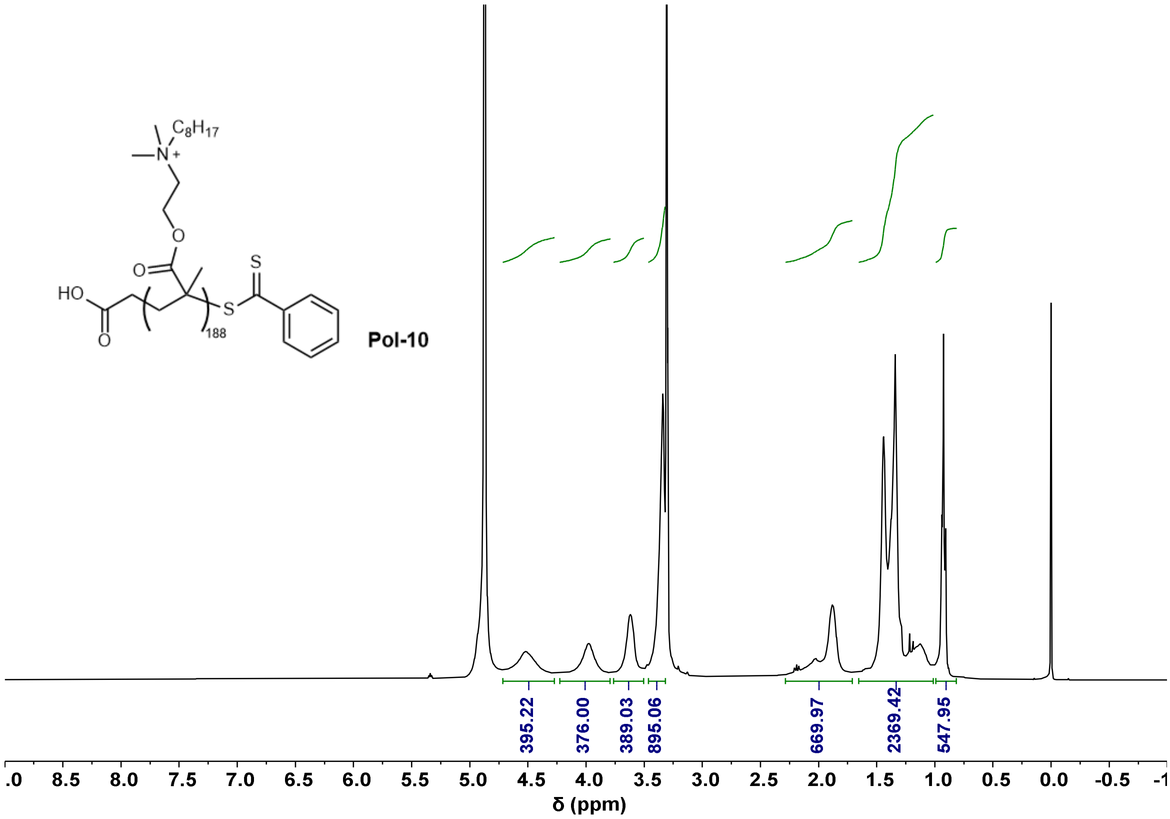

Supplementary Figure 73. ^{1}H NMR of Pol-10 in CD_3OD, 400 MHz.

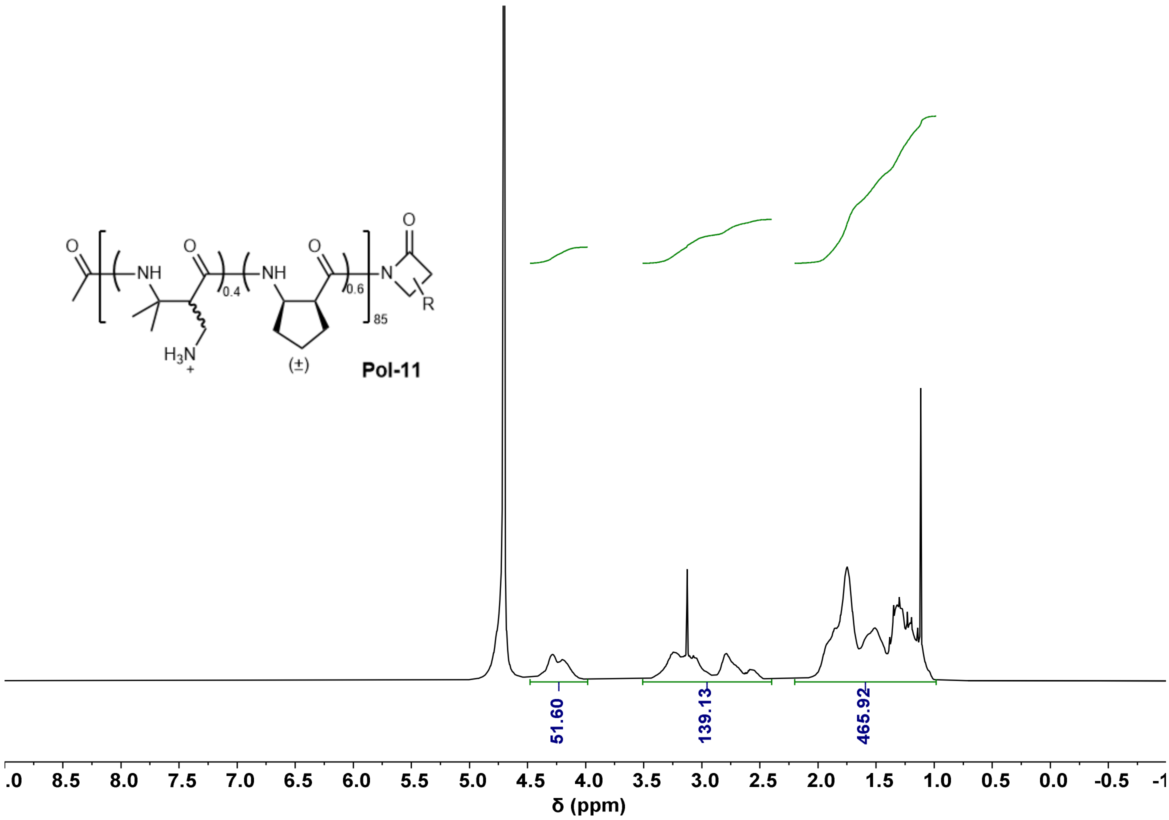

Supplementary Figure 74. ^{1}H NMR of Pol-11 in D_2O, 400 MHz.

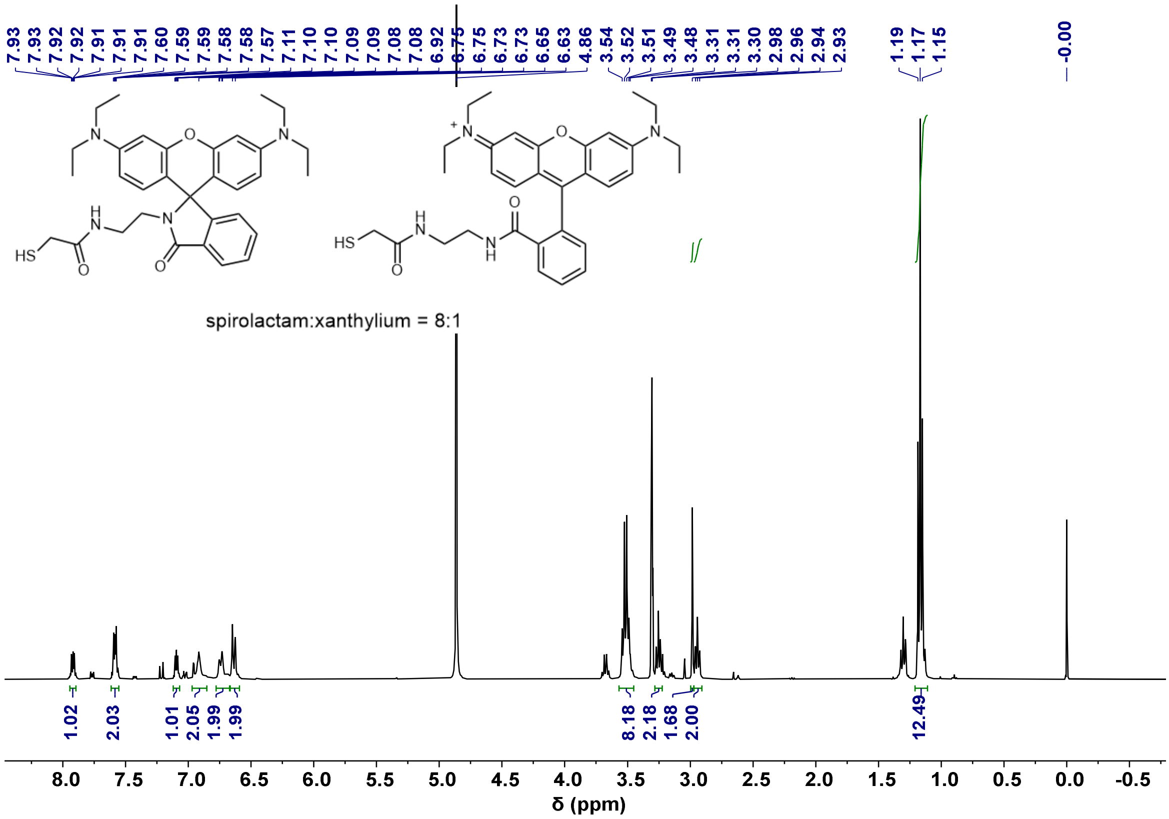

Supplementary Figure 75. ^{1}H NMR spectrum for Rh-SH in CD_3OD, 400 MHz.

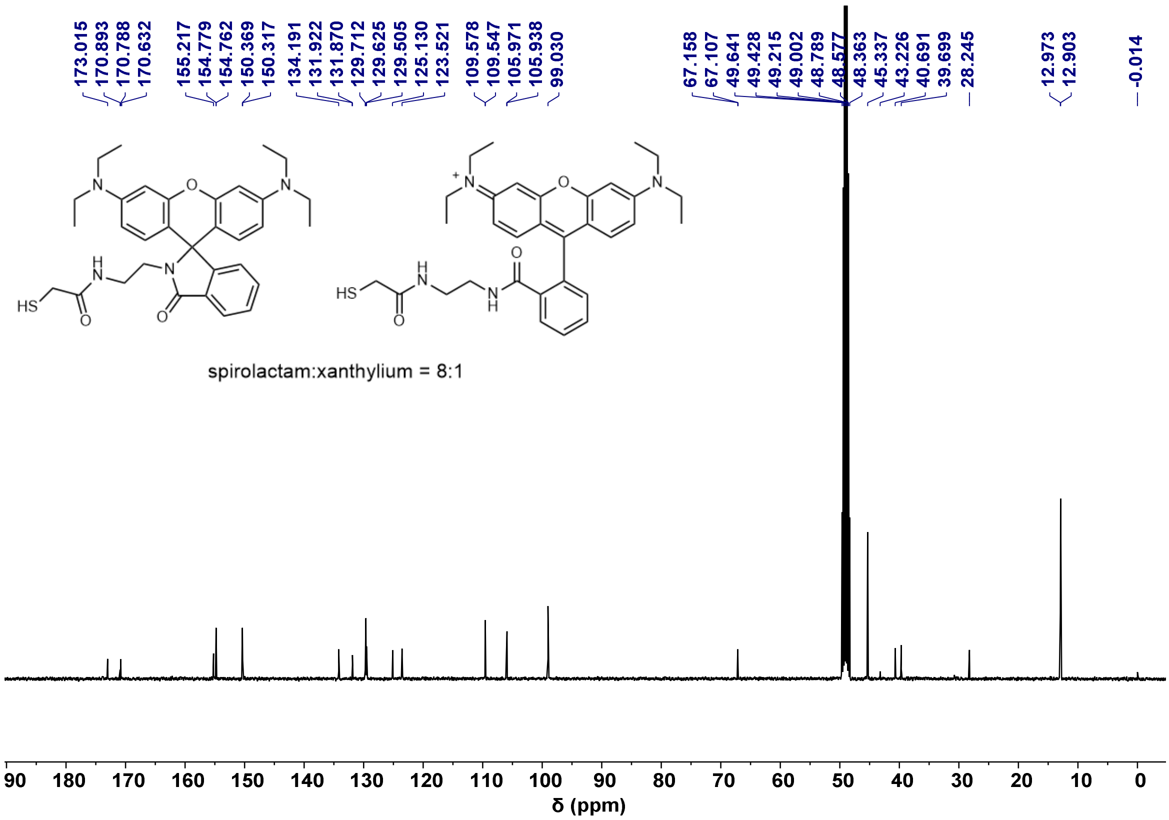

Supplementary Figure 76. ^{13}C NMR spectrum for Rh-SH in CD_3OD, 100 MHz.

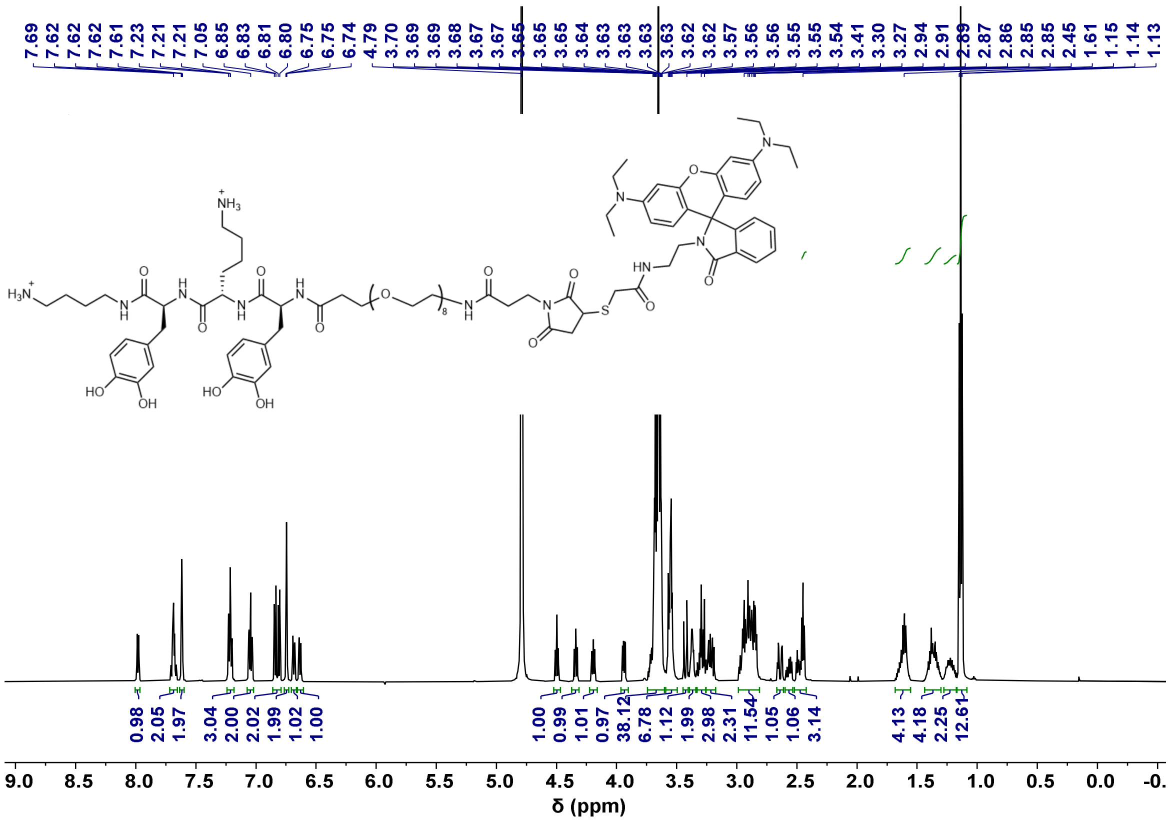

Supplementary Figure 77. ^{1}H NMR spectrum for Dba<u>Y</u>K<u>Y</u>-OEG8-Rh in D_2O, 600 MHz.

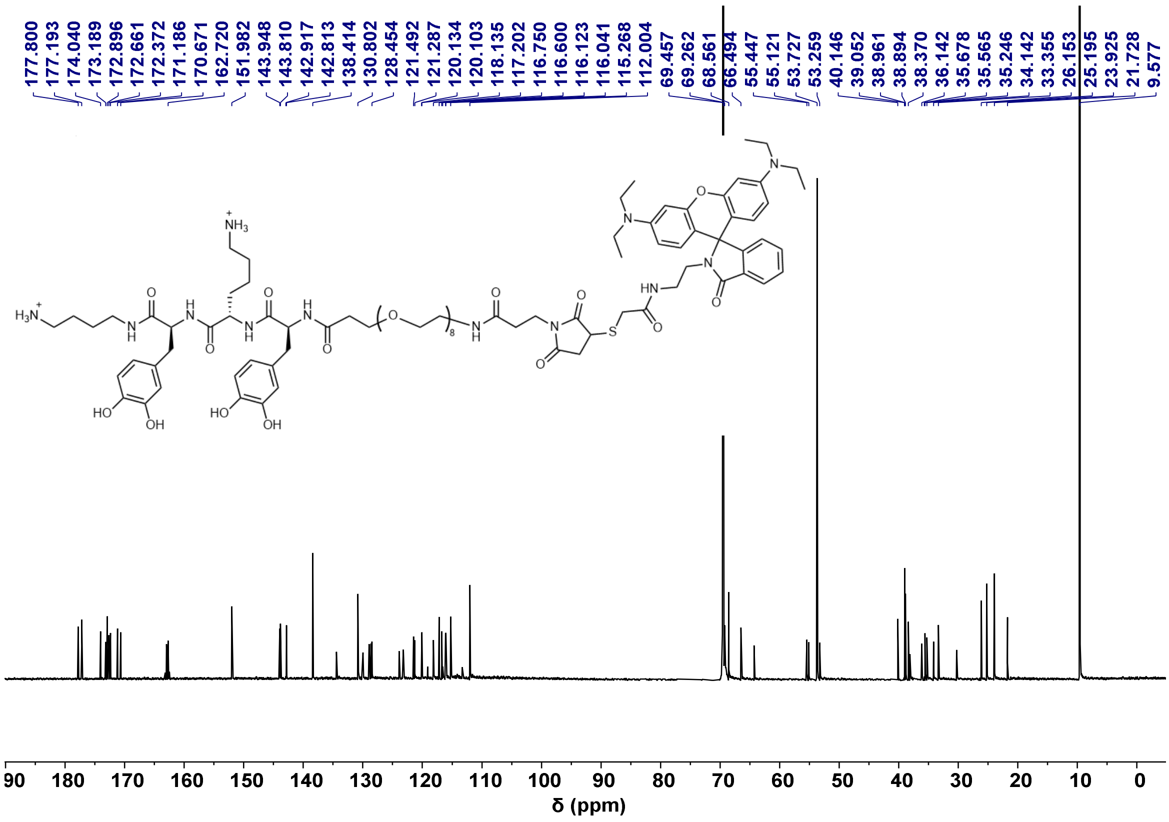

Supplementary Figure 78. ^{13}C NMR spectrum for DbaYKY-OEG8-Rh in CD_3OD, 150 MHz.

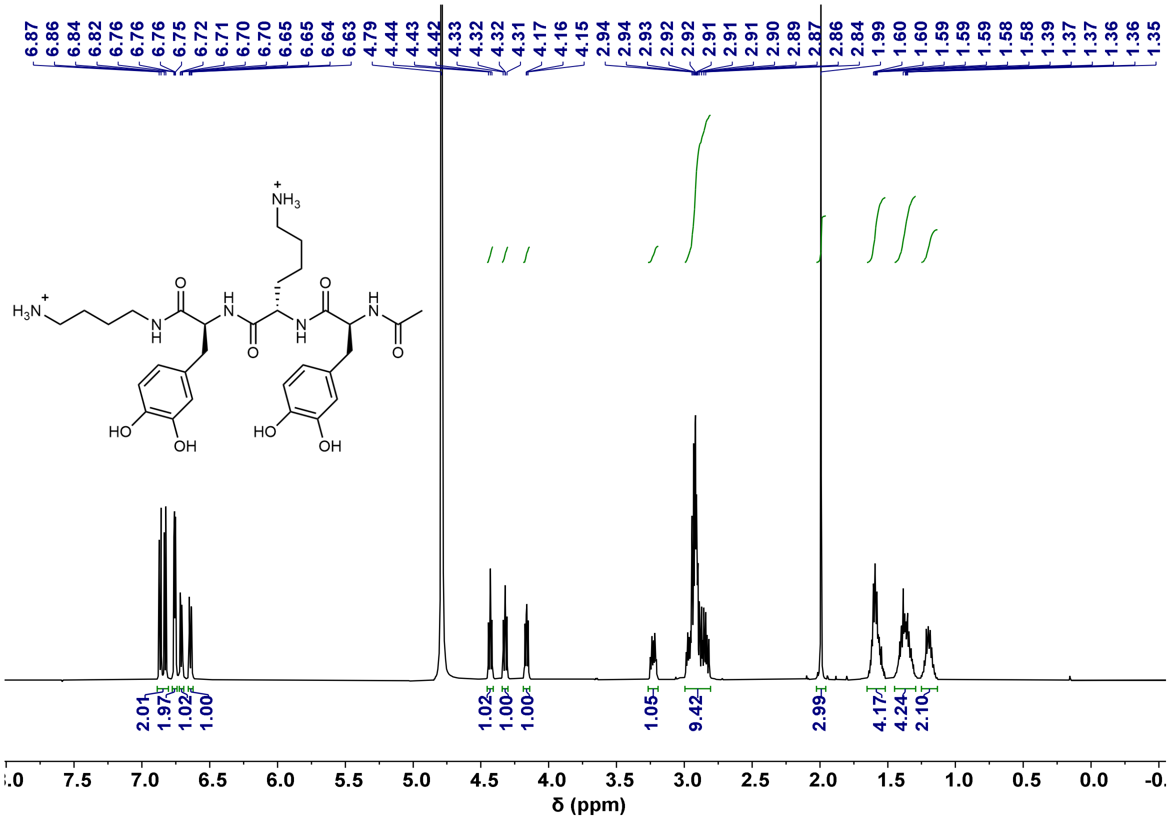

Supplementary Figure 79. ^{1}H NMR spectrum of DbaYKY-Ac in D_2O, 600 MHz.

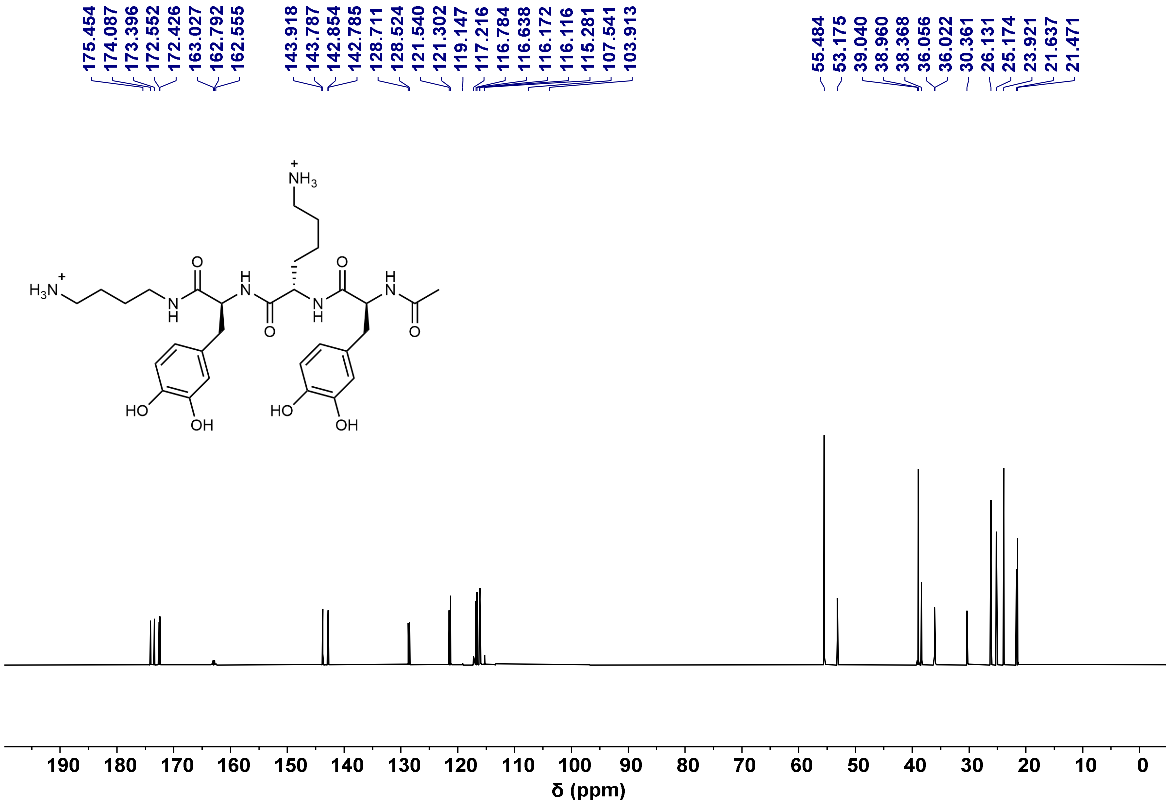

Supplementary Figure 80. ^{13}C NMR spectrum for Dba<u>Y</u>K<u>Y</u>-Ac in CD_3OD, 150 MHz.

GPC trace of polymers

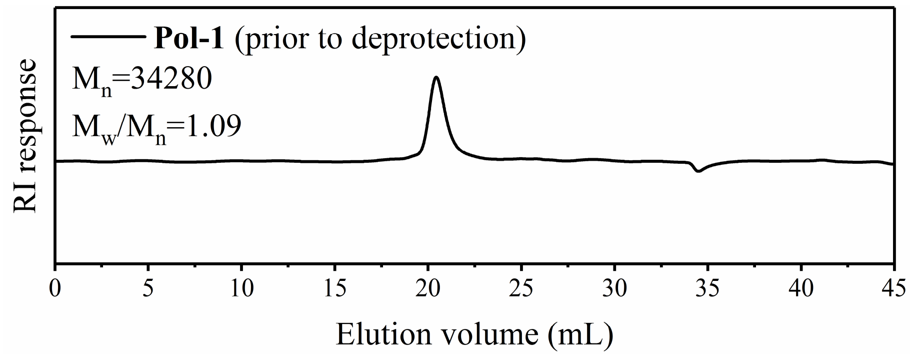

Supplementary Figure 81. GPC trace of Pol-1 at the sidechain protected stage using DMF as the mobile phase.

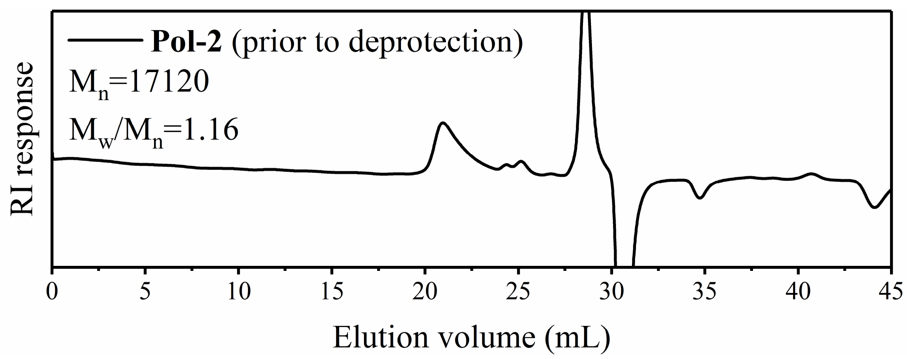

Supplementary Figure 82. GPC trace of Pol-2 at the sidechain protected stage using DMF as the mobile phase.

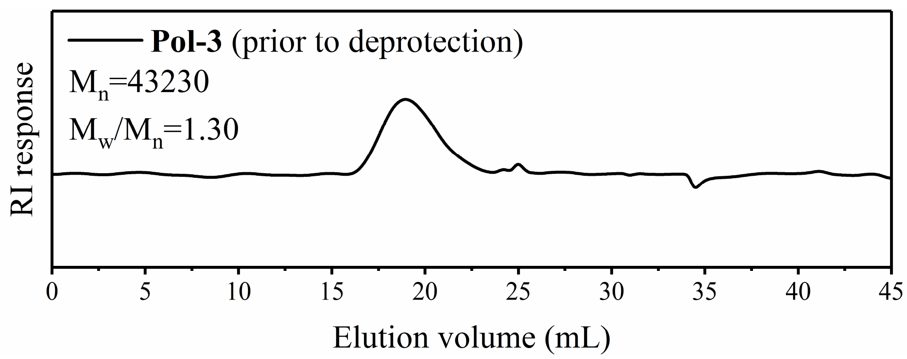

Supplementary Figure 83. GPC trace of Pol-3 at the sidechain protected stage using DMF as the mobile phase.

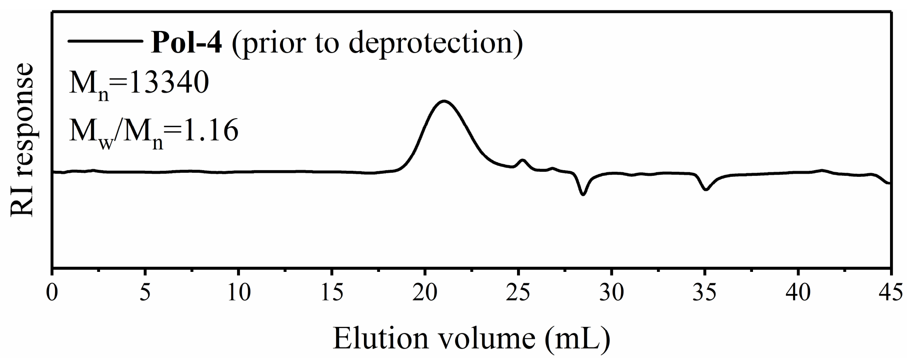

Supplementary Figure 84. GPC trace of Pol-4 at the sidechain protected stage using DMF as the mobile phase.

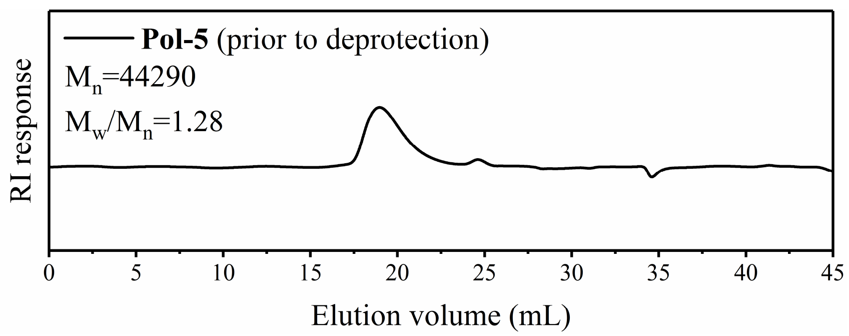

Supplementary Figure 85. GPC trace of Pol-5 at the sidechain protected stage using DMF as the mobile phase.

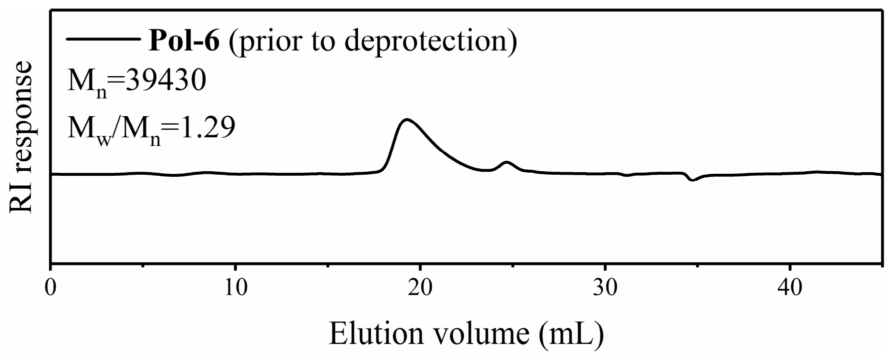

Supplementary Figure 86. GPC trace of Pol-6 at the sidechain protected stage using DMF as the mobile phase.

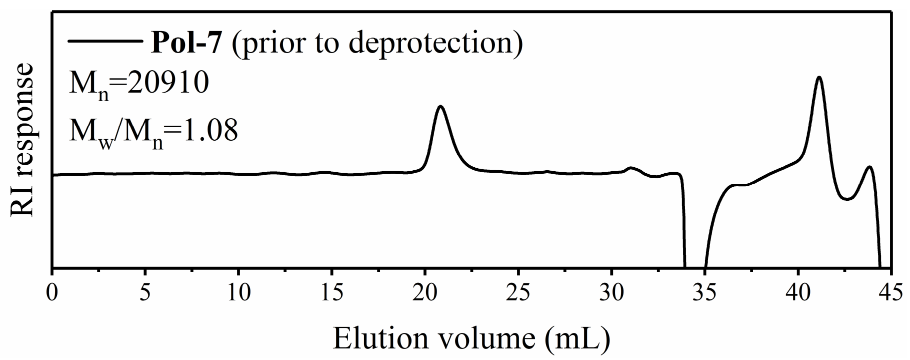

Supplementary Figure 87. GPC trace of Pol-7 at the sidechain protected stage using DMF as the mobile phase.

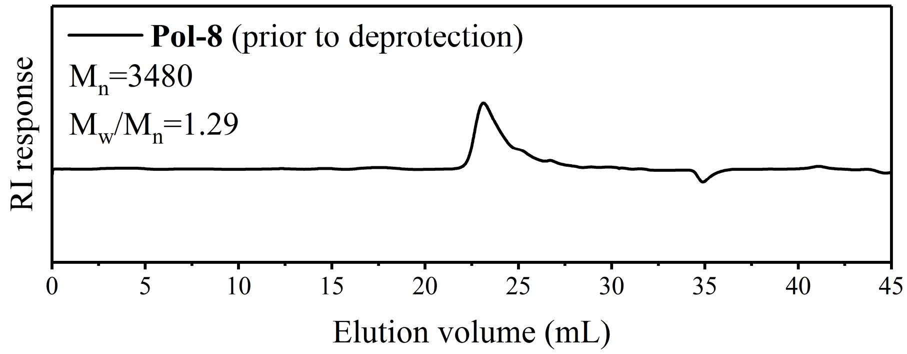

Supplementary Figure 88. GPC trace of Pol-8 at the sidechain protected stage using DMF as the mobile phase.

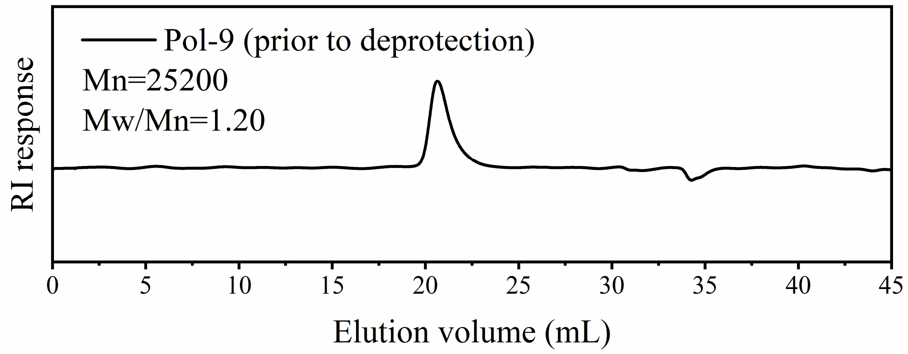

Supplementary Figure 89. GPC trace of Pol-9 at the sidechain protected stage using DMF as the mobile phase.

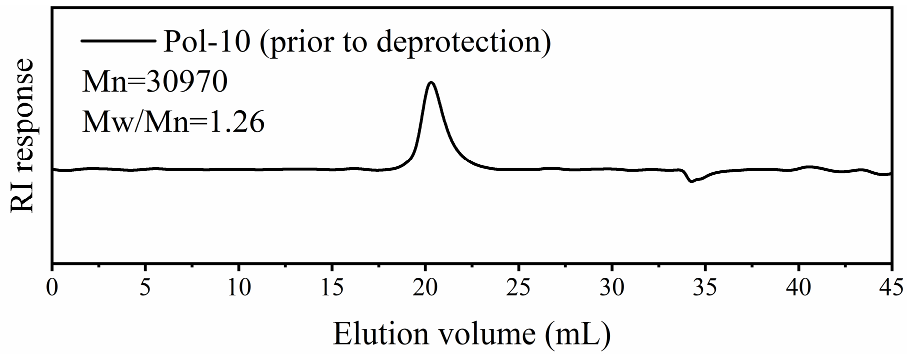

Supplementary Figure 90. GPC trace of Pol-10 at the sidechain protected stage using DMF as the mobile phase.

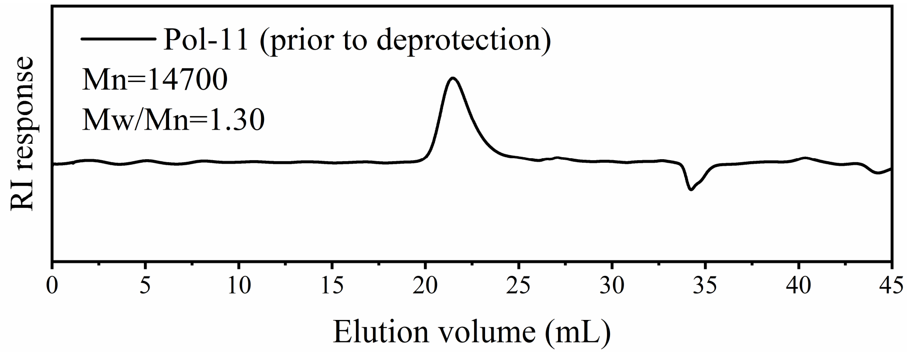

Supplementary Figure 91. GPC trace of Pol-11 at the sidechain protected stage using DMF as the mobile phase.

Supplementary Tables

Supplementary Table 1. Characterization of polymers.

Polymer	^{1}H-NMR	GPC characterization (prior to deprotection)		
	DP[a]	M_n[b] (kDa)	$Đ$[c]	DP[a]
Pol-1	ND[d]	34.3	1.09	209
Pol-2	95	17.1	1.16	139
Pol-3	ND[d]	43.2	1.30	265
Pol-4	117	13.3	1.26	109
Pol-5	177	44.3	1.28	160
Pol-6	93	39.4	1.29	142
Pol-7	116	20.9	1.08	125
Pol-8	16	3.5	1.29	16
Pol-9	ND[d]	25.2	1.20	152
Pol-10	ND[d]	31.0	1.26	188
Pol-11	ND[d]	14.7	1.30	86

[a]DP is the degree of polymerization; [b]M_n is the number average molecular weight; [c]$Đ$ is the polydispersity index. [d]ND means the value cannot be obtained by ^{1}H-NMR.

Supplementary Table 2. Rupture force of the adhesive moieties measured using SMFS.

Adhesive moiety	DbaYKY	YY	YKY	KYK	KKYY
Median (pN)	223.0	81.7	163.5	172.2	202.8
Mean (pN)	251.9	97.2	210.0	203.3	246.0
P#	-	<0.001	<0.001	<0.001	0.63

#Significant difference analysis between DbaYKY and other adhesive moieties in the table was determined by two-tailed t-test.

Supplementary Table 3. SMFS results of samples from two different batches.

		Rupture force (pN)				
		DbaYKY	YY	YKY	KYK	KKYY
Median	Batch 1	223.0	81.7	163.5	172.2	202.8
	Batch 2	240.6	58.2	151.4	167.3	227.8
Mean	Batch 1	251.9	97.2	210.0	203.3	246.0
	Batch 2	260.2	70.8	171.9	192.1	272.8

Reference

1 Liu, J., Zhang, D., Jiang, W. & Liu, R. Simple and Cost-Effective Synthesis of Fmoc-DOPA(acetonide)-OH. *Chinese J. Org. Chem.* **40**, 2543, (2020).

2 Liu, Z., Hu, B. H. & Messersmith, P. B. Convenient Synthesis of Acetonide Protected 3,4-Dihydroxyphenylalanine (DOPA) for Fmoc Solid-Phase Peptide Synthesis. *Tetrahedron Lett.* **49**, 5519-5521, (2008).

3 Liu, R., Masters, K. S. & Gellman, S. H. Polymer chain length effects on fibroblast attachment on nylon-3-modified surfaces. *Biomacromolecules* **13**, 1100-1105, (2012).

4 Wu, Y. *et al.* Lithium hexamethyldisilazide initiated superfast ring opening polymerization of alpha-amino acid N-carboxyanhydrides. *Nat. Commun.* **9**, 5297, (2018).

5 Liu, R., Chen, X., Gellman, S. H. & Masters, K. S. Nylon-3 polymers that enable selective culture of endothelial cells. *J. Am. Chem. Soc.* **135**, 16296-16299, (2013).
