# Supplementary material for: A sandcastle worm-inspired strategy to functionalize wet hydrogels
Source: Nat Commun. 2021 Nov 3;12:6331. doi: 10.1038/s41467-021-26659-0 (PMC8566497; doi:10.1038/s41467-021-26659-0)
Supplement: Supplementary file 3 — Reporting Summary [file 41467_2021_26659_MOESM3_ESM.pdf]

nature portfolio

Corresponding author(s): Runhui Liu

Last updated by author(s): Sep 23, 2021

Reporting Summary

Nature Portfolio wishes to improve the reproducibility of the work that we publish. This form provides structure for consistency and transparency in reporting. For further information on Nature Portfolio policies, see our Editorial Policies and the Editorial Policy Checklist.

Statistics

For all statistical analyses, confirm that the following items are present in the figure legend, table legend, main text, or Methods section.

n/a	Confirmed	
☐	☒	The exact sample size (*n*) for each experimental group/condition, given as a discrete number and unit of measurement
☐	☒	A statement on whether measurements were taken from distinct samples or whether the same sample was measured repeatedly
☐	☒	The statistical test(s) used AND whether they are one- or two-sided<br>*Only common tests should be described solely by name; describe more complex techniques in the Methods section.*
☒	☐	A description of all covariates tested
☐	☒	A description of any assumptions or corrections, such as tests of normality and adjustment for multiple comparisons
☐	☒	A full description of the statistical parameters including central tendency (e.g. means) or other basic estimates (e.g. regression coefficient) AND variation (e.g. standard deviation) or associated estimates of uncertainty (e.g. confidence intervals)
☐	☒	For null hypothesis testing, the test statistic (e.g. *F*, *t*, *r*) with confidence intervals, effect sizes, degrees of freedom and *P* value noted<br>*Give P values as exact values whenever suitable.*
☒	☐	For Bayesian analysis, information on the choice of priors and Markov chain Monte Carlo settings
☒	☐	For hierarchical and complex designs, identification of the appropriate level for tests and full reporting of outcomes
☒	☐	Estimates of effect sizes (e.g. Cohen's *d*, Pearson's *r*), indicating how they were calculated

Our web collection on statistics for biologists contains articles on many of the points above.

Software and code

Policy information about availability of computer code

Data collection

AVANCE III 400 spectrometer (400 MHz) or Ascend 600 spectrometer (600 MHz) was operated with TopSpin software (version: 3.1);
Waters XEVO G2 TOF mass spectrometer with MassLynxTM software (version: 4.1);
Waters GPC instrument was operated with Breeze 2 software (version: 6.20.00.00);
Thermo Scientific K-Alpha XPS System with Avantage software (version: 5.948);
Nanoscope VIIIa microscope system (Bruker, USA) was operated with NanoScope Analysis software (version: 1.8);
Microplate reader (Molecular Devices SpectraMax M2 precision) was operated with SoftMax Pro software (version: 6.5.1);
Microscope (Nikon Eclipse Ti-S; Japan) was operated with NIS Elements software (version: 4.60);
Confocal microscope (Leica TCS SP8) was operated with Leica Application Suite X software (version: 3.5.5.19976);
Typhoon TRIO Variable Mode Imager with Typhoon Scanner Control software (version: 5.0.0409.0700);
SHIMADZU LC 20AR HPLC System was operated with LabSolutions software (version: 5.90);
UV1800 Series UV-vis spectrophotometer with UV probe software (version: 2.43);
Matrix-assisted laser desorption/ionization time-of-flight (MALDI-TOF) mass spectra was operated with Data Explorer software (version: 4.3);
Pannoramic 250/MIDI scanner equipped with the CaseViewer software (version: 2.0).

Data analysis	Excel (version: 16.0.11727.20222);<br>Origin (version: 8.5.0 SR1 b161);<br>MestReNova (version: 14.0.0-23239);<br>XPS Peak Fit (version: 4.1);<br>CaseViewer (version: 2.0);<br>Adobe Photoshop CC (version: 20.0.6);<br>ImageQuant LAS 4000 Control software (version: 8.1).

For manuscripts utilizing custom algorithms or software that are central to the research but not yet described in published literature, software must be made available to editors and reviewers. We strongly encourage code deposition in a community repository (e.g. GitHub). See the Nature Portfolio guidelines for submitting code & software for further information.

Data

Policy information about availability of data

All manuscripts must include a data availability statement. This statement should provide the following information, where applicable:

- Accession codes, unique identifiers, or web links for publicly available datasets
- A description of any restrictions on data availability
- For clinical datasets or third party data, please ensure that the statement adheres to our policy

Data that support the findings detailed in this study are available in the Supplementary Information and this article. Data is available from the corresponding author. The Source data underlying Figures. 3b, c, d, e, f, 4b, d, 7c, Supplementary Figures. 4, 6b, c, d, 8b, c, d, e and 9 are provided as a Source data file.

Field-specific reporting

Please select the one below that is the best fit for your research. If you are not sure, read the appropriate sections before making your selection.

[x] Life sciences [ ] Behavioural & social sciences [ ] Ecological, evolutionary & environmental sciences

For a reference copy of the document with all sections, see nature.com/documents/nr-reporting-summary-flat.pdf

Life sciences study design

All studies must disclose on these points even when the disclosure is negative.

Sample size	No sample size calculation was performed in advance. Samples size was determined based on the reproducibility of the experiments to ensure confident statistical analyses.
Data exclusions	No data were excluded from the analyses in this study.
Replication	In vitro experiments were reliably repeated at least two times. In vivo wound healing experiments were performed once. For in vivo experiment, reproducibility was verified by similar results for individual animal within the group. IACUC approval requires that the minimum possible number of animals be used that yield statistically significant results.
Randomization	Randomization used for in vitro and in vivo study.
Blinding	Cell adhesion experiments were blinded, other experiments were not blinded because the first author who prepared the samples also participating these experiments.

Reporting for specific materials, systems and methods

We require information from authors about some types of materials, experimental systems and methods used in many studies. Here, indicate whether each material, system or method listed is relevant to your study. If you are not sure if a list item applies to your research, read the appropriate section before selecting a response.

Materials & experimental systems

n/a	Involved in the study	
[ ]	[x]	Antibodies
[ ]	[x]	Eukaryotic cell lines
[x]	[ ]	Palaeontology and archaeology
[ ]	[x]	Animals and other organisms
[x]	[ ]	Human research participants
[x]	[ ]	Clinical data
[x]	[ ]	Dual use research of concern

Methods

n/a	Involved in the study	
[x]	[ ]	ChIP-seq
[x]	[ ]	Flow cytometry
[x]	[ ]	MRI-based neuroimaging

Antibodies

Antibodies used	Rabbit anti-vinculin/AF555 polyclonal antibody (Beijing Biosynthesis Biotechnology Co., Ltd. Catalog no: bs-6640R-AF555).
Validation	Rabbit anti-vinculin/AF555 polyclonal antibody:<br>Species Reactivity: Human, Mouse, Rat, (predicted: Chicken, Dog, Pig, Cow, Horse, Rabbit.)<br>Application: Immunofluorescence.

Eukaryotic cell lines

Policy information about cell lines

Cell line source(s)	NIH 3T3 fibroblast cells (ATCC CRL-1658) were obtained from the Cell Bank of the Chinese Academy of Sciences (Shanghai, China).
Authentication	NIH 3T3 fibroblast cells (ATCC CRL-1658) were not authenticated by us repeatedly.
Mycoplasma contamination	Cell lines were not tested by us repeatedly for mycoplasma contamination.
Commonly misidentified lines<br>(See ICLAC register)	No commonly misidentified lines were used.

Animals and other organisms

Policy information about studies involving animals; ARRIVE guidelines recommended for reporting animal research

Laboratory animals	Female Sprague-Dawley rats (SD rats, 8 weeks old) used in this study.
Wild animals	The study did not involve wild animals.
Field-collected samples	The study did not involve field-collected samples.
Ethics oversight	All experiments were performed with the guidelines and approval of the Animal Care and Ethics Committee of East China University of Science and Technology.

Note that full information on the approval of the study protocol must also be provided in the manuscript.
