# Supplementary material for: Author Correction: A sandcastle worm-inspired strategy to functionalize wet hydrogels
Source: Nat Commun. 2026 Aug 18;17:8576. doi: 10.1038/s41467-026-76868-8 (PMC13487143; doi:10.1038/s41467-026-76868-8)
Supplement: Supplementary file 1 [file 41467_2026_76868_MOESM1_ESM.pdf]

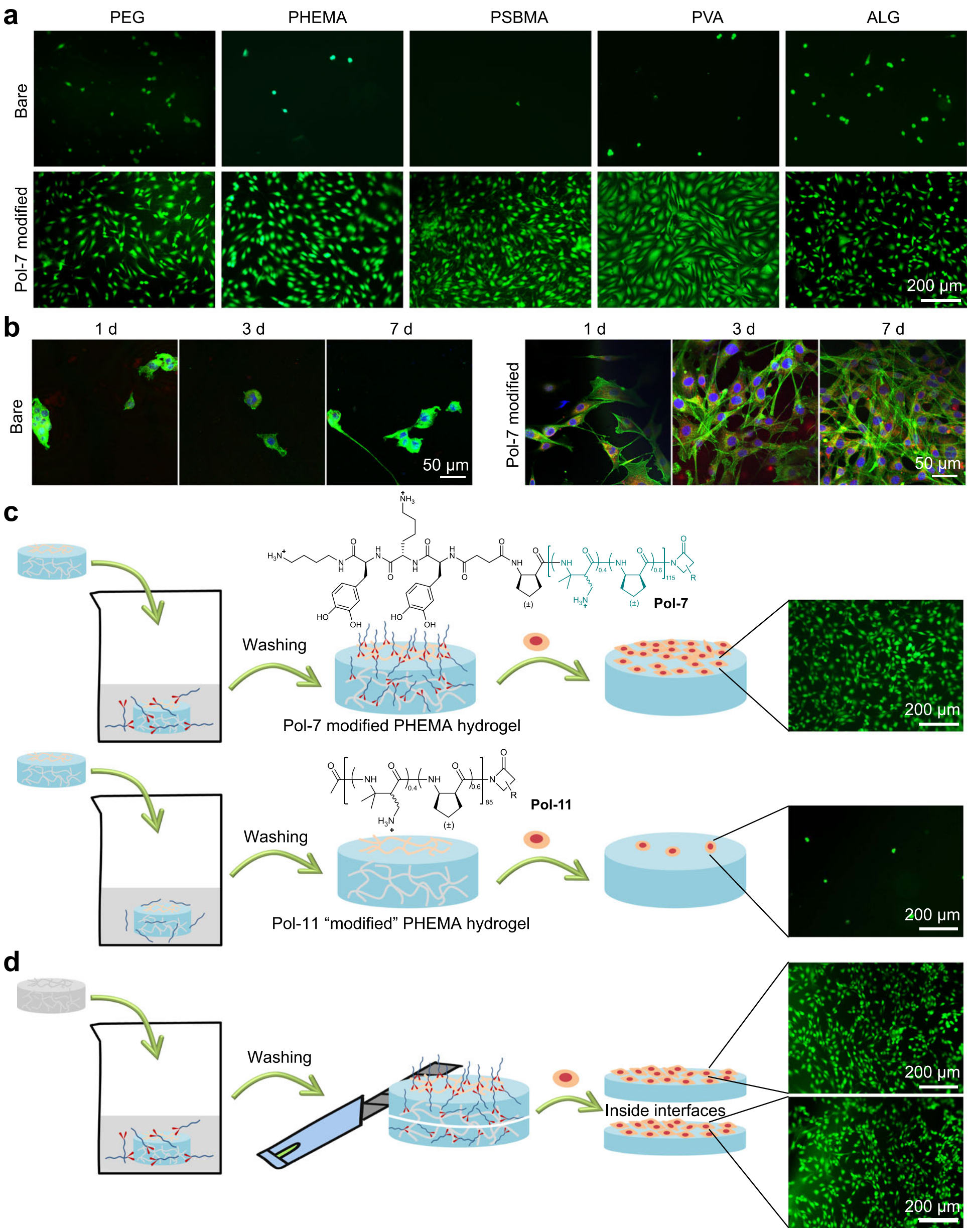

Fig. 6 One-step modification of hydrogels to have cell adhesive function. a Fluorescent microscopy images of 3T3 fibroblast cell adhesion to the bare hydrogels and functionalized hydrogels (PHEMA, PEG, PSBMA, PVA, and ALG) with DbaYKY terminated Pol-7 after cell seeding for 1 day. **b** Fluorescence confocal microscope images (green, actin; red, vinculin; blue, nucleus) of NIH 3T3 fibroblast cells on bare PEG hydrogels and Pol-7 modified PEG hydrogels after cell seeding for 1 day, 3 days, and 7 days. Scale bar: 50 μm. **c** Demonstration of cell adhesion on Pol-7 (with adhesive DbaYKY) modified PHEMA hydrogel and Pol-11 (without adhesive DbaYKY) "modified" PHEMA hydrogel, with polymer modification starting from swelling hydrogels. **d** Demonstration on direct modification of Pol-7 to both the outside and inside of the freeze-dried PHEMA hydrogels, as shown for the inside interfaces by cutting the PHEMA hydrogels from the middle. Cells were treated with LIVE/DEAD staining using calcein AM (green) and ethidium homodimer-1 (red), followed by imaging under a fluorescence microscope. Scale bar: 200 μm.
